# Extending El Sayed's Rule by ^1^(n,π*)–^3^(n,π*) Rotation via Intramolecular Secondary Interaction Enables Room‐Temperature Circularly Polarized Phosphorescence in Solution

**DOI:** 10.1002/anie.8360226

**Published:** 2026-09-23

**Authors:** Oscar Charpentier, Marie Guesdon, Catherine Demangeat, Maher Hojorat, Joseph Young, Emrys W. Evans, Vincent Dorcet, Nicolas Galland, Denis Jacquemin, Ludovic Favereau

**Affiliations:** ^1^ ISCR – UMR CNRS 6226 Université de Rennes, CNRS Rennes France; ^2^ Department of Chemistry Swansea University Centre for Integrative Semiconductor Materials Swansea UK; ^3^ CEISAM UMR 6230 Nantes Université CNRS Nantes France; ^4^ Institut Universitaire de France (IUF) Paris France

**Keywords:** non‐covalent intramolecular interactions, organic chiral emitters, room temperature circularly polarized phosphorescence, spin orbit coupling

## Abstract

The development of purely organic chiral room temperature (RT) phosphorescent emitters is in the limelight. However, the key parameters governing the polarized luminescence are difficult to predict and rationalize since the phosphorescence emission is rarely obtained in solution, despite the introduction of heavy atoms (Br, I) or carbonyl groups, and engineering of total angular momentum, for example, singlet/triplet states corresponding to n‐π*/π‐π* transitions (or vice‐versa), as prescribed by El‐Sayed's rule. Here, we show that intramolecular secondary interactions can induce an efficient ISC between formally identical ^1^(n,π*) and ^3^(n,π*) states in chiral thionated phthalimide derivatives. This unprecedented type of inter‐system crossing (ISC) extends the usual thinking of the El‐Sayed principle and enables the realization of circularly polarized (CP) RT phosphorescence (RTP) in solution with *g*_lum_ of up to 4 × 10^−3^. These results bring additional molecular design guidelines to reach CP‐RTP in solution and offer new insights into the subtle relationships between excited states of different spin multiplicities that enable higher CP phosphorescence intensity.

## Graphical abstract

Weak but skilled: Our results highlight the crucial role of intramolecular secondary interactions to induce an efficient ISC between formally identical ^1^(n,π*) and ^3^(n,π*) states in chiral thionated phthalimide derivatives. This unprecedented type of inter‐system crossing (ISC) extends the usual thinking of the El‐Sayed principle and enables the obtention of circularly polarized (CP) RTP in solution with *g*_lum_ of up to 4 × 10^−3^.

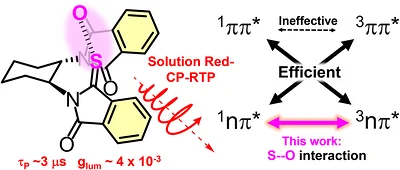

## 1 Introduction

Managing triplet excited states in purely organic molecules is of fundamental importance for several research lines, including optoelectronics, non‐linear optics, photocatalysis, photodynamic therapy, and bioimaging [1, 2, 3, 4, 5]. Recently, studies have revealed that organic triplets could contribute to charge‐carrier generation [6], act as reactive intermediates in enantioselective photo‐cycloadditions [7, 8], or provide oxygen‐sensitive contrast in bioimaging [9]. Moreover, precise control over triplet sublevels is emerging as a critical requirement for quantum information technologies and artificial photosynthesis [10, 11]. Since the seminal works of Kasha, El‐Sayed, and Kearns in the 60s [12, 13], the formation of triplet states in organic molecules has been understood almost exclusively through intersystem crossing (ISC), a spin‐forbidden transition typically taking place from the *S_1_* excited state and often remaining inefficient due to the weak spin‐orbit coupling (SOC) associated with light atoms (1 cm^−^^1^ or less). This limited accessibility of triplet states in organic compounds can be partially circumvented through two well‐known design principles: [2] (i) introducing heavy atoms (Br, I) to increase SOC and enhance ISC, and (ii) ensuring conservation of total angular momentum, for example, obtaining singlet/triplet states corresponding to n‐π*/π‐π* transitions (or vice‐versa), as prescribed by El‐Sayed's rule [12, 14, 15, 16, 17]. Along with these electronic features, providing the phosphors with a rigid environment is also crucial for observing emission, owing to the suppression of molecular motions and the diffusion of triplet quenchers, such as molecular oxygen [18, 19, 20, 21]. Following these general guidelines, several types of organic compounds have been able to show RTP, with photoluminescence quantum yield (PLQY) up to 80% and lifetimes ranging from milliseconds to several seconds [22]. In the reported examples, intermolecular interactions within crystals, or secondary interactions of the organic emitters with the matrix, play a crucial role in promoting efficient phosphorescence [23, 24, 25, 26, 27, 28, 29]. However, the joint influence of the surrounding matrix and/or intermolecular interactions on the excited‐state dynamics remains difficult to deconstruct, because in nearly all reported examples phosphorescence has only been accessible in the solid state [30, 31, 32, 33, 34, 35, 36, 37].

This represents a key challenge in this field and highlights the still limited fundamental understanding of the RTP process in organic systems. To date, phosphorescence in fluid media has indeed been observed for a very limited number of molecular designs, involving benzophenone [38, 39] or 1,2‐diketones units [40, 41], and more recently the thiadiazole moiety [42, 43, 44] (Scheme 1). These too few examples preclude establishing systematic structure‐property relationships for this phenomenon in a non‐rigid environment. We recently reported that hydrogen bonding can modify the nature of the triplet excited state in chiral organic emitters by tuning the n‐π*/π‐π* balance and allowing different mixing between the singlet n‐π* and π‐π* excited states, ultimately impacting the intensity of RTP. These results provide one of the rare examples of CP‐RTP in fluid media for purely organic systems [9, 45], and offer new insights into the potential of ‘electric‐dipole forbidden’ phosphorescence [45, 46], for achieving more intense CPL than classical spin‐allowed fluorescence of chiral emitters [47, 48]. While providing valuable insights regarding the importance of considering secondary interactions for designing efficient organic RTP, the inter‐/intra‐molecular character of these interactions hampers a clear molecular‐level rationalization.

**SCHEME 1:**
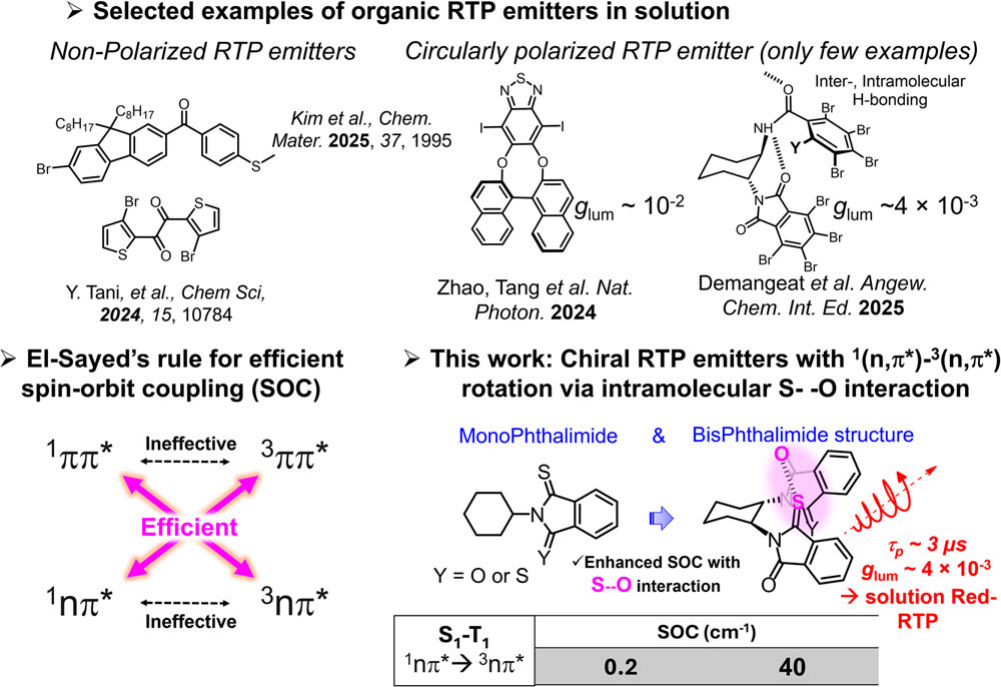
Chemical structures of selected phosphorescent emitters in fluidic medium (top) and of the thionated phthalimide phosphorescent emitters investigated in this study (bottom), with an emphasis on the key findings regarding the obtained CP‐RTP in solution.

Here, we report thionated chiral bisphthalimide derivatives able to display efficient phosphorescence emission in solution, notably due to intramolecular secondary interaction between the lone pair of the sulfur atom and the adjacent carbonyl group. Interestingly, this non‐covalent interaction circumvents the classical El Sayed principle: efficient ISC occurs between singlet and triplet states of the same n‐π* character. Indeed, detailed photophysical experiments and theoretical modeling show a dramatical enhancement of the SOC value by more than a hundred‐fold factor between S_1_ and T_1_ for the bisphthalmimide compounds compared to their mono counterpart, clearly pointing out the importance of non‐covalent interaction. In addition, using chirality as a design element, we take advantage of the unique abilities of chiroptical spectroscopies, namely electronic circular dichroism (ECD) and circularly polarized luminescence (CPL), to probe the involved key singlet and triplet excited states. These experiments notably allow us to distinguish an *S*_0_ → *T*_1_ absorption band with an oscillator strength four times more intense than the *S*_0_ → *S*_1_ one, as well as an intense CP microsecond phosphorescence at the molecular level and at room temperature in solution, with a promising *g*_lum_ of up to 4 × 10^−^^3^. Taken together, these results highlight the potential role of secondary interactions in manipulating the SOC of purely organic emitters, affording a distinguishable *S*_0_ → *T*_1_ optical excitation, and one of the rare examples of CP‐RTP in fluid media for purely organic systems.

## 2 Results and Discussion

### 2.1 Synthesis and Characterizations

The synthesis of the thionated compounds has been performed following ones previously developed for phtalimide and other rylene derivatives [49, 50, 51], by reacting enantiopure (*RR*)‐ or (*SS*)‐cyclohexylebisphthalimide starting materials with an excess of the Lawesson reagent (Figure 1). Following this synthetic pathway, the mono‐ and bis‐thionated compounds **5** and **6** were obtained in 11% and 34% yield, respectively. As model compounds, the mono‐phthalimide mono‐ and bis‐thionated compounds **2** and **3** were also obtained in 10% and 81% of yield, respectively (see the SI for details). In addition to the bisphthalimide **5** and **6**, we unexpectedly isolate a [5]‐helicene derivative **7** in 43% yield. Indeed, the obtained structure involves a new double bond between the two phthalimide units, with external thiocarbonyl groups (Figure 1 and Figure S2 for details about its x‐ray structure). We presume that the formation of this compound occurs after forming the bisthionated one in the course of the reaction. Despite its resemblance to an intramolecular thia‐Paternò^_^Büchi‐like reaction [52, 53, 54, 55, 56, 57, 58], our main hypothesis is that an intermediate dithietane is formed, which rapidly evolved to the newly obtained compound [59, 60]. In the course of our structural and optoelectronic characterizations, we noticed a weak photostability for this compound, which is also not luminescent in contrast to **5** and **6**. In the context of this study, we therefore focused our effort on the luminescent **5** and **6** compounds.

**FIGURE 1:**
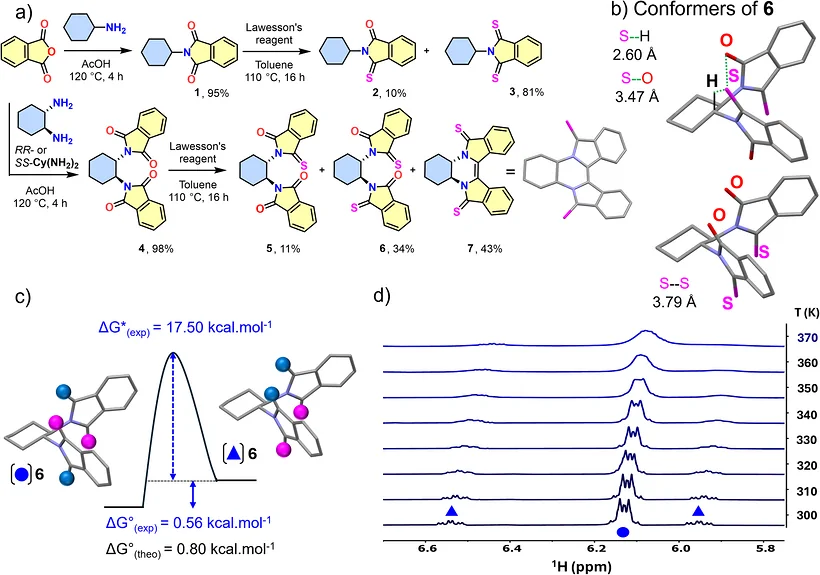
(a) Synthetic pathway for the synthesis of enantiopure **5**, **6**, and **7** compounds, with the X‐structure of the latter. (b) X‐ray structure of the conformers obtained for **6**, with (c) and (d) thermodynamic data relative to their equilibrium extracted from VT‐^1^H NMR of **6** (blue) between 300 and 370 K (400 MHz, toluene‐d_6_).

These enantiopure **5** and **6** derivatives were fully characterized by both NMR spectroscopy and mass spectrometry, displaying characteristic signals of both phthalimide and cyclohexane units in the aromatic and aliphatic parts of the ^1^H spectrum, respectively. While a characteristic multiplet at 4.59 ppm can be observed for the two protons on the carbon atoms next to the nitrogen ones for the monophthalimide system **2**, additional peaks are observed for the bisphthalimide bearing one or two sulfur atoms, **5** and **6**, respectively. Indeed, a triplet of doublet signal can be distinguished at 5.73 ppm for **5**, with an integration of 0.15 in CDCl_3_ at 298 K. Despite its symmetric structure, similar additional NMR signals are also observed for **6** bearing one sulfur atom on each phthalimide unit, even after several purification steps (crystallization, chiral HPLC). Owing to the bigger size of the sulfur atom in comparison to the oxygen one, we envisioned the occurrence of conformational exchange in solution and at room temperature [61]. To further probe such a hypothesis, we performed variable temperature (VT) NMR experiments in toluene‐d_6_ for both bisphthalimide **5** and **6**.

As depicted in Figure 1 for **6**, the small triplet of doublet NMR signals at 5.90 and 6.50 ppm become broader and closer to the major signal at 6.14 ppm during the VT NMR experiment. The observed shifts were used to estimate the relative energy of the two forms as well as the activation barrier for their interconversion, using the Eyring equation. Corresponding **ΔG^0^** and ΔG^*^ values of 0.56 and 17.5 kcal mol^−1^, respectively, were obtained for bisphthalimide **6**, slightly higher than the monothionated system **5** (Figures S4, S5). These results were further confirmed by theoretical calculations, which also indicate the presence of several conformers for both bisphthalimide **5** and **6**, with values in complete agreement with the experimental ones (Figure 1b and Figures S38–S40 in the Supporting Information).

Finally, x‐ray structure of **6** has been obtained and shows the two isomers in the crystalline unit cell (Figure 1). It crystallizes into the P_21_ space group, with the expected equatorial arrangement of the phthalimide cores. Close contact of 2.60 Å between the sulfur atom and the axial hydrogen from the cyclohexyl scaffold can be observed, as well as a distance close to the sum of the Van der Waals radii between the sulfur and oxygen atoms from the opposite phthalimide core of 3.47 Å (the sum of the radii of the S and O atoms is 3.32 Å). In the crystal packing of the *face‐to‐face* conformer, a distance of 3.79 Å is observed between the two sulfur atoms. This distance is a bit higher than the sum of the van der Waals radii of the two sulfur atoms (3.60 Å), making significant intramolecular S–S contact unlikely.

### 2.2 Photophysical and Chiroptical Properties in Solution

The optical and photophysical properties of these compounds were firstly investigated in diluted chloroform solution, resulting in a similar UV‐vis absorption signature for the mono‐thionated phthalimide **2** and the bisphthalimide ones **5** and **6**. Taking **2** as an example, the UV/Vis spectra show an intense absorption band at approximately 290 nm (*ε* ∼ 1.6 × 10^4^ M^−1^cm^−1^) and a smaller one at 330 nm with a noticeable shoulder at 360 nm (*ε* = 7.5 × 10^3^ M^−1^cm^−1^) that ends around 400 nm. The only difference is the intensity of the absorption, which is expectedly higher in the case of the bisphthalimide derivatives (Figure 2a). For all compounds, an additional absorption band is observed when zooming between 450 and 570 nm. The latter is very weak (*ε* ∼ 30 M^−1^cm^−1^) for **2** and undergoes a slight redshift and a significant hyperchromic shift when going to the bisphthalimide systems (Figure 2a). Based on previous literature on thionated compounds [54, 56, 62, 63] and theoretical calculation (*vide infra*), we attributed this band to the n‐π* transition of the C═S group.

**FIGURE 2:**
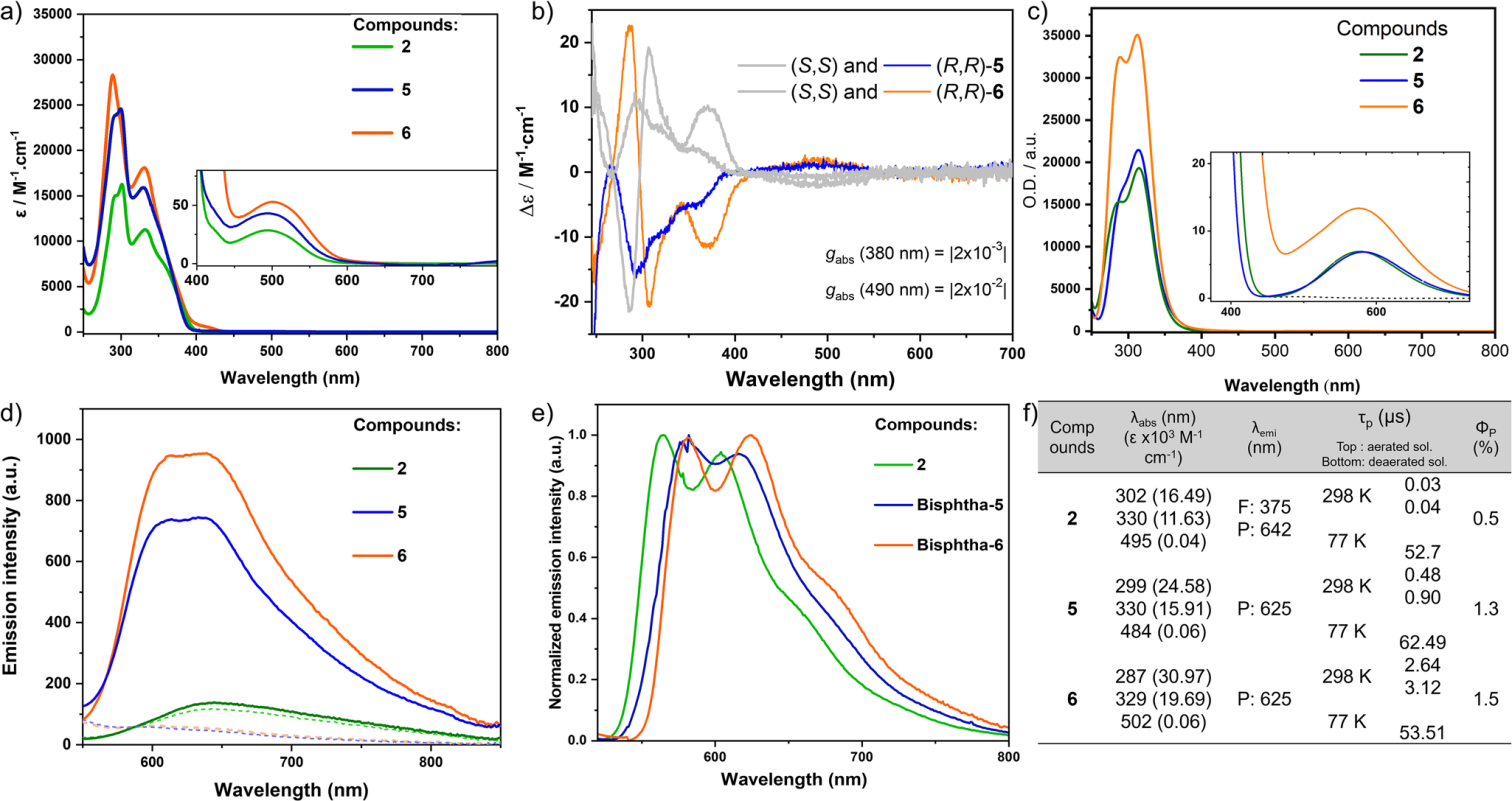
UV‐vis absorption (a), ECD (b), and theoretical absorption spectra (c) of compounds **2**, **5,** and **6** in chloroform solution (10^−5^ M), with their corresponding emission spectra in air (dotted lines) and under degassed (solid lines) solution at room temperature (d), and at 77 K (e) in chloroform solution (10^−5^ M, *λ*_exc_ = 330 nm). (f) Photophysical data of the compounds recorded in chloroform at 298 K and at 77 K.

Electronic circular dichroism (ECD) spectra of the chiral bisphthalimide **5** and **6** afford more insights regarding the impact of having one or two thionated phthalimide units. Indeed, (*R*,*R*)‐**5** shows a negative signal at 250 nm, followed by a broad and negative band between 270 and 390 nm with a maximum at 300 nm, and finally a lower positive between 400 and 550 nm. While *(R,R)*‐**6** displays the first similar transition at high energy, the other part of its spectrum appears different with a strong positive exciton coupling at 300 nm, a negative signal at 380 nm, and a similar but more intense positive band than *(R,R)*‐**5** between 400 and 550 nm. The difference observed between these ECD signatures can be explained by the less favored chiral excitonic coupling in *(R,R)*‐**5**, which involves non‐degenerate electronic dipoles from each phthalimide unit. In comparison, the exciton coupling in *(R,R)*‐**6** is more intense across the whole ECD spectra owing to the degenerate electronic dipoles, especially for the π–π* and n–π* transitions between 300 and 400 nm.^38,39,40^ It is interesting to note that ECD is much more sensitive to the forbidden n‐π* transition of the C═S group transition than classical UV‐vis absorption because of the stronger interaction between a CP light and a transition having a low electric dipole moment transition, such as this n‐π* excitation.

Further insights regarding this specific aspect and the ground and excited states of these thionated phthalimide derivatives were obtained by theoretical calculations (see the SI for details, Figures S42–S49). The TD‐DFT optical spectra for the UV‐vis and ECD features reasonably reproduce also the experimental ones (Figures S42, S43), including, importantly, the additional exciton coupling for *(R,R)*‐**6**. It is obvious from the EDD plots provided in Figures S44–S49 (see also below) that the lowest excited‐states have an n‐π* character in all compounds, as expected. One interesting feature coming out of the theoretical calculations is that for the n‐π* transition in **6**, the oscillator strength determined for the most intense triplet sublevel (2 × 10^−4^) is of similar magnitude as the corresponding singlet state (1 × 10^−4^), once SOC effects are included in the calculations.

In solution, all the compounds show a broad emission between 550 and 800 nm when excited at 330 nm. In addition, monophthalimide **2** displays an additional structured luminescence signal at higher energy between 340 and 450 nm when excited at 300 nm, which is absent for bisphthalimides **5** and **6**. This unusual behavior for organic compounds is already known for thiocarbonyl systems, which can show dual fluorescence and phosphorescence with emissions arising from S_2_ and T_1_ excited‐states, respectively [54, 55, 56, 64, 65]. Indeed, oxygen dependence of the red emission intensity, as well as the subnano‐ and micro‐second lifetimes measured at 370 and 610 nm, confirm such behavior for **2**, while compounds **5** and **6** show only phosphorescence emission. This red‐emission is related to the thiocarbonyl group, which lowers the LUMO of the compounds [49, 51, 62], in comparison to the oxygenated ones (Figure S16 [data of **4**]). This is also evidenced by the lowest reduction potentials recorded for the thionated compounds recorded in electrochemistry (Figure S17).

A key finding of the present study is the difference in emission intensity observed when going from mono‐ to bisphthalimide compounds. In solution, the photoluminescence quantum yield (PLQY) of monophthalimide **2** is at least three times smaller than that of the bisphthalimide derivatives **5** and **6**, reaching approximately 1.5% for the latter. Although these values remain very modest, they are noteworthy in the context of organic RTP emitters, especially for the red ones, which remain highly challenging to obtain even in the solid state. Indeed, the performances of organic phosphors are often difficult to improve in the red region because the phosphorescence rate is unfavorably related to the emission wavelength, that is, *k*_p_ is inversely proportional to the cube of the RTP wavelength while *k*_nr_(RT) is nearly proportional to the square of the latter) [12]. Further analysis related to these radiative and non‐radiative constants is detailed in the Supporting Information (Table S2).

In addition to their higher PLQY, compounds **5** and **6** also exhibit longer lifetimes than **2**, with values up to 3 µs for bisphthalimide **6** (Figure 2f). In thiocarbonyl‐containing systems, ISC is typically more efficient due to the presence of sulfur, which enhances SOC between singlet and triplet excited states. We underline that although sulfur is present in both mono‐ and bisphthalimide systems, the bisphthalimide architecture significantly improves phosphorescence efficiency, even though only one sulfur atom is involved in **5**.

Emission spectra recorded in dilute chloroform at 77 K (Figure 2e) display similar structured profiles, with maxima at 580, 630, and 680 nm, confirming that phosphorescence originates from the thionated phthalimide unit. While the spectral shapes remain comparable, a slight redshift of the phosphorescence onset is observed for the bisphthalimide derivatives. These results suggest a greater stabilization of the lowest triplet excited state in both **5** and **6**. This behavior is pointing to an additional factor contributing to their improved phosphorescence efficiency. Theoretical calculations performed on the optimized S_1_ geometry indeed reveal reasonably small S‐T gaps, with interestingly, significantly larger SOCs between S_1_ and T_1_ in both bisphthalimides than in monophthalimide **2** (Figure 3 and Supporting Information): the presence of an additional phthalimide unit in the two former compounds greatly aids the mixing of excited‐state characters, in turn, promoting more efficient ISC. In both **5** and **6**, we indeed determined theoretically S_1_–T_1_ SOCs of ∼40 cm^−1^, which are exceptionally large values for organic compounds, even sulfur‐containing ones. It is noteworthy that in **6**, the optimizations of the lowest excited state of both spin symmetries yield to localization of the state on one side (symmetry breaking), as illustrated in Figure 3c. From the analysis of the topology of S_1_ and T_1_ as provided by the electron density difference (EDD) in Figure 3 and Figures S44–S49, it turns out that both the lowest singlet and triplet states have clear n‐π* character for all compounds, which is in apparent contradiction with the well‐accepted El‐Sayed rule [12]. Indeed, the latter states that ISC is generally more efficient when it involves a change in the orbital type/character of the electronic transition. The physical rationale is that changing the orbital character can increase the relevant SOC matrix element, thereby facilitating the spin‐forbidden transition between singlet and triplet states.

**FIGURE 3:**
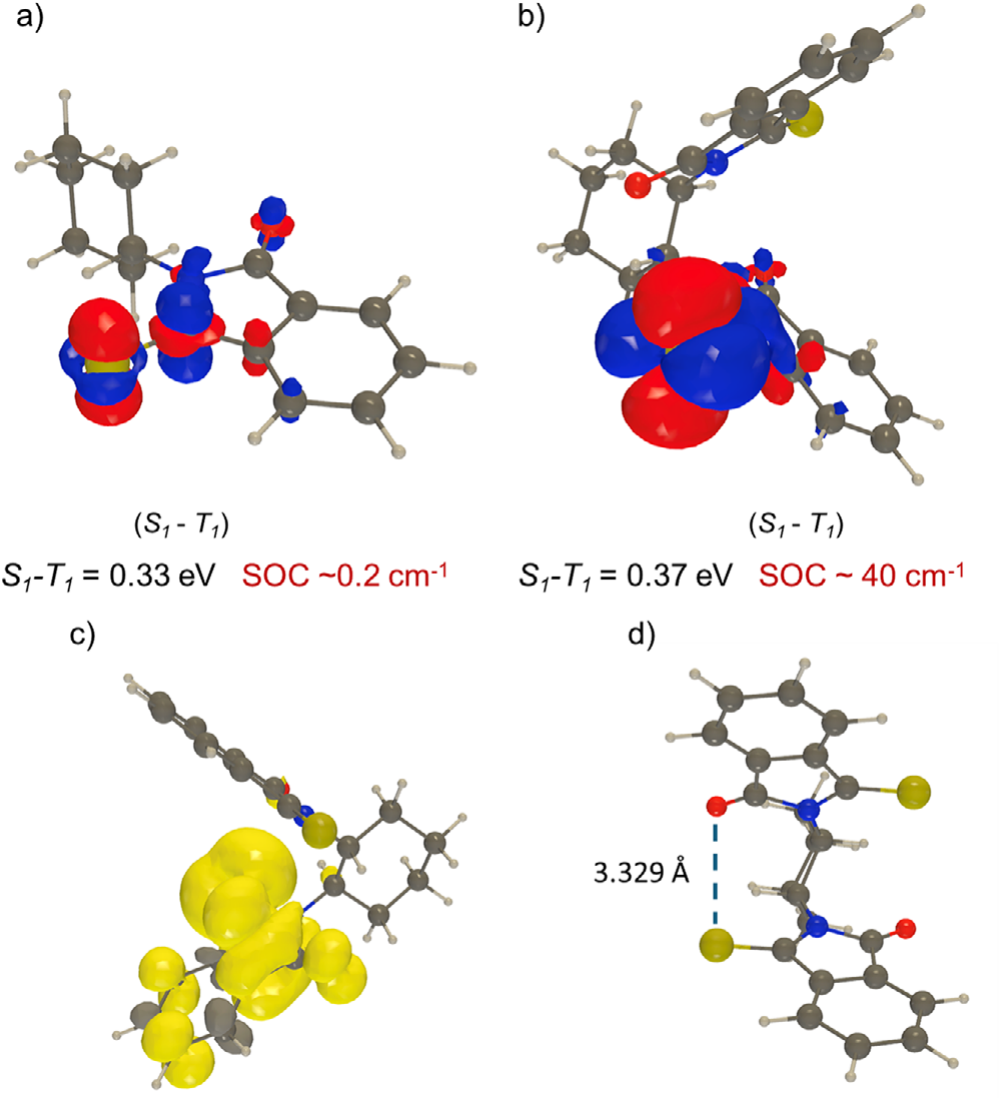
Electron density difference plot corresponding to the *S_1_–T_1_* densities on the singlet excited‐state geometry determined at PCM‐TD‐PBE0/6‐311++G(d,p) level for (a) monophthalimide **2** and (b) bisphthalimide **6**. The blue (red) regions indicate a decrease (increase) of electron density upon ISC (contour threshold: 0.0001 au), with their SOC (cm^−1^) computed between the lowest *S_1_* state and the lowest triplet state on the optimal *S_1_* geometry, with the associated S–T gaps in eV. (c) Spin difference plot (*α*–*β*) plots corresponding to the lowest triplet excited state of **6** (threshold: 0.002 au) showing localization on one side, with (d) the calculated S–O distance.

Therefore, the observed situation for compounds **5** and **6** does not constitute the classical favorable El‐Sayed situation. However, the spatial orientation of the n orbital contribution changes significantly between S_1_ and T_1_ for compounds **5** and **6**, and not really for **2** (Figure 3 and Figures S44–S49). This difference cannot readily be linked to a large structural rearrangement because the EDDs are compared at a fixed S_1_‐optimized geometry, with, in addition, the phthalimide chromophore remaining geometrically similar for all mono‐ and bisphthalimide compounds.

We therefore assign the observed effect to electronic communication between the two phthalimide units, and notably through a non‐covalent interaction with the opposite oxygen atom. Such an interaction sems to induce a change in orbital organization that can affect the electronic structure and SOC between the singlet and triplet states and is therefore consistent with the electronic rationale underlying El‐Sayed's rule. In this sense, our results suggest that the enhanced ISC behavior is associated not with a simple geometrical rotation, but with an electronically driven redistribution of the excited‐state wavefunction. This argument is in line with the calculated SOC matrix elements (Table S5). The significant difference between the orientations of the n orbitals in the singlet and triplet states for both compounds **5** and **6**, therefore, suggests a “rotation effect” justifying El‐Sayed's rule is indeed present, but very originally between two n‐π* states.

To get further information regarding this interaction, we conducted additional modeling Quantum Theory of Atoms in Molecules and Non‐Covalent Interactions  methods (QTAIM and NCI) that point toward weak interactions of predominantly electrostatic nature between the sulfur and the opposite oxygen atom, with only a minor contribution from charge transfer (Figure 3d and the Supporting Information). Between **5** and **6**, this intramolecular interaction appears to be more effective in **6**.

The obtained novel chiral emitters enable us to investigate the still rare CPL of chiral RTP in solution. As depicted in Figure 4, reliable mirror‐image spectra were obtained for both bisphthalimide compounds with *g*_lum_ of approximately 2 and 4 × 10^−3^ for **5** and **6**, respectively (estimated error of 2 × 10^−4^ for these measured values).

**FIGURE 4:**
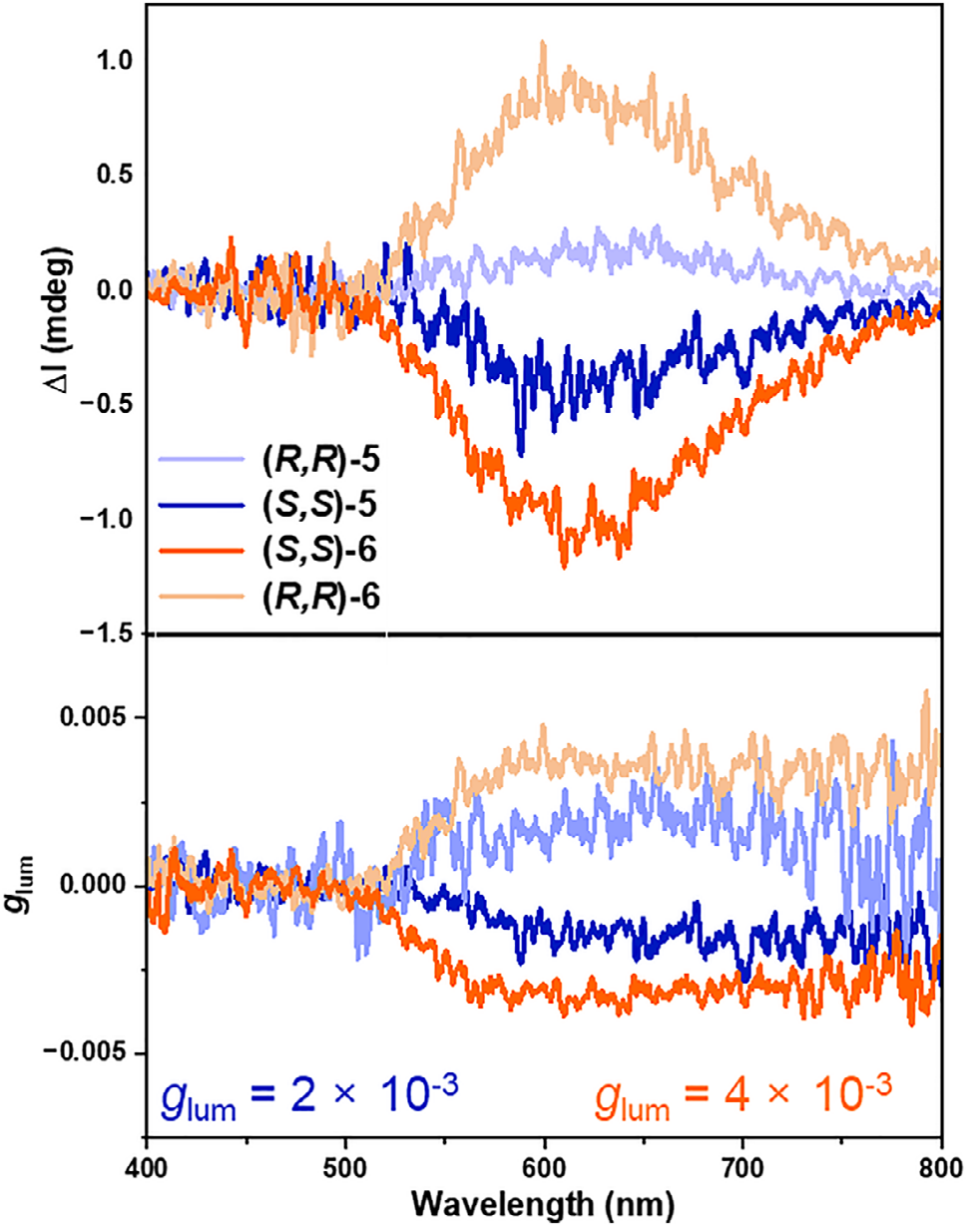
Top: Circularly polarized luminescence (CPL) and bottom: luminescence dissymmetry factor (*g*_lum_) spectra of (*R*,*R*)‐ and (*S*,*S*)‐**5** and **6** in CHCl_3_ (10^−5^ M) at 298 K under degassed condition, recorded upon excitation at *λ*_exc_ = 320 nm.

These obtained values are in line with the recently measured CP phosphorescence on aggregates of chiral phthalimide [66], as well as the usually measured intensity of CP fluorescence for other chiral diamine units [46, 47, 48]. It has been invoked that, for phosphorescent chiral organic compounds, the sign and magnitude of the *g*_lum_ value of the emission arising from a triplet state can give further information regarding its related mixing with a singlet excited‐state [66, 67]. In the present case, the magnitude and sign of the CP phosphorescence, predominantly exhibiting n–π* character, is similar to the *g*_abs_, estimated at 490 nm from the CD spectra of these compounds, which corresponds to the n‐π* transition of the C═S group. While being an estimation, it is interesting to note that ECD and CPL spectroscopies can provide strong evidence that the emitting T_1_ state couples to the singlet n–π* transition in the 450–550 nm range. These observations are consistent with the theoretical framework proposed by Blok and Dekkers more than three decades ago [67], and provide direct experimental support for the role of secondary through‐space interaction between the sulfur lone pairs and the carbonyl oxygen atoms of the opposite phthalimide unit in controlling both ISC and chiroptical responses in compounds **5** and **6**.

## 3 Conclusion

In conclusion, we have demonstrated that thionated chiral bisphthalimide derivatives constitute a powerful platform for achieving RT phosphorescence in solution through the engineering of intramolecular secondary interactions. The sulfur lone pair–carbonyl interaction introduces a modification of the excited‐state manifold, enabling a different pathway than the conventional El‐Sayed framework. In these systems, ISC is no longer restricted by orbital‐type change but instead proceeds efficiently between singlet and triplet states of the same n–π* character, as evidenced by a dramatic enhancement of SOC relative to the monophtalimide analogue. Experimental and theoretical results provide evidence of this effect, with an unusually intense S_0_ → T_1_ absorption that surpasses the S_0_ → S_1_ transition, together with unique details on the SOC from chiroptical spectroscopies. These findings establish notably a rare example of CP‐RTP from purely organic molecules under ambient conditions, with *g*_lum_ values reaching 4 × 10^−^^3^.

These results may offer new opportunities to design innovative and efficient CP‐RTP while investigating their potential in bioimaging and CP‐OLED, among other applications. Ultimately, this study provides consistent elements to achieve a better understanding of the key parameters governing the polarized luminescence.

## Author Contributions

**Oscar Charpentier**: investigation, writing – original draft, writing – review and editing. **Marie Guesdon**: investigation, writing – original draft, writing – review and editing. **Catherine Demangeat**: investigation, writing – review and editing. **Maher Hojorat**: investigation, writing – review and editing. **Joseph Young**: investigation, writing – review and editing. **Emrys W. Evans**: investigation, writing – review and editing. **Vincent Dorcet**: investigation. **Nicolas Galland**: investigation, methodology, writing – review and editing. **Denis Jacquemin**: conceptualization, funding acquisition, writing – review and editing, investigation, methodology. **Ludovic Favereau**: conceptualization, funding acquisition, investigation, writing – review and editing.

## Conflicts of Interest

The authors declare no conflicts of interest.

## Supporting information




**Supporting File**: Anie74877‐sup‐0001‐SuppMat.pdf.

## Data Availability

The data that support the findings of this study are available from the corresponding author upon reasonable request.
