# Supplementary material for: Extending El Sayed's Rule by 1(n,π*)–3(n,π*) Rotation via Intramolecular Secondary Interaction Enables Room‐Temperature Circularly Polarized Phosphorescence in Solution
Source: Angew Chem Int Ed Engl. 2026 Sep 23;65(46):e8360226. doi: 10.1002/anie.8360226 (PMC13749351; doi:10.1002/anie.8360226)
Supplement: Supplementary file 1 [file ANIE-65-e8360226-s001.pdf]

SUPPORTING INFORMATION

Extending El Sayed's Rule by $^1(n,\pi^*)$-$^3(n, \pi^*)$ Rotation via Intramolecular Secondary Interaction Enables Room-Temperature Circularly Polarized Phosphorescence in Solution

Oscar Charpentier,[a] Marie Guesdon,[a] Catherine Demangeat,[a] Maher Hojorat,[a] Joseph Young,[b] Emrys W. Evans,[b] Vincent Dorcet,[a] Nicolas Galland,[c] Denis Jacquemin,*[c,d] and Ludovic Favereau*[a]

1. General methods 2
   a) Experiment 2
   b) Theory 2
2. Synthesis monomeric and dimeric phthalimide derivatives 5
3. Crystallographic structures 7
4. Rotamers in solution 8
   a) Gibbs free energy 9
   b) Energy of activation 10
5. Photophysical properties 11
   a) Absorbance & luminescence spectra 11
   b) Phosphorescence lifetime 13
   c) Photophysical properties of 4 14
6. Cyclic voltammetry 16
7. NMR Spectra 17
8. HR-MS Spectra 24
9. Additional theoretical results 27
   a) Representation of the conformers 27
   b) Simulated spectra 28
   c) Electron Density Difference plots 29
   d) Spin Difference plots 31
   e) Computed transition energies and SOCs 32
   f) Topological analysis 33
   g) Cartesian coordinates 35

1. General methods

a) Experiment

^{1}H and ^{13}C NMR spectra were recorded at room temperature on an *AVANCE III 400 BRUKER* at Centre Régional de Mesures Physiques de l'Ouest (CRMPO), Université de Rennes. Chemical shifts δ are given in ppm, relative to an internal standard of residual deuterated solvent ($CDCl_3$: δ = 7.26 ppm for ^{1}H NMR, δ = 77.16 ppm for ^{13}C NMR); and coupling constants *J* in Hz.

High-resolution mass (HR-MS) determinations were performed at CRMPO on a Bruker MaXis 4G by ASAP (+ or -), ESI (with CH_2Cl_2/MeOH as solvents system). Experimental and calculated masses are given with consideration of the mass of the electron.

UV-Visible (UV-vis, in M^{-1} cm^{-1}) absorption spectra were recorded on a V-730 JASCO spectrophotometer. Emission spectra were measured on an FP-8350 JASCO fluorimeter with a Xenon lamp. The lifetime were recorded on a FL 920 Edinburgh fluorimeter.

Phosphorescence quantum yields Φ were measured in diluted solution using the following equation:

$$\Phi_X = \Phi_{ST}\left(\frac{\mathrm{Grad}_X}{\mathrm{Grad}_{ST}}\right)\left(\frac{\eta_X^2}{\eta_{ST}^2}\right)$$

Where the subscripts ST and X denote standard and sample respectively, Φ is the fluorescence quantum yield, Grad is the gradient from the plot of integrated fluorescence intensity vs absorbance, and η the refractive index of the solvent. Reference for fluorescence quantum yields used herein is $Ru(bpy)_3^{2+}$ (Excitation of reference and sample compounds was performed at the same wavelength).

Electronic circular dichroism spectra (ECD, in M^{-1} cm^{-1}) were recorded on a Jasco J-1700 Circular Dichroism Spectrometer. The circularly polarized luminescence (CPL) measurements were performed using a JASCO 300 CPL spectrofluoropolarimeter. The following parameters were used: emission slit width = 4 mm, integration time = 4 sec, scan speed = 50 nm/min. Concentration of compounds was adjusted to reach optical density not larger than ~0.1 at the excitation wavelength (here λ_{exc}= 320 nm) to avoid self-reabsorption and also photoselection. The reported CPL spectra are resulting from the average of accumulations of 10 scans.

Electrochemical tests are conducted in a three-electrode system. The working electrode is a platinium electrode, the reference electrode is Ag/AgCl, and the counter electrode is platinum wire. Cyclic voltammetry studies are performed using the instrument EmStat4S with a scan rate 100 mV/s. The supporting electrolyte was 0.1 N NBu_4PF_6 (tetrabutylammonium hexafluorophosphate) in dichloromethane and solutions were purged with argon before the measurements.

The crystals of **6** and **7** were grown by slow evaporation of a CH_2Cl_2. The structure was determined by standard crystallographic methods. These datas can be obtained on the Cambridge Crystallographic Data Centre via www.ccdc.cam.ac.uk/data_request/cif), the CCDC number is 2566568 for **6** and 2566609 for **7** respectively.

b) Theory

We have performed the DFT and TD-DFT calculations with Gaussian 16.[1] No structural simplification was performed. Point-group symmetry was used when suited. Default Gaussian16 thresholds and algorithms were used but for an improved optimization threshold (10^{-5} au on average residual forces), a stricter self-consistent field convergence criterion (10^{-10} a.u. for optimization/frequencies, 10^{-8} a.u. for single-point calculations) and the use of the *superfine* DFT integration grid, the largest pruned grid available in Gaussian.

Firstly, the S0 geometries have been optimized with DFT and the vibrational frequencies have been analytically determined, using the PBE0 hybrid exchange-correlation functional.[2] This functional works

especially well for thiocarbonyl derivatives.[3] These calculations were performed with the 6-31+G(d) atomic basis set and account for solvent effects through the linear-response PCM approach considering $CHCl_3$ as solvent.[4] We selected this solvent, since this aprotic solvent is typically the one the best modeled by the PCM approach. These ground-state optimizations have been carried out starting with a series of chemically-sound conformers and the following steps were performed on the most stable one (according to DFT computed free energies). The structures are true minima of the PES, except for the transition states of rotation that presents one single imaginary mode corresponding to the expected torsion. Secondly, starting from the optimal ground-state geometries, we have used TD-DFT with the same functional and basis set to optimize the S_1 and S_2 geometries and compute their vibrational frequencies. All optimized structures correspond to true minima of the potential energy surface, except obviously for the rotation transition state. Thirdly, the vertical transition energies were determined with TD-DFT and the same functional, but a larger basis set, namely 6-311++G(d,p), in gas-phase as well as in solution using the cLR^2 variant of the PCM,[5] in its non-equilibrium limit. The lowest triplet state (T_1) structures have been optimized using a consistent level of theory within the unrestricted DFT (U-DFT) formalism.

As we are aware of the significant dependency of the TD-DFT results on the selected functional,[6] the obtained transition energies were also computed using CC2[7] with the Turbomole 7.3 code.[8] The CC2 energies were calculated in gas phase applying the resolution of identity scheme and the frozen-core approximations, and using the *aug*-cc-pVTZ atomic basis set. Combining the CC2 and TD-DFT data using a well-known protocol,[9] one can obtain accurate CC2-corrected estimates of the absorption, fluorescence, and 0-0 energies that can be straightforwardly compared to experimental values. The 0-0 phosphorescence energies have been determined with the same protocol, but using the SCS-CC2 rather than CC2 energies, since SCS-CC2 provides more accurate relative energies for singlet-triplet cases.[10,11]

The spin-orbit coupling elements were computed using the same PBE0 hybrid functional, the ZORA Hamiltonian and the ma-*def2*-TVPP basis set a for the calculations using ORCA 6.[12] TDA was not applied, RI was used, and the so-called “1,4,4,1” options were selected for estimating the SOC. Solvent effects were included through the SMD model, with $CHCl_3$ as solvent. The reported SOC values reported in the text have been computed as:

$$\sqrt{\frac{1}{3}S_x^2 + \frac{1}{3}S_y^2 + \frac{1}{3}S_z^2}$$

The S-T gaps determined when studying the ISC process have been computed with the same methodology (and ORCA), as well as at SCS-CC2/aug-cc-pVTZ level in gas-phase (with Turbomole). SCS-CC2[10,11] is known to provide accurate S-T gaps. We also report the PBE0 gaps given by ORCA. For the records, the latter are highly similar to the PCM-PBE0/6-311+G(2d,p) obtained with Gaussian.

All the topological analyses were carried out using the TopChem2 program, version 5.01.[13,14]

[1] M. J. Frisch, et al. Gaussian 16, revision A.03; Gaussian Inc.: Wallingford, CT, 2016.
[2] a) C. Adamo, V. Barone, J. Chem. Phys., 1999, 110, 6158−6170 ; b) M. Ernzerhof, G. E. Scuseria, J. Chem. Phys., 1999, 110, 5029−5036.
[3] D. Jacquemin, V. Wathelet, E. A. Perpète, J. Phys. Chem. A., 2006, 110, 9145−9152.
[4] J. Tomasi, B. Mennucci, R. Cammi, Chem. Rev., 2005, 105, 2999−3094.
[5] C. A. Guido, A. Chrayteh, G. Sclamani, B. Mennucci, D. Jacquemin J. Chem. Theory Comput., 2021, 17, 5155−5164.
[6] A. D. Laurent, D. Jacquemin, Int. J. Quantum Chem., 2013, 113, 2019−2039.
[7] O. Christiansen, H. Koch, P. Jørgensen Chem. Phys. Lett., 1995, 243, 409−418.
[8] TURBOMOLE V7.3/V7.5/V7.8, a development of University of Karlsruhe and Forschungszentrum Karlsruhe GmbH, 1989−2007; TURBOMOLE GmbH. http://www.turbomole.com.
[9] D. Jacquemin, I. Duchemin, X. Blase, J. Chem. Theory Comput., 2015, 11, 5340−5359.
[10] A. Pershin, D. Hall, V. Lemaur, J. C. Sanchi-Garcia, L. Muccioli, E. Zysman-Colman, D. Beljonne, Y. Olivier, Nat. Commun., 2019, 10, 597.
[11] P. F. Loos and D. Jacquemin, J. Comput. Chem., 2024, 45, 1179−1805.

[12] F. Neese, WIREs Comput. Mol. Sci. 2022, 12, e1606.
[13] D. Kozlowski and J. Pilmé, J. Comput. Chem., 2011, 32, 3207–3217.
[14] H. Chevreau and J. Pilmé, J. Comput. Chem., 2023, 44, 1505–1516.

2. Synthesis monomeric and dimeric phthalimide derivatives

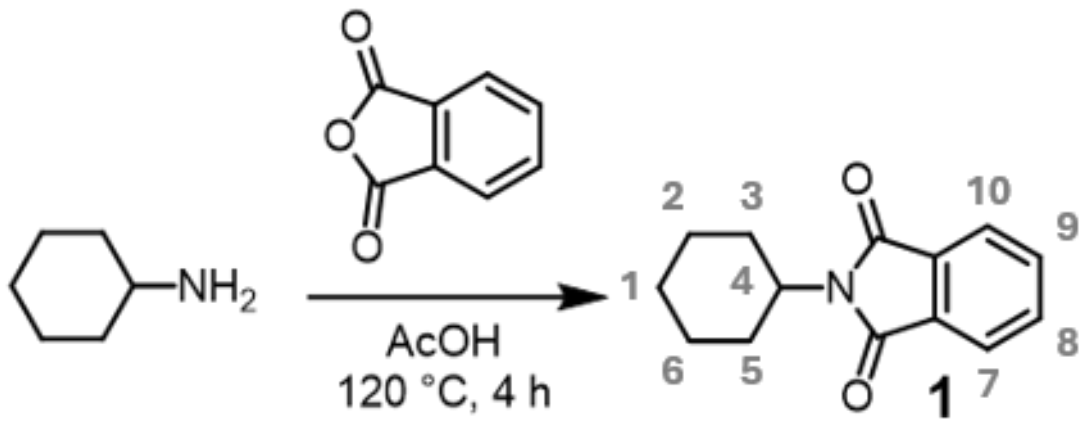

In a Schlenk flask, cyclohexylamine (0.3 mL, 2.52 mmol, 1 equiv.) and phthalic anhydride (0.375 g, 2.52 mmol, 1 equiv.) were dissolved in acetic acid (10 mL). The reaction mixture was stirred at 120 °C during 4h. After cooling to room temperature, distilled water was added, leading to the precipitation of a white solid. The product was collected by vacuum filtration on a fritted funnel, washed with water, and a small volume of ethanol was added to aid in the removal of residual water. After drying under vacuum, compound **1** was obtained as a white powder in 95% yield (0.55 g).

^{1}H NMR (400 MHz, $CDCl_3$): δ = 7.81 (dd, *J* = 5.5, 3.1, 2$H^{10, 7}$), 7.68 (dd, *J* = 5.5 Hz, 3.1, 2$H^{9, 8}$), 4.11 (tt, *J* = 12.5, 3.9, 1H^4), 2.21 (qd, *J* = 12.5, 3.9, 2$H^{5, 3}$), 1.87 (d, *J* = 13.7, 2$H^{5', 3'}$), 1.77 – 1.65 (m, 3$H^{1', 2', 6'}$), 1.34 (m, 3$H^{1, 2, 6}$).
^{13}C NMR (101 MHz, $CDCl_3$) δ = 168.4 (2 C=O), 133.7 (2C), 132.1 (2C), 122.9 (2C), 50.9, 29.8 (2C), 26.0 (2C), 25.1.
HR-MS (ESI-Q-Exactive) calculated for $[C_{14}H_{16}NO_2]^+$ is 230.1175, found 230.1175.

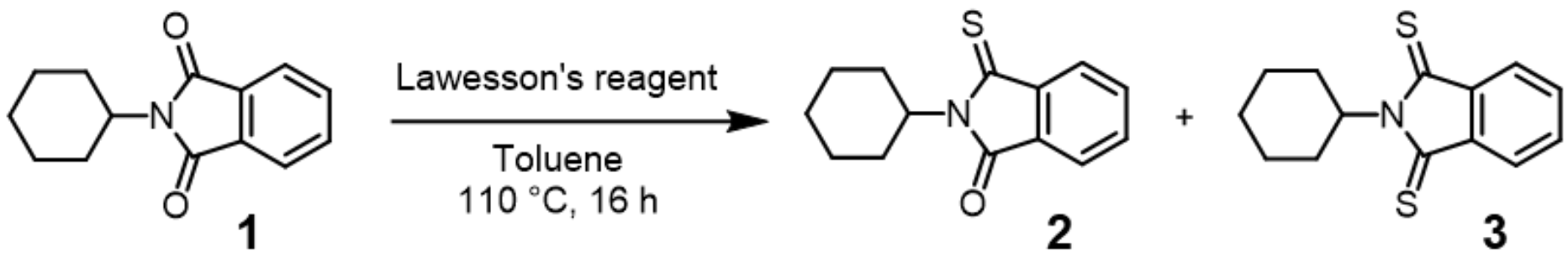

In a two neck round bottom flask equipped with a reflux condenser and under an argon atmosphere, **1** (0.15 g, 0.65 mmol, 1 equiv.) and Lawesson's reagent (0.60 g, 51.47 mmol, 2.25 equiv.) were dissolved in dry toluene (8 mL). The reaction mixture was stirred and refluxed overnight, resulting in an orange solution. After cooling to room temperature, an initial purification was carried out by passing the crude mixture through a short silica plug using CH_2Cl_2 to remove excess Lawesson's reagent and its by-products. The resulting mixture was further purified by column chromatography on silica gel, using CH_2Cl_2/Heptane (2/8) as gradient eluent. Compounds **2 (O,S)** and **3 (S,S)** were isolated from different fractions, with compound **3 (O,S)** eluting first as a brown solid in 10% yield (0.02 g), followed by compound **2 (S,S)** as a green solid in 81% yield (0.13 g).

2 (O,S) :

^{1}H NMR (400 MHz, $CDCl_3$) δ = 7.94 (dd, *J* = 7.6, 3.9 Hz, 1H^7), 7.74 (dd, *J* = 7.6, 3.9 Hz, 1H^{10}), 7.68 (dd, *J* = 7.6, 3.9 Hz, 1$H^{8,9}$), 4.80 (tt, *J* = 12.4, 3.9 Hz, 1H^4), 2.37 (qd, *J* = 12.4, 3.5 Hz, 2$H^{5, 3}$), 1.91 (dt, *J* = 12.4, 3.0 Hz, 2$H^{5', 3'}$), 1.83 – 1.69 (m, 3$H^{1', 2', 6'}$), 1.50 – 1.24 (m, 3$H^{1, 2, 6}$).
^{13}C NMR (101 MHz, $CDCl_3$) δ = 197.4 (C=S), 170.0 (C=O), 136.9, 133.8, 133.0, 127.7, 124.2, 122.4, 54.6, 29.3 (2C), 26.1 (2C), 25.2.
HR-MS (ESI-Q-Exactive) calculated for $[C_{22}H_{19}N_2S_2]^+$ is 262.0718, found 262.0718.

3 (S,S) :

^{1}H NMR (400 MHz, $CDCl_3$): δ = 7.81 (dd, *J* = 5.6, 3.1, 2$H^{7, 10}$), 7.64 (dd, *J* = 5.6, 3.1, 2$H^{8, 9}$), 5.37 (tt, *J* = 12.9, 3.4, 1H^4), 2.82 (qd, *J* = 12.9, 3.7, 2$H^{5, 3}$), 1.90 (d, *J* = 13.3, 2$H^{5', 3'}$), 1.72 (d, *J* = 12.9, 3$H^{1', 2', 6'}$), 1.49 – 1.35 (qt, *J* = 12.9, 3.4, 2$H^{2, 6}$), 1.33 – 1.26 (m, 1H^1).
^{13}C NMR (75 MHz, $CDCl_3$): δ = 191.9 (2 C=S), 140.3, 139.4, 131.1, 128.3, 125.7, 121.7, 55.5, 53.2, 30.8 (2C), 25.5 (2C).
HR-MS (ESI-Q-Exactive) calculated for $[C_{14}H_{16}NOS]^+$ is 246.0947, found 246.0946.

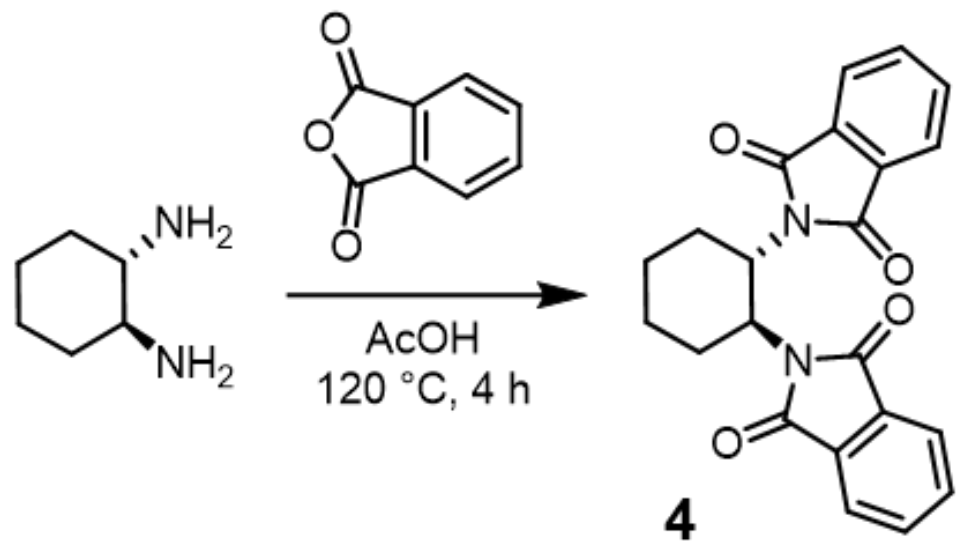

4

In a Schlenk flask, (*R,R*)- or (*S,S*)-trans-1,2-diaminocyclohexane (0.18 mL, 1.53 mmol, 1 equiv.) and phthalic anhydride (0.45 g, 3.07 mmol, 2 equiv.) were dissolved in acetic acid (10 mL). The reaction mixture was stirred at 120 °C during 4h. After cooling to room temperature, distilled water was added, leading to the precipitation of a white solid. The product was collected by vacuum filtration on a fritted funnel, washed with water, and a small volume of ethanol was added to aid in the removal of residual water. After drying under vacuum, compound **4** was obtained as a white powder in 98% yield (0.51 g).

^{1}H NMR (400 MHz, DMSO-d$_6$): δ = 7.95-7.51 (m, 8H$^{7, 8, 9, 10}$), 4.81-4.70 (m, 2H$^{4, 5}$), 2.44-2.26 (m, 2H$^{6', 3'}$), 1.91 (d, *J* = 13.6 Hz, 4H$^{1', 2', 6, 3}$), 1.40 (t, *J* = 9.8 Hz, 2H$^{1, 2}$).
^{13}C NMR (101 MHz, CDCl$_3$) δ = 168.0 (4 C=O), 133.8 (4C), 131.4 (4C), 123.2 (4C), 50.9 (2C), 29.4 (2C), 25.0 (2C).
HR-MS (ESI-Q-Exactive) calculated for [$C_{22}H_{11}N_2O_4Na$]$^+$ is 397.1158, found 397.1161.

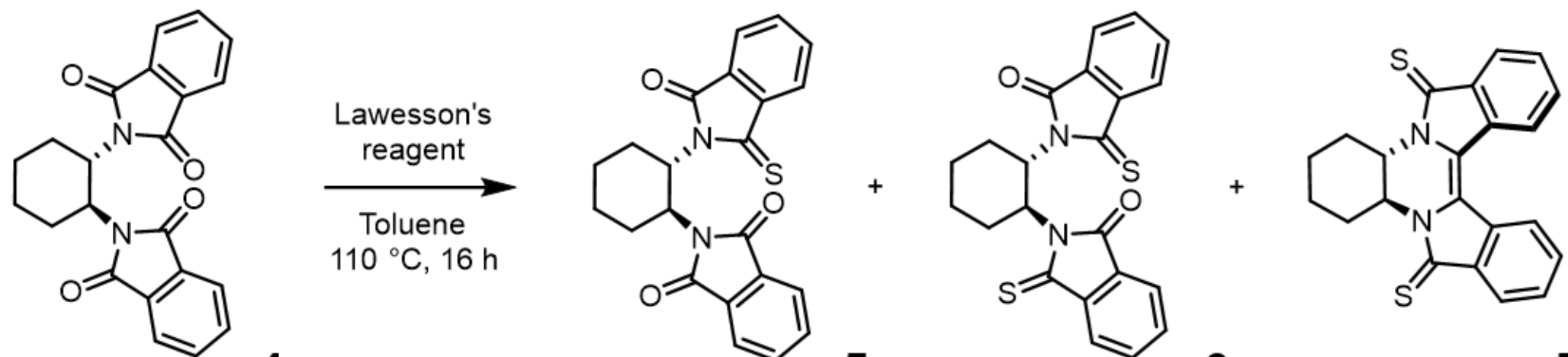

4 **5** **6** **7**

In a two neck round bottom flask equipped with a reflux condenser and under an argon atmosphere, **4** (0.15 g, 0.60 mmol, 1 equiv.) and Lawesson's reagent (0.97 g, 3.61 mmol, 6 equiv.) were dissolved in dry toluene (8 mL). The reaction mixture was stirred and refluxed overnight, resulting in an orange solution. After cooling to room temperature, an initial purification was carried out by passing the crude mixture through a short silica plug using CH_2Cl_2 to remove excess Lawesson's reagent and its by-products. The resulting mixture was further purified by column chromatography on silica gel, The resulting mixture was further purified by column chromatography on silica gel, using CH_2Cl_2/Heptane (2/8) as gradient eluent. Compounds **6 (2S, 2O), 5 (S, 3O)** and **7** were isolated from different fractions, with compound **6 (2S, 2O)** eluting first as a red solid in 34% yield (0.08 g), followed by compound **5 (S, 3O)** as an orange solid in 11% yield (0.03 g) and compound **7** as an ochre solid in 43% yield (0.10 g).

5 (S, 3O) :

^{1}H NMR (400 MHz, CDCl$_3$): δ = 7.75 (dd, *J* = 7.9, 2.6, 2H$^{7a, 10a}$), 7.60 (m, 6H$^{9a, 8a, 10b, 7b, 8b, 9b}$), 5.68 (td, *J* = 11.9, 4.5, 1H^{4}), 5.25 (td, *J* = 11.9, 4.5, 1H^{5}), 2.50 (m, 2H$^{6', 3'}$), 1.95 (m, 4H$^{1', 2', 6, 3}$), 1.59 (m, 2H$^{1, 2}$).
^{13}C NMR (75 MHz, CDCl$_3$): δ = 197.4 (C=S), 170.8 (C=O), 169.5 (2 C=O), 136.5 (2C), 133.7 (2C), 133.1 (2C), 127.6 (2C), 124.3 (2C), 122.8 (2C), 54.8, 50.7, 29.3, 29.1, 25.1, 25.0.
HR-MS (ESI-Q-Exactive) calculated for [$C_{22}H_{19}N_2O_3S$]$^+$ is 391.1110, found 391.1110.

6 (2S, 2O) :

^{1}H NMR (400 MHz, CDCl$_3$): δ = 7.73 (d, *J* = 6.8, 2H$^{10a, 7a}$), 7.69 (d, *J* = 6.5, 2H$^{10b, 7b}$), 7.63 – 7.52 (m, 4H$^{9a, 8a, 9b, 8b}$), 5.91 (m, 2H$^{4, 5}$), 2.56 (m, 2H$^{6', 3'}$), 1.97 (m, 4H$^{1', 2', 6, 3}$), 1.62 (m, 2H$^{1, 2}$).
^{13}C NMR (75 MHz, CDCl$_3$): δ = 197.4 (2 C=S), 169.5 (2 C=O), 136.5 (2C), 133.7 (2C), 133.1 (2C), 127.6 (2C), 124.3 (2C), 122.7 (2C), 54.3 (2C), 29.0 (2C), 25.0 (2C).

HR-MS (ESI-Q-Exactive) calculated for $[C_{22}H_{19}N_2O_2S_2]^+$ is 407.0882, found 407.0883.

7 :

^{1}H NMR (400 MHz, $CDCl_3$) δ = 8.24 (d, *J* = 7.9 Hz, $2H^{10a, 10b}$), 8.14 (dt, *J* = 7.9, 1.2 Hz, $2H^{7a, 7b}$), 7.65 (td, *J* = 7.9, 1.2 Hz, $2H^{8a, 8b}$), 7.55 (m, $2H^{9a, 9b}$), 4.85 (dt, *J* = 13.5, 2.5 Hz, $2H^{4, 5}$), 4.41 – 4.29 (m, $2H^{6', 3'}$), 1.97 (td, *J* = 5.5, 2.5 Hz, $2H^{1', 2'}$), 1.73 (m, $2H^{6, 3}$), 1.61 (m, $2H^{1, 2}$).
^{13}C NMR (101 MHz, $CDCl_3$) δ = 188.2 (2 C=S), 139.2 (2C), 131.3 (2C), 131.0 (2C), 129.6 (2C), 126.2 (2C), 125.6 (2C), 121.7 (2C), 62.6 (2C), 29.3 (2C), 24.4 (2C).
HR-MS (ESI-Q-Exactive) calculated for $[C_{22}H_{19}N_2S_2]^+$ is 375.0984, found 375.0982.

3. Crystallographic structures

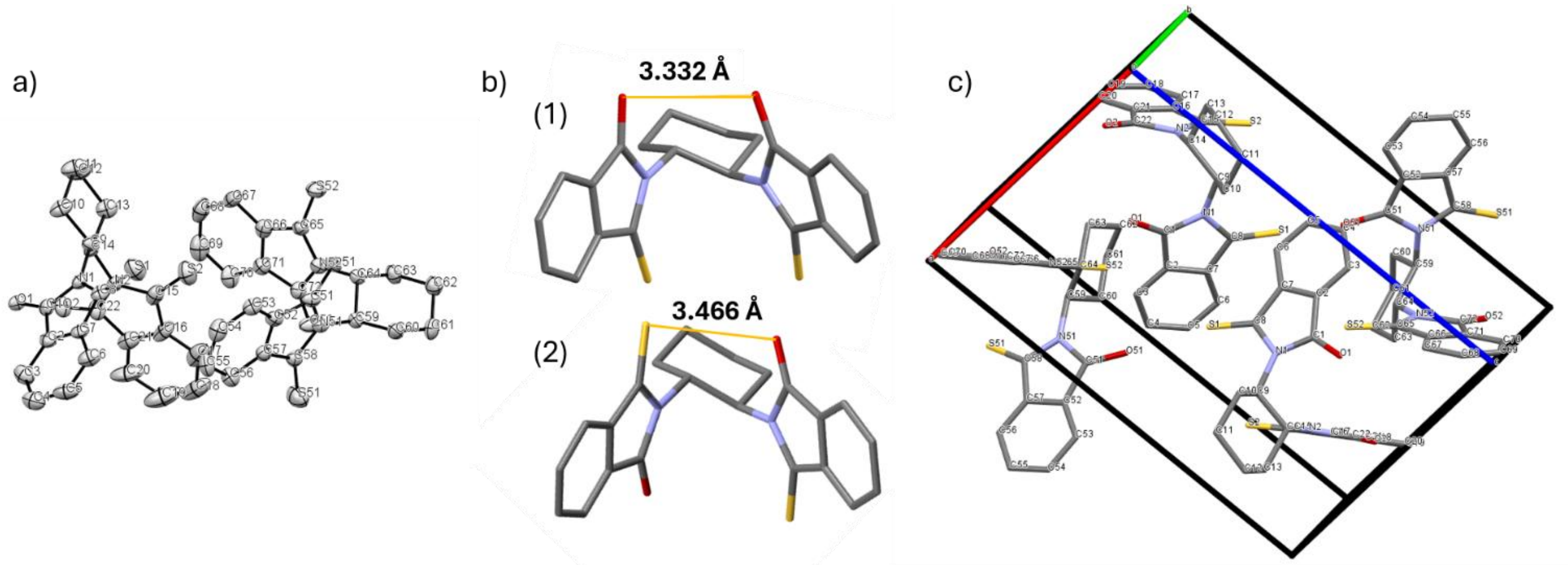

Figure S1: *Single crystal X-ray crystallography of a) ORTEP structure of (**6**) b) Face-to-face dimer unit (**6**) and head-to-tail dimer unit (**6**) c) packing of the compound in the unit cell.*

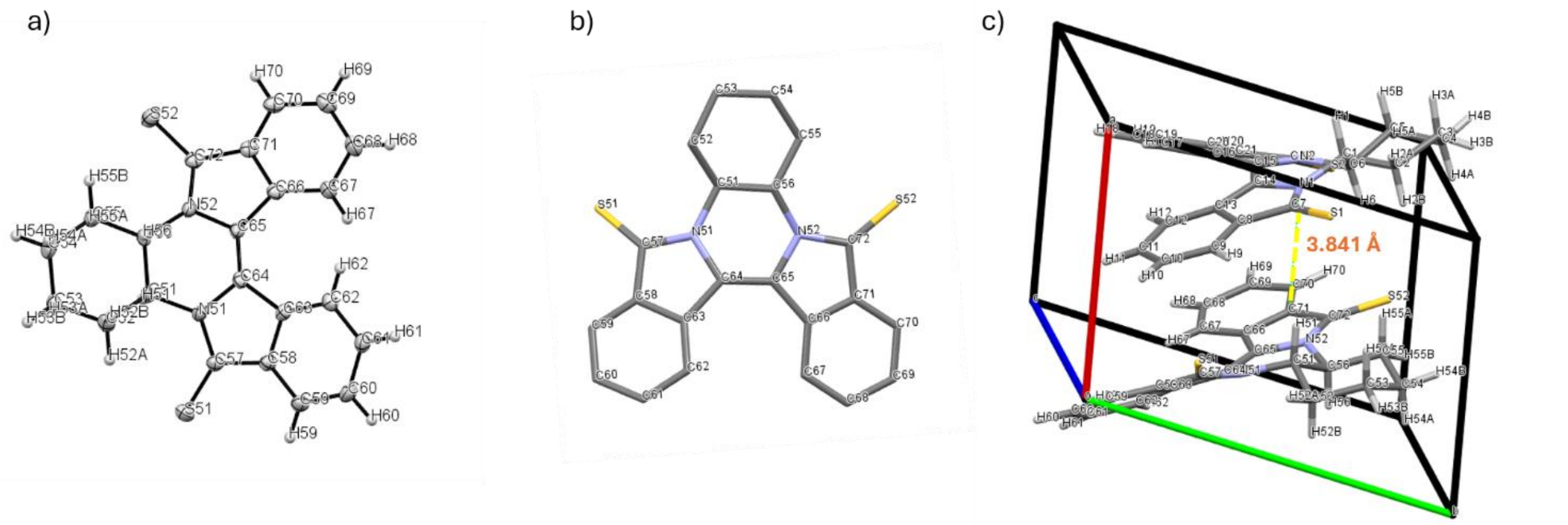

Figure S2: *Single crystal X-ray crystallography of a) ORTEP structure of **7**, b) Monomer unit **7**, c) packing of the compound in the unit cell.*

Table S1: *Crystallographic parameters for **6** and **7**.*

Product	**6 (2O, 2S)**	**7**
Formula	‘$C_{22}H_{18}N_2O_2S_2$’	‘$C_{22}H_{18}N_2S_2$’
FW (g/mol)	406.50	374.50
Crystal system	Monoclinic	Triclinic

Space group	P $_{21}$	P $_{1}$
a, Å	9.2130(7)	7.8096(5)
b, Å	13.9310(10)	10.1668(6)
c, Å	15.2001(11)	11.8257(6)
α, °	90	93.661(2)
β, °	97.620(3)	99.687(2)
γ, °	90	104.076(2)
V, Å^3	1933.6(2)	892.36(9)
Z	4	2
T, K	150(2)	150(2)
Absorption coefficient μ, mm^{-1}	0.296	0.306
Refls. coll.	14864	16601
Ind. Refls. (R_{int})	8221(0.0615)	7534(0.0576)
R_1 (I>2σ(I))[a]	0.0745	0.0453
wR_2 (I>2σ(I))[a]	0.1848	0.1081
R_1 (all data)[a]	0.1045	0.0575
wR_2 (all data)[a]	0.2050	0.1146
GOF	1.053	1.016

4. Rotamers in solution

From theoretical data obtained through DFT calculations and experimental NMR measurements, it is possible to estimate thermodynamic parameters associated with conformational equilibria in solution. At room temperature, the integration of proton signals in the NMR spectrum provides the relative populations of two conformers coexisting in solution. These populations can be related to the equilibrium constant between conformers. Using this equilibrium constant and the **Arrhenius** or **Eyring** (Figure **S3**) equation, one can access the free energy difference (**ΔG°**) between the conformers, as well as the activation parameters (**ΔG***) associated with their interconversion.

$$\frac{N_{\bullet}}{N_{\blacktriangle}} = e^{\left(\frac{-\Delta G^{\circ}}{RT}\right)}$$

N = Population of conformers (obtained from NMR)
ΔG° = Gibbs free energy
R = Gas constant
T = Temperature in Kelvin (K)

$$\Delta G^{*} = RT\,ln\left(\frac{k_B T c}{h\,k}\right)$$

R = Gas constant
T = Temperature (K)
ΔG* = Energy of activation
$\mathbf{K_B}$ = Boltzmann constant
$\mathbf{T_C}$ = Temperature of signal coalescence (K)
h = Planck's constant
k = Rate constant

Figure S3: *Thermodynamic relationships used to describe the equilibrium between the two conformers in solution and the energy required for their interconversion.*

a) Gibbs free energy

In this way, the combination of DFT-computed energies and experimentally determined proton integrations offers a consistent approach to evaluate conformational stability and to derive **ΔG°** values in solution.

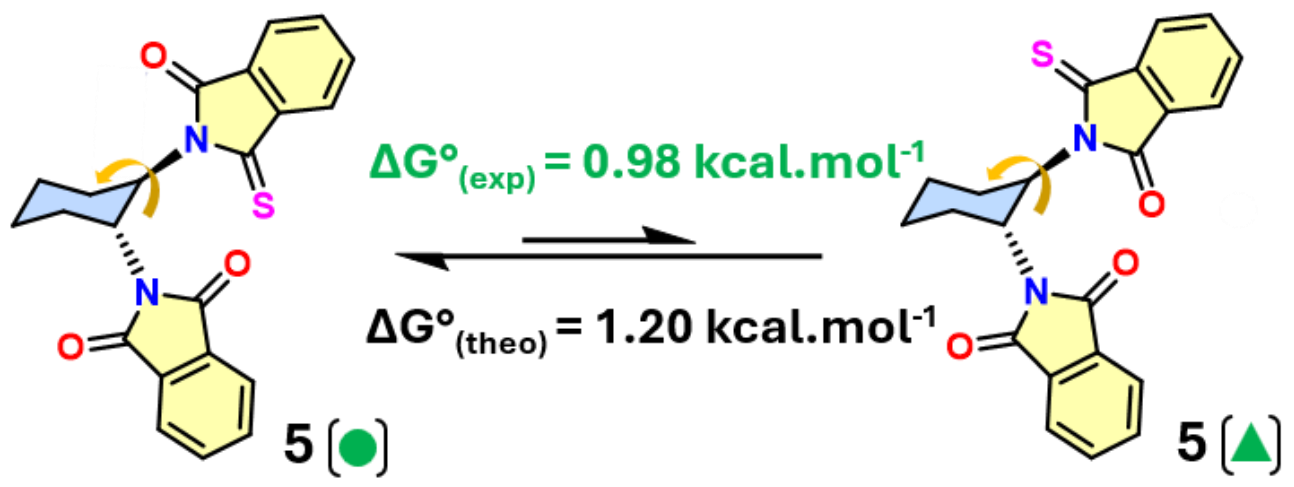

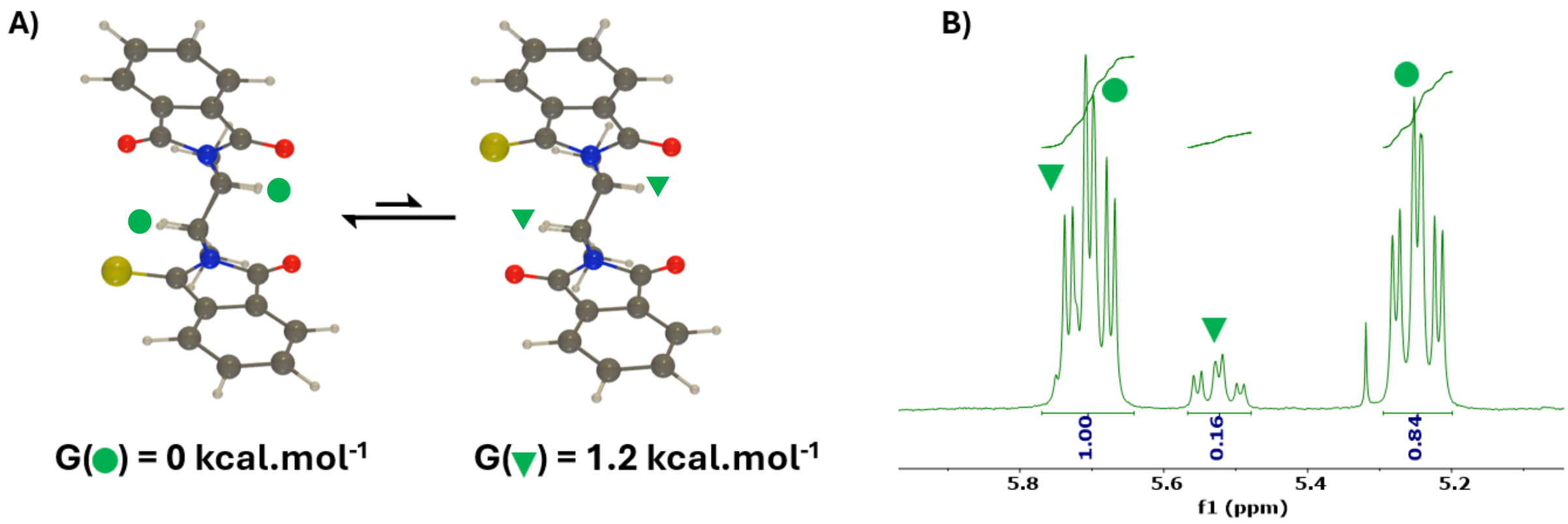

Figure S4: *A) Representation of the two conformers of **5** together with their relative free energies (G) in kcal.mol^{-1}, B) ^{1}H NMR of **5** at 298 K in $CDCl_3$ reveals two sets of signals corresponding to two conformers in solution at room temperature.*

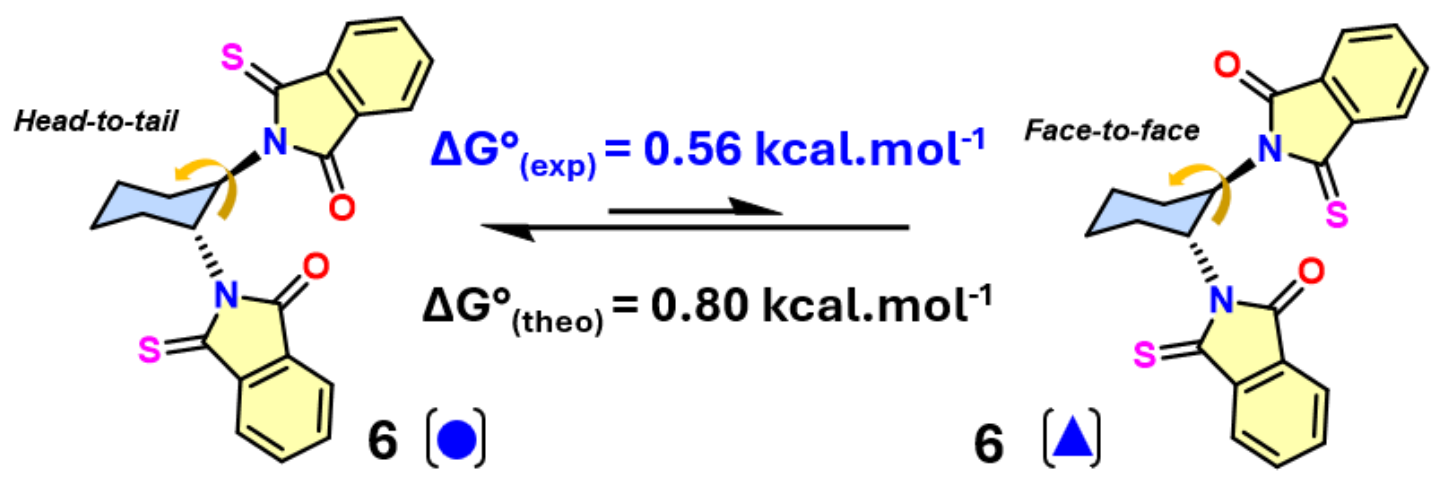

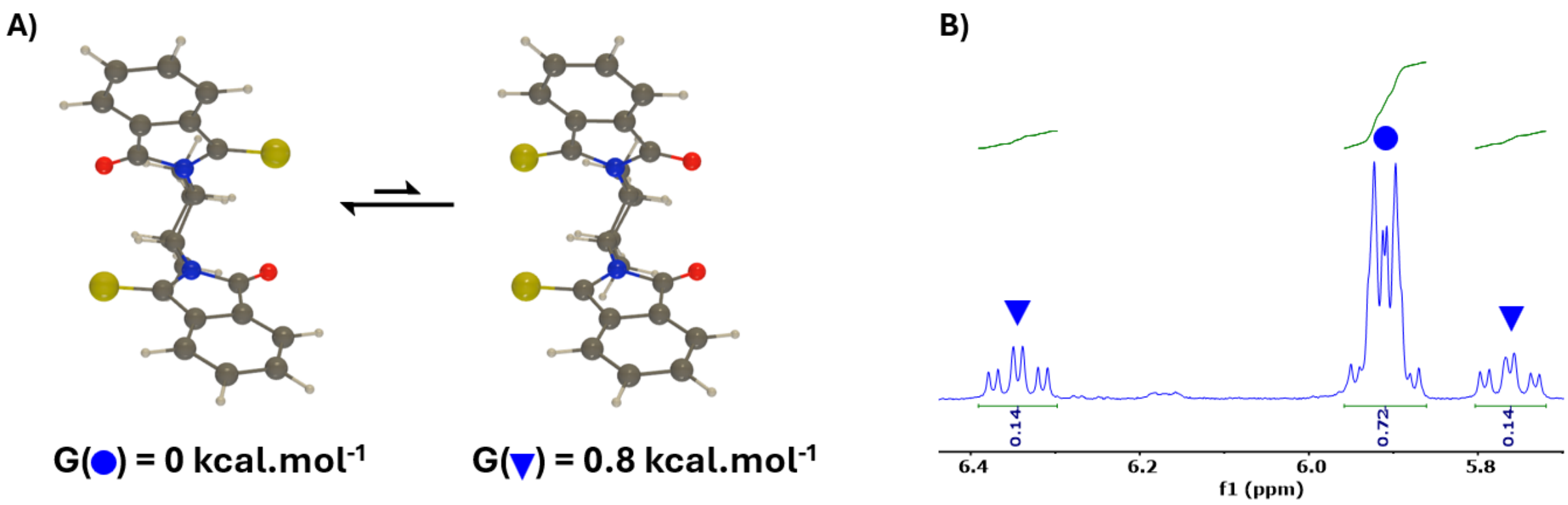

Figure S5: *A) Representation of the two conformers of **6** together with their relative free energies (G) in kcal.mol^{-1}, B) ^{1}H NMR of **6** at 298 K in $CDCl_3$ reveals two sets of signals corresponding to two conformers in solution at room temperature.*

b) Energy of activation

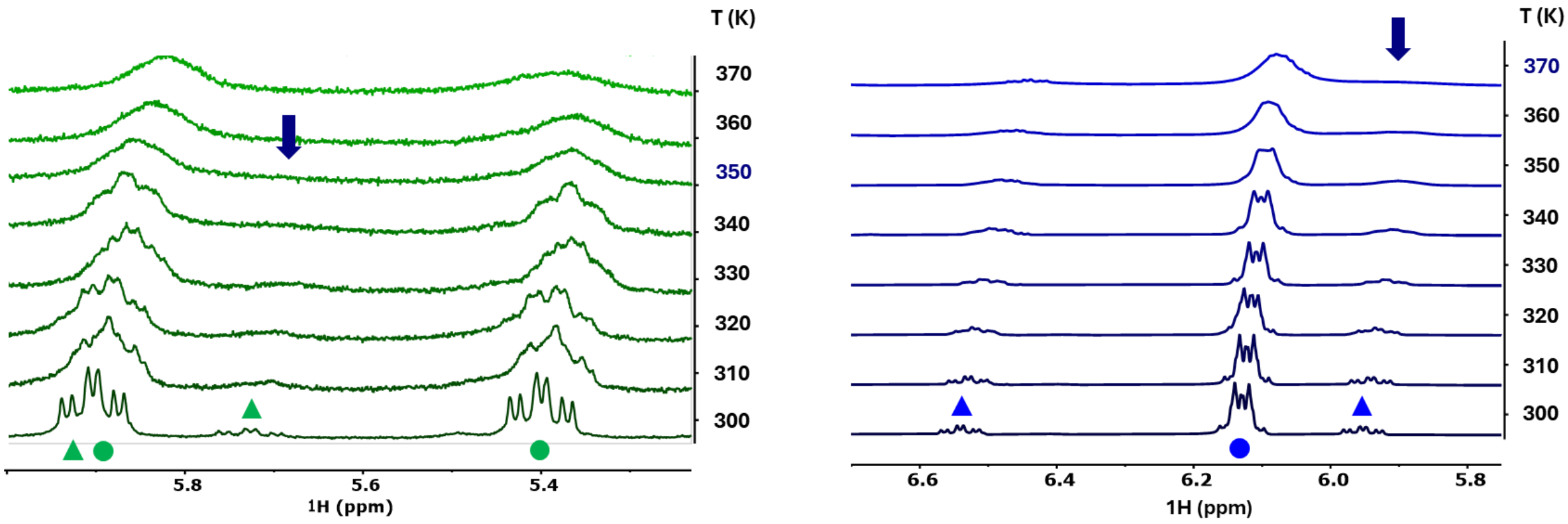

Figure S6: *VT-¹H-NMR of **5** (left) and **6** (right) of the two conformers between 300-370 K (400 MHz, Toluene-d_6).*

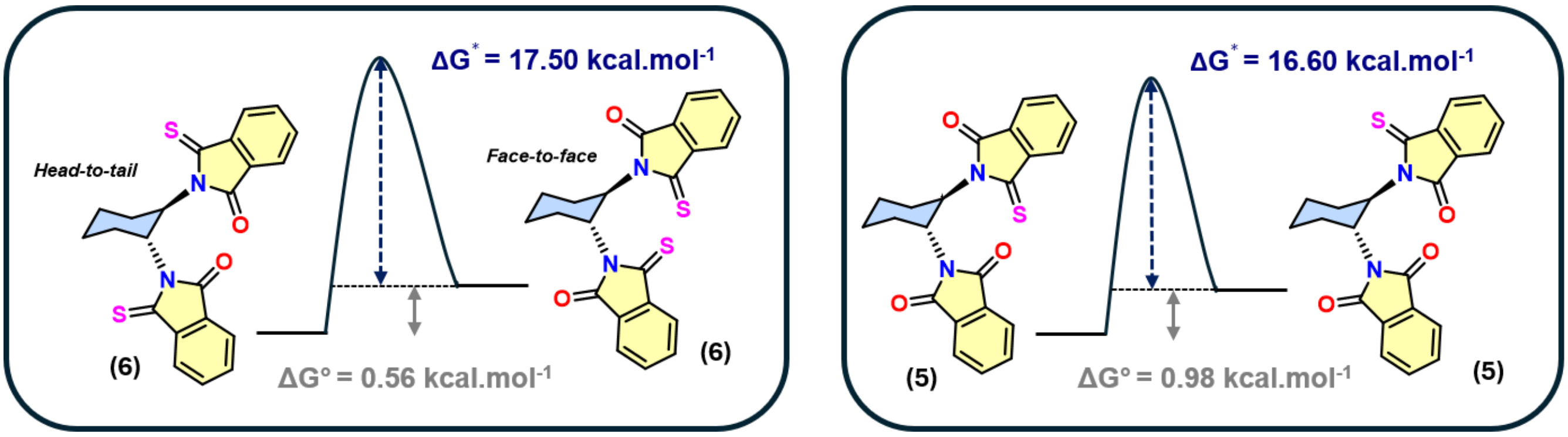

At the coalescence temperature, the rate constant is related to the chemical shift difference (in Hz) by:

$$\boldsymbol{k} = \frac{\boldsymbol{\pi\Delta\nu}}{\sqrt{\mathbf{2}}}$$

where $\boldsymbol{\Delta\nu}$ is the frequency separation (Hz) between the two proton signals in the slow-exchange regime ($T = 300$ K). For measurements performed on a 400 MHz spectrometer, $\boldsymbol{\Delta\nu}$ was obtained from the chemical-shift difference using $\boldsymbol{\Delta\nu} = \mathbf{400} \times \boldsymbol{\Delta\delta}$, where $\boldsymbol{\Delta\delta}$ is expressed in ppm. The chemical shift differences between the two forms in this regime are **Δδ** = 0.354 ppm and **Δδ** = 0.403 ppm for compounds **5** and **6**, respectively. The coalescence temperatures of the signals between the two rotamers are on the order of $\mathbf{T_C}$ = 350 K for the phthalimide derivative **5** and for its analogue **6**, a temperature of $\mathbf{T_C}$ = 370 K is taken (even if the coalescence of the peaks is not totally observed, limited by the boiling point of the used solvent), which leads to activation energy values for the interconversion of the rotamers of **ΔG*** = 16.60 kcal·mol⁻¹ and **ΔG*** = 17.50 kcal·mol⁻¹ for **5** and **6**, respectively.

5. Photophysical properties

a) Absorbance & luminescence spectra

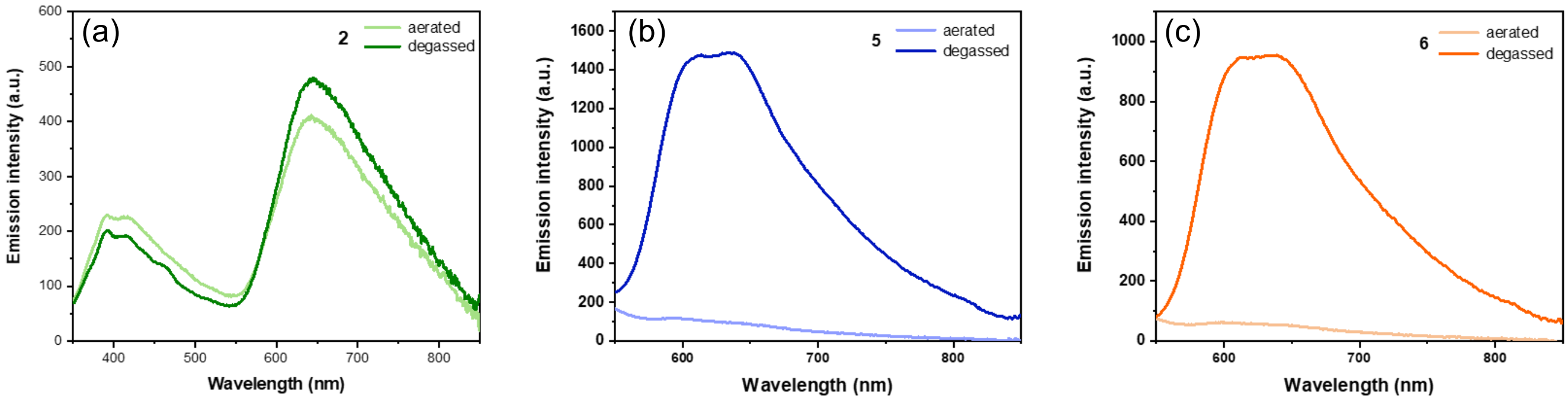

Figure S7: *Effect of oxygen on emission spectra of (a)* ***2****, (b)* ***5****, and (c)* ***6*** *in $CHCl_3$ (5 x 10^{-5} M) at 298 K under aerated (light lines) and degassed (dark lines) conditions, recorded upon excitation at λ_{exc}= 300 nm.*

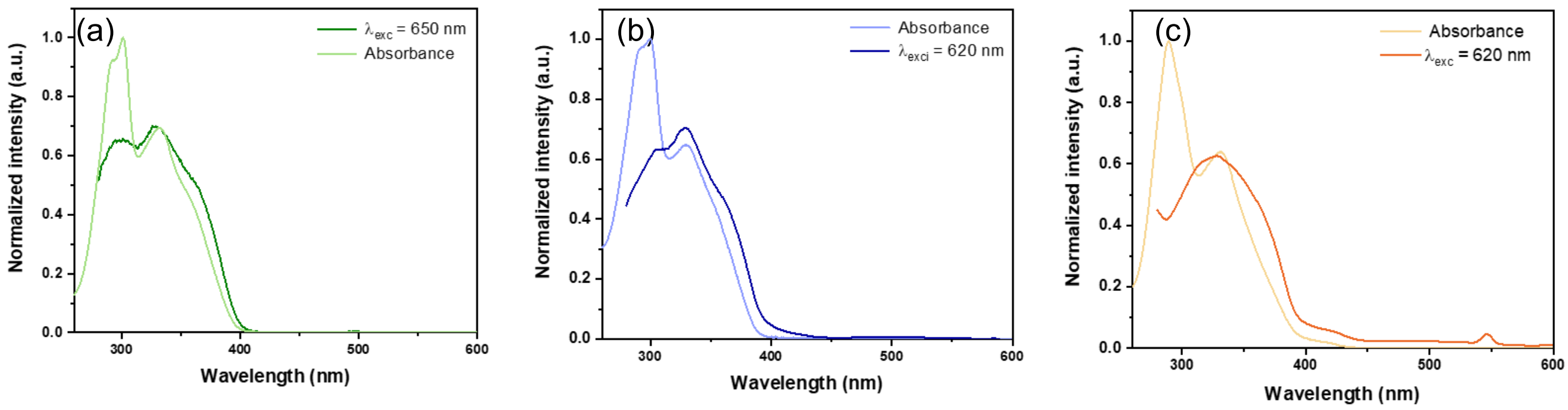

Figure S8: *Excitation spectra of (a)* ***2****, (b)* ***5****, and (c)* ***6*** *in $CHCl_3$ (5 x 10^{-5} M) at 298 K under degassed conditions, recorded at maximum of each emission band (λ_{em}).*

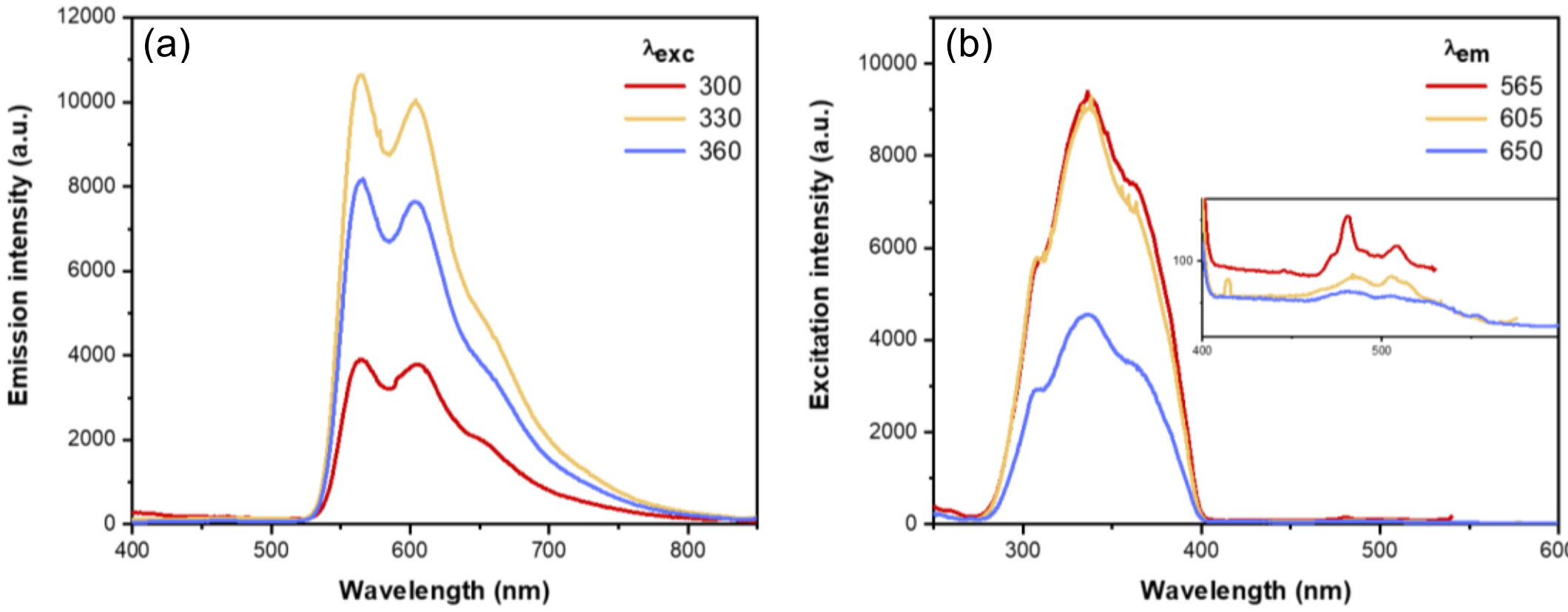

Figure S9: *(a) Emission and (b) excitation spectra of* ***2*** *in $CHCl_3$ (5 x 10^{-5} M) at 77 K, recorded at different excitation and emission wavelengths (λ_{exc} and λ_{em}).*

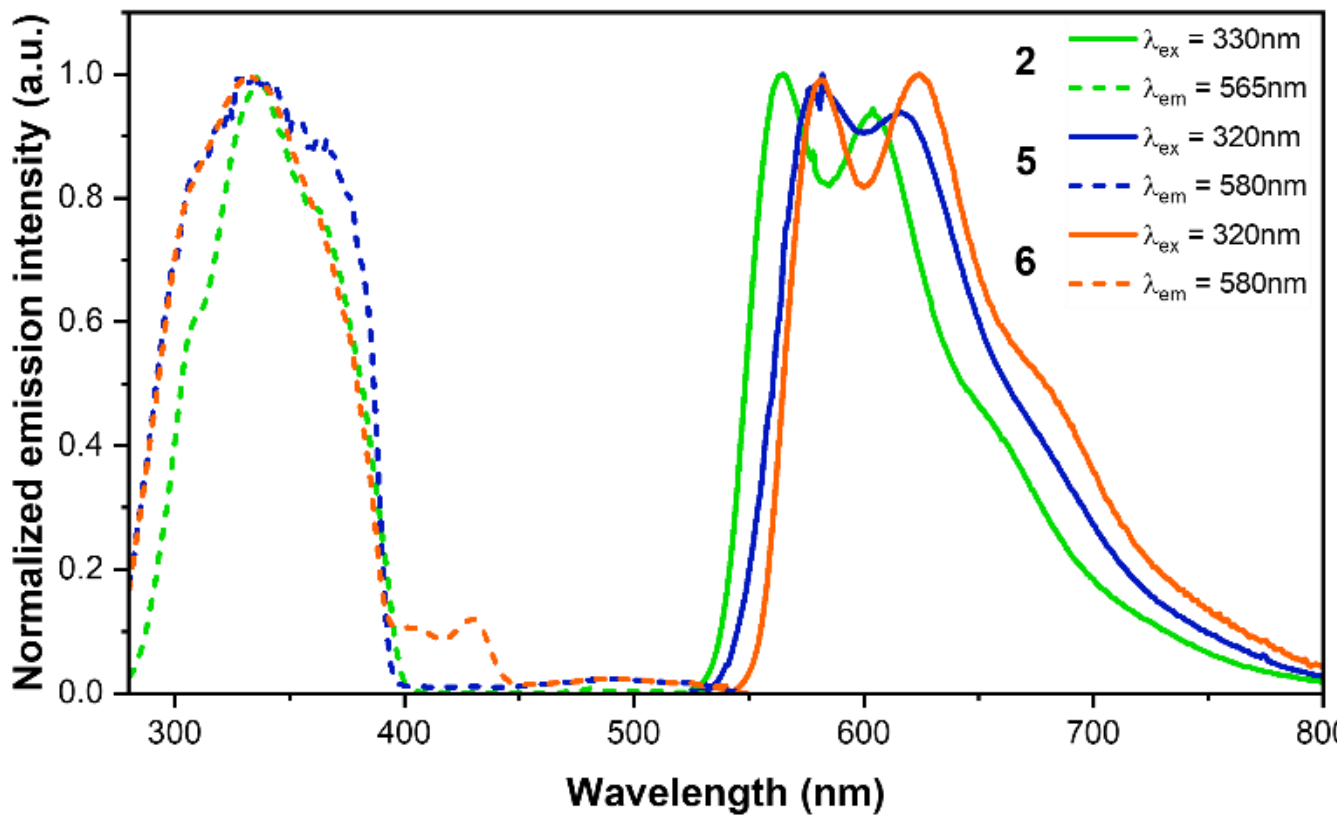

Figure S10: *Normalized excitation (dashed lines) and emission (solid lines) spectra of* ***2****,* ***5*** *and* ***6*** *in $CHCl_3$ (10^{-4} M) at 77 K. Emission spectra were recorded upon excitation at λ_{exc} = 320 or 330 nm, while excitation spectra were monitored at the emission maxima.*

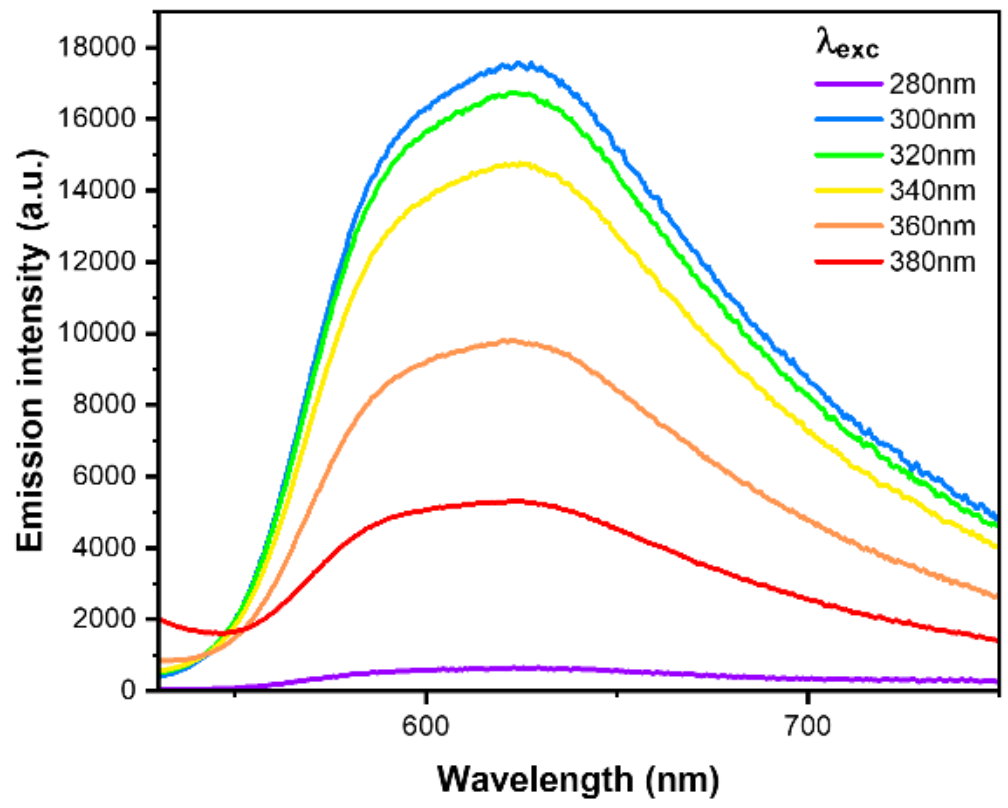

Figure S11: *Excitation-emission map spectra of* ***5*** *in $CHCl_3$ (10^{-4} M) at 298 K under degassed condition.*

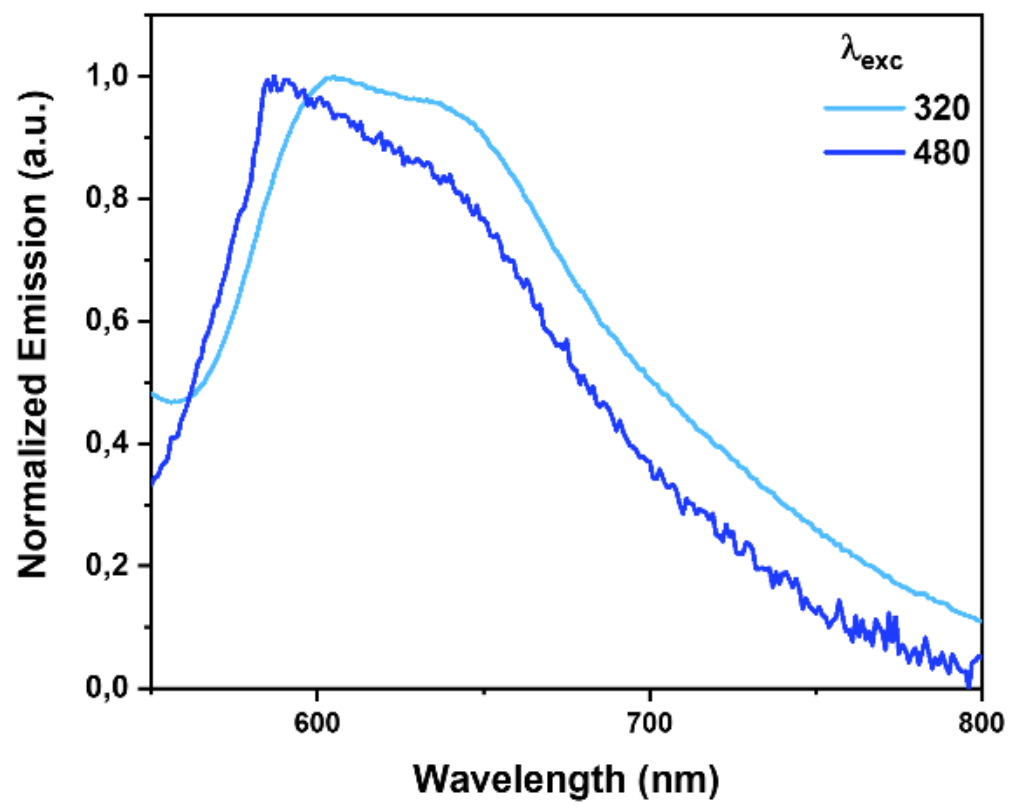

Figure S12: *Emission spectra of* ***5*** *in $CHCl_3$ (10^{-4} M) at 298 K under degassed conditions, recorded at different excitation wavelengths (λ_{exc}).*

b) Phosphorescence lifetime

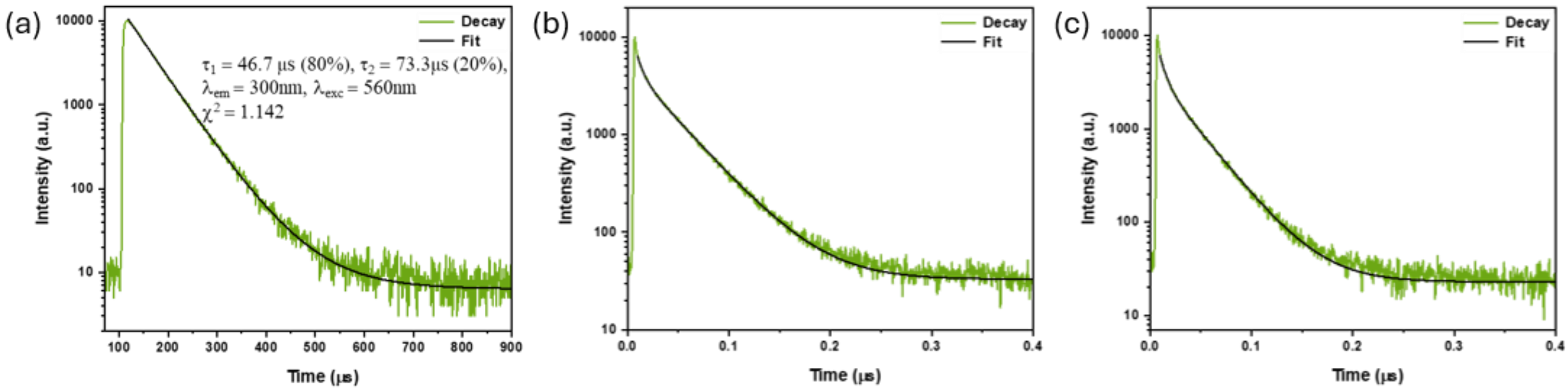

Figure S13: *Phosphorescence lifetime decay profiles of* ***2*** *in $CHCl_3$ (10^{-4} M) recorded at (a) 77 K, (b) 298 K under degassed conditions, and (c) 298 K under aerated conditions.*

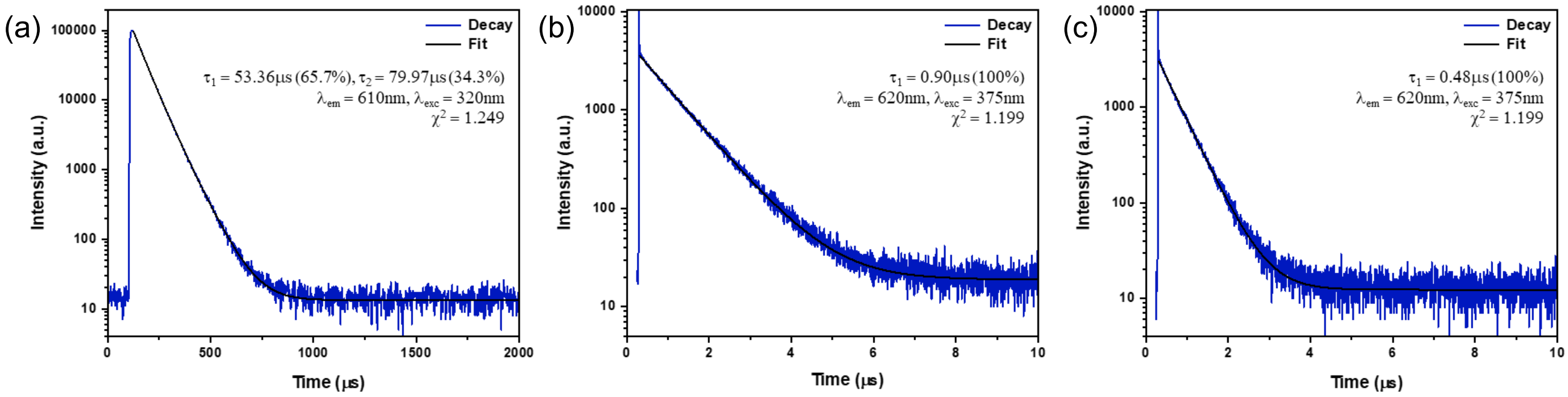

Figure S14: *Phosphorescence lifetime decay profiles of* ***5*** *in $CHCl_3$ (10^{-4} M) recorded at (a) 77 K, (b) 298 K under degassed conditions, and (c) 298 K under aerated conditions.*

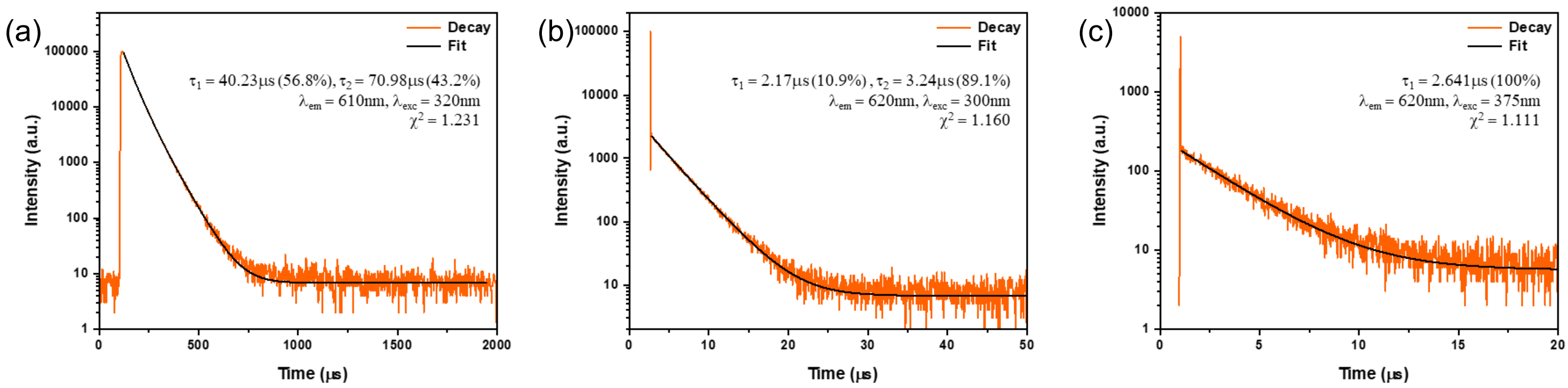

Figure S15: *Phosphorescence lifetime decay profiles of* ***6*** *in $CHCl_3$ (10^{-4} M) recorded at (a) 77 K, (b) 298 K under degassed conditions, and (c) 298 K under aerated conditions.*

We observe a bi-exponential decay at 77 K, with the shorter lifetime being the main component, which deceases in weight when going to compounds **5** and **6**. We indeed relate this fact to the presence of two conformers for all these compounds (see Figure 1 of the manuscript, and Figures. S32, S33, and S34). When we prepared our samples for this measurement, we immersed our quartz tubes in liquid nitrogen, which froze the solution almost instantly, thereby preventing the two conformers from reaching equilibrium. We therefore obtained two different emitting species, with different lifetimes depending on the presence of S---H interaction (for monophthalimide **2**), in addition to the reported S---O interaction (bisphthalimide compounds **5** and **6**).

Table. S2: *Photophysical properties of* ***5*** *and* ***6*** *phosphorescence (in $CHCl_3$ solution at 298K).*

Compound	Φ_P (%)	τ_P (s)	k_P (s^{-1})	k_{nr} (s^{-1})
**6**	1,5	3,12 $\times10^{-6}$	4 838	3,17 $\times10^{5}$
			X2,9	X3,4
**5**	1,3	0,90 $\times10^{-6}$	14 444	1,01 $\times10^{6}$

The values of Φ_P and t_P are generally expressed as:

(1) $\Phi_P(T) = \Phi_{ISC}(T)k_P\tau_P(T)$

(2) $t_P(T) = 1/ k_P + k_{nr}$

The obtained experimental values for **5** and **6** were 1.3 and 1.5 % respectively at 298K (Table S2). Given that the phosphorescence dominates the total PL emission spectra for both **5** and **6**, we consider that $\Phi_{PL} \sim \Phi_P$ and $\Phi_{ISC} \sim 1$ (using $\Phi_{ISC} = 1 - \Phi_F$). Based on the obtained Φ_P and t_P (Table S2), the rate constant k_P and k_{nr} were then derived from (1) and (2) to obtain the desired values (according to the following expression: $k_P = \Phi_P / t_P$ and $k_{nr} = 1 / t_P - k_P$). These values are reported in Table S2. Despite the large k_p estimated for both **5** and **6**, their phosphorescent yields (1.3-1.5%) does not reach the large Φ_P values obtained in some cases (notably in the solid state) certainly, because the magnitude of k_{nr} is also very large for these two compounds in solution. Accordingly, the estimated values of k_{nr} were 1.01×10^6 s^{-1} and 3.17×10^5 s^{-1} for **5** and **6** respectively. Compared to **6**, the magnitude of both k_p and k_{nr} is significantly higher for compound **5**, yet, the luminescence efficiency is comparable for the two compounds because the factor by which k_{nr} and k_p are increased are different (i.e. much larger for k_{nr} (~3.4 vs 2.9). Thus, similar Φ_P values are obtained, despite the additional sulfur atoms of **6**.

c) Photophysical properties of 4

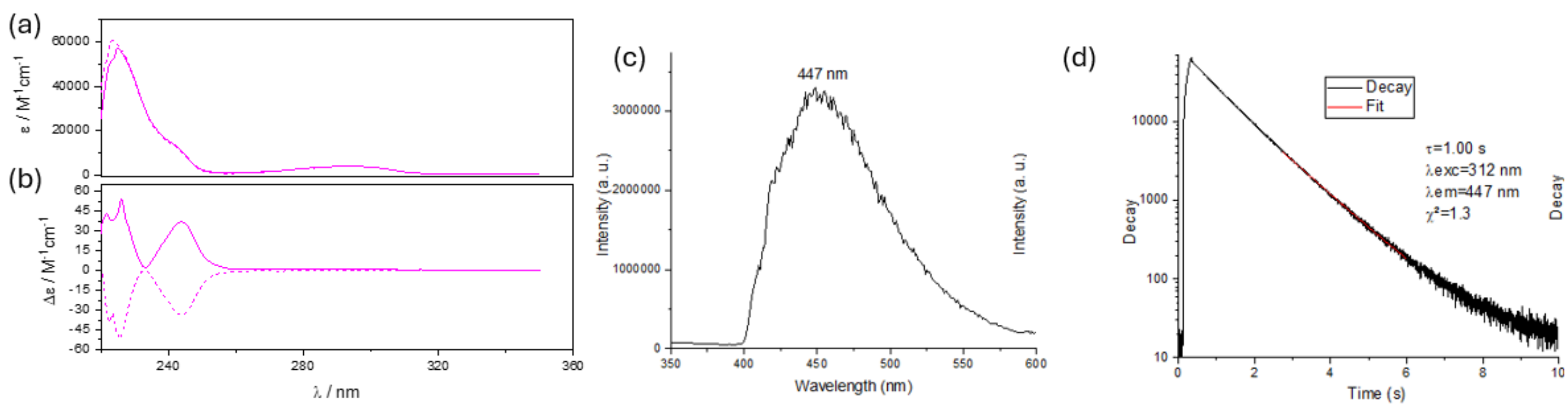

Figure S16: a) Absorption and b) ECD spectra of (+)- and (-)-4 recorded in $CHCl_3$ (~10^{-5} M) at 298 K; c) Photoluminescence spectra of **4** in dilute 2-MeTHF (~10^{-5} M), recorded at 77 K and excited at 310 nm, with d) lifetime decay profile of phosphorescence.

The optical and photophysical properties of chiral oxygenated bisphthalimide compound **4** are reported here to further highlight the effect of the sulfur atom on the optical and photophysical properties of compounds **5** and **6**. Overall, the optical and photophysical properties of compound **4** show a marked blueshifted UV-vis absorption and emission signature compared to both compounds **5** and **6**. The absorption spectrum of **4** shows an intense absorption bands at ca. 230 nm ($\varepsilon \sim 5.7 \times 10^4$ $M^{-1}cm^{-1}$) and smaller broader one at 287 nm ($\varepsilon = 5.0 \times 10^3$ $M^{-1}cm^{-1}$), respectively assigned to π-π^* and n-π^* transitions (these results are in accordance with previously reported ones, see: J. Gawronski, F. Kazmierczak, K. Gawronska, U. Rychlewska, B. Nordén, A. Holmén, J. Am. Chem.

Soc. 1998, 120, 12083). As expected, these transitions appear to be also active in ECD with expected mirror-image spectra for both enantiomers. For instance, compounds (*SS*)- **4** shows positive intense bands at 230 and 242 nm ($\Delta\varepsilon \sim 35\ M^{-1}cm^{-1}$), arising from a chiral excitonic coupling between the electronic dipoles of each phthalimide unit, and an additional very weak transition around 280 nm ($\Delta\varepsilon \sim -0.5\ M^{-1}cm^{-1}$, Figure S1). In solution, compound **4** is poorly emissive ($\phi < 0.01$) owing to an efficient ISC process from the singlet to the triplet excited states of the molecule, as already described for phthalimide derivatives (see for instance: A. Demeter, T. Bérces, L. Biczók, V. Wintgens, P. Valat, J. Kossanyi, J. Chem. Soc., Faraday Trans. 1994, 90, 2635). Lowering the temperature to 77 K in dilute 2-MeTHF afforded exclusively phosphorescence emission. Under 310 nm excitation, compound **4** displayed structured emission band centred at ca. 450-470 nm, arising from π-π* triplet state, associated to a lifetime of *ca.* ~1.0 s. The striking emission differences obtained for compounds **5** and **6** unambiguously show how the incorporation of sulfur atom can help obtaining phosphorescence emission, with shorter lifetimes, at room temperature in fluidic conditions.

6. Cyclic voltammetry

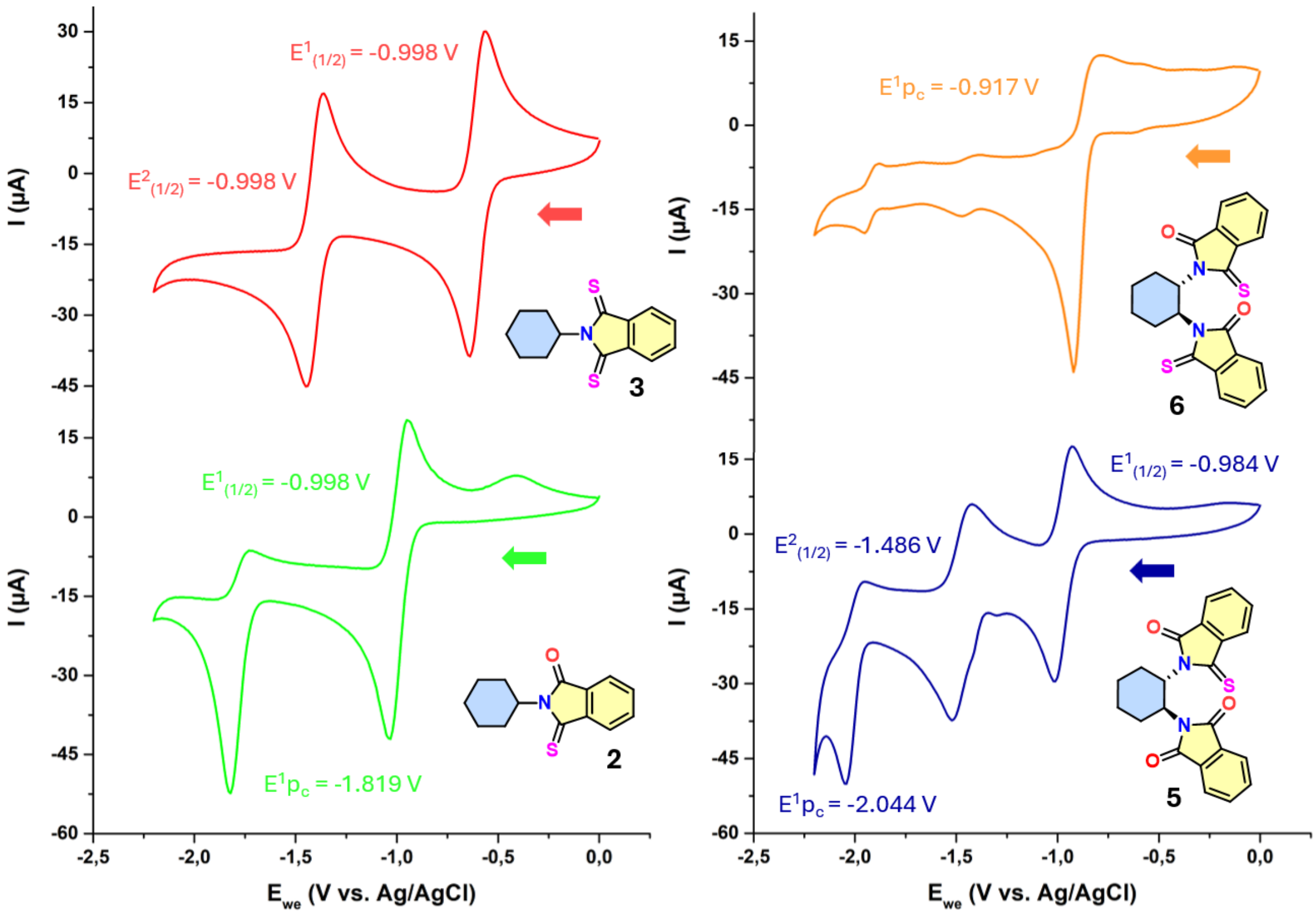

Figure S17: *CV of **3** (red), **6** (navy blue), **5** (orange) and **2** (green) (1 mM) in CH_2Cl_2 with $^nBu_4NPF_6$ (0.1 M) as supporting electrolytes, at a scan rate of 100 mV.s^{-1} on a glassy carbon electrode.*

7. NMR Spectra

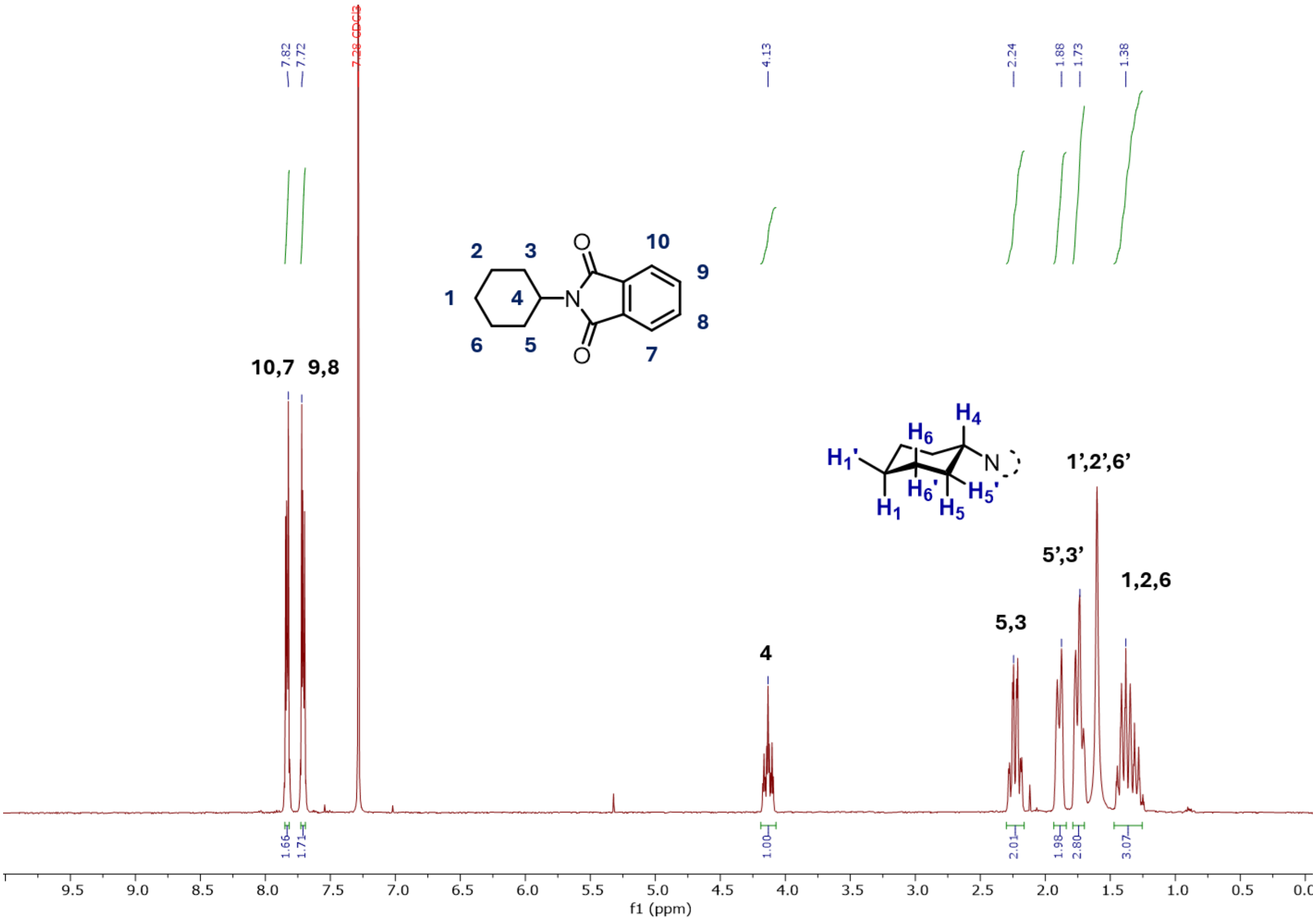

Figure S18: *^{1}H NMR spectra of **1** (CDCl$_3$, 400 MHz).*

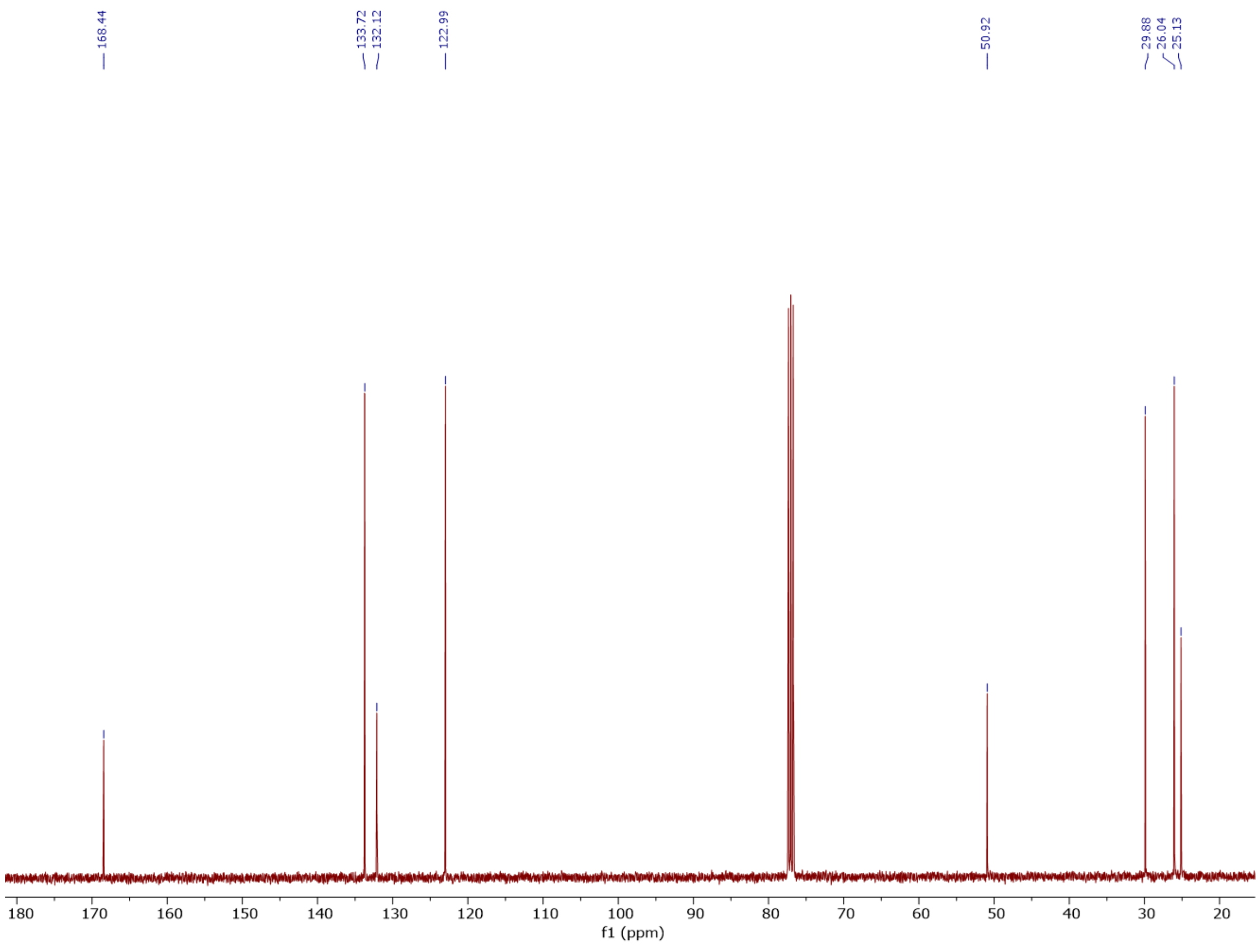

Figure S19: *^{13}C NMR spectra of **1** (CDCl$_3$, 101 MHz).*

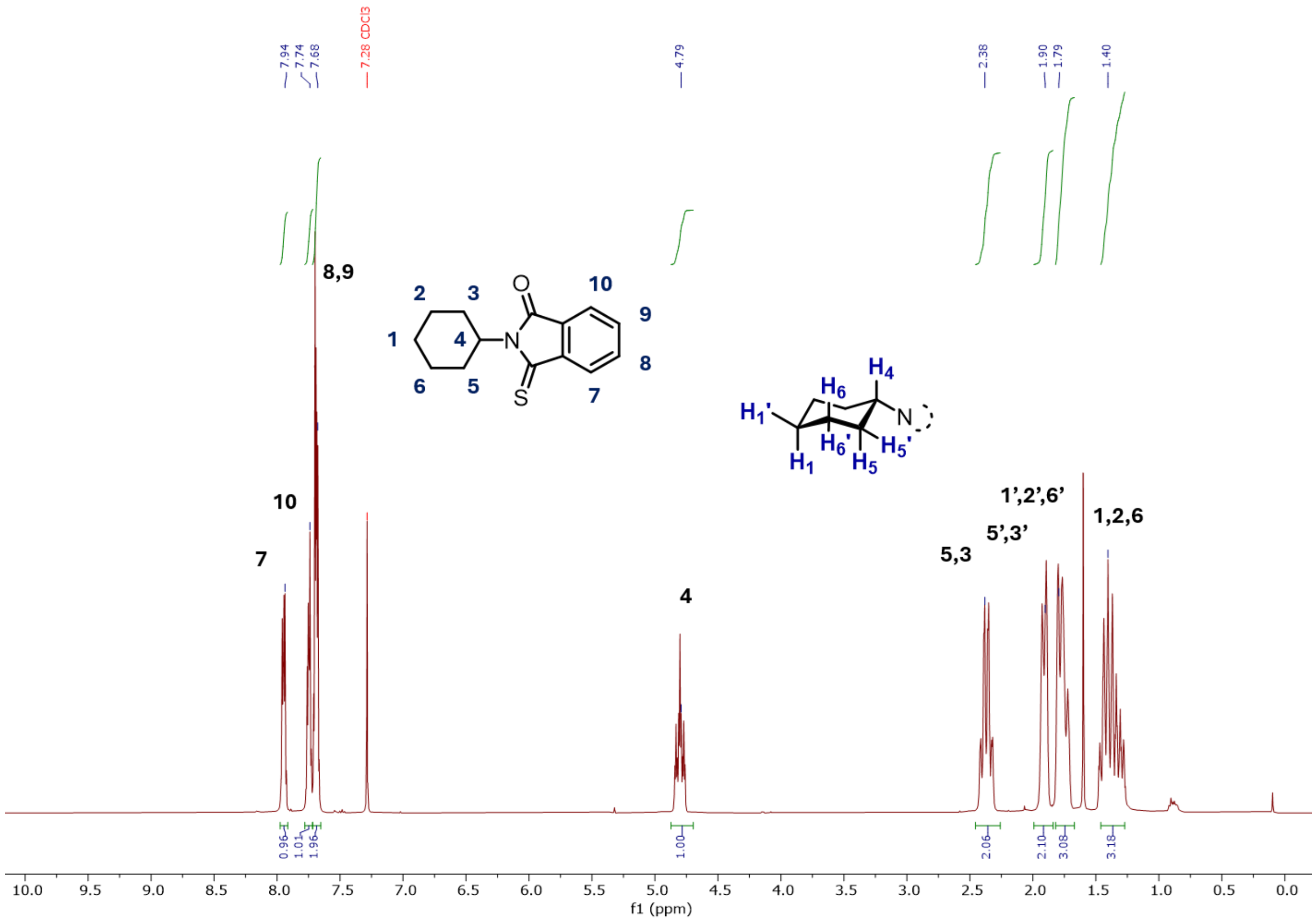

Figure S20: *^{1}H NMR spectra of **2** ($CDCl_3$, 400 MHz).*

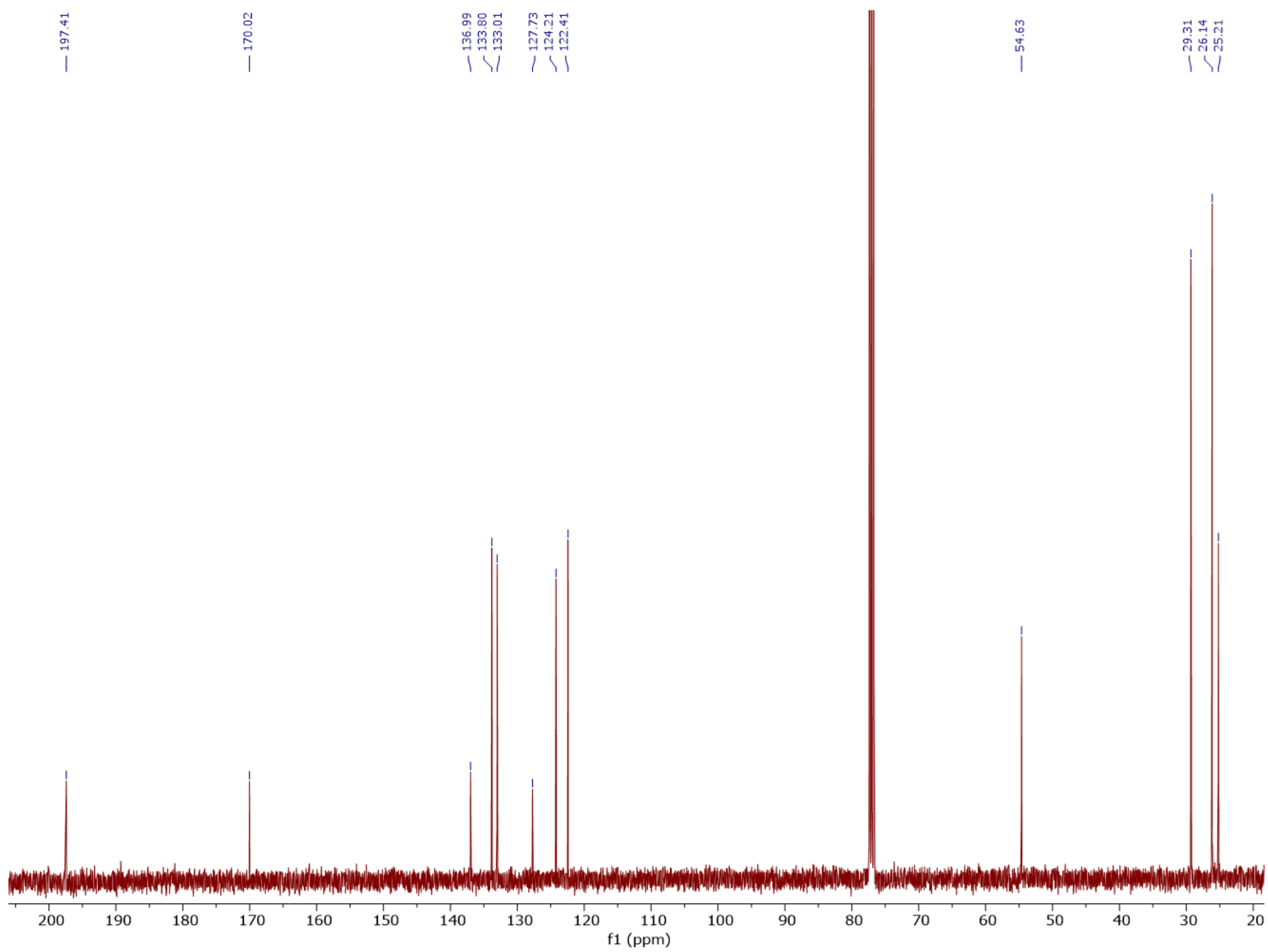

Figure S21: *^{13}C NMR spectra of **2** ($CDCl_3$, 101 MHz).*

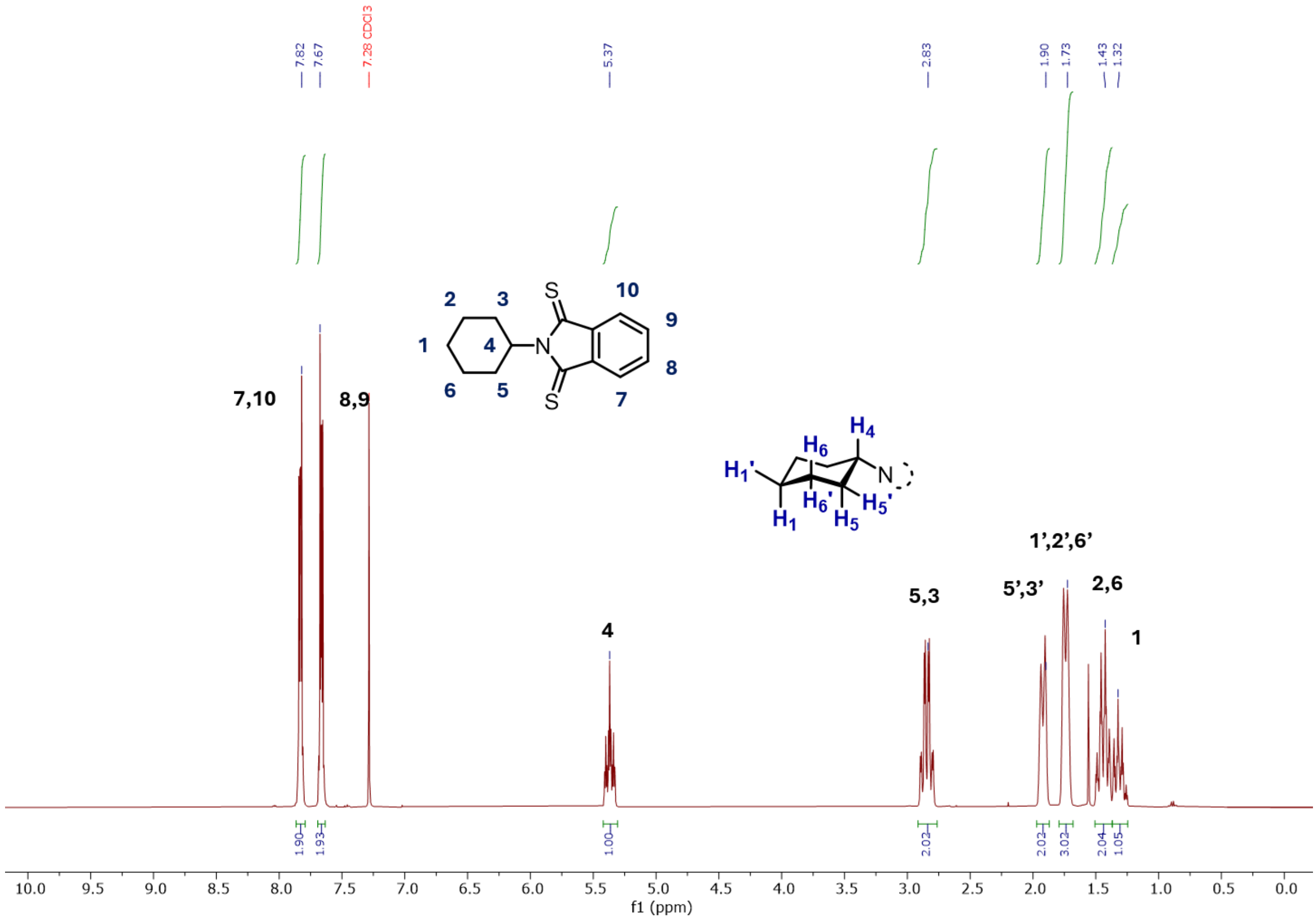

Figure S22: *^{1}H NMR spectra of **3** ($CDCl_3$, 400 MHz).*

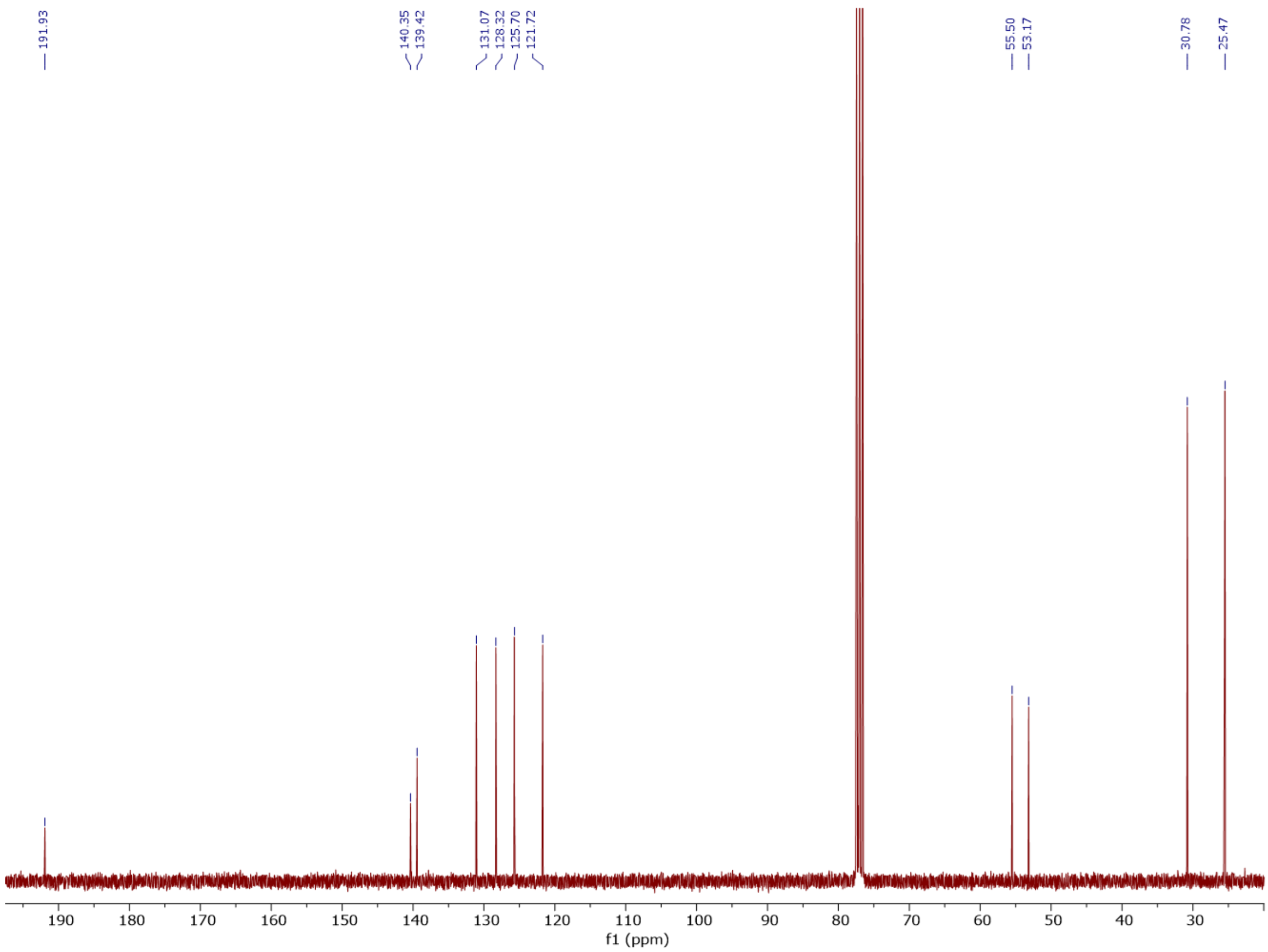

Figure S23: *^{13}C NMR spectra of **3** ($CDCl_3$, 101 MHz).*

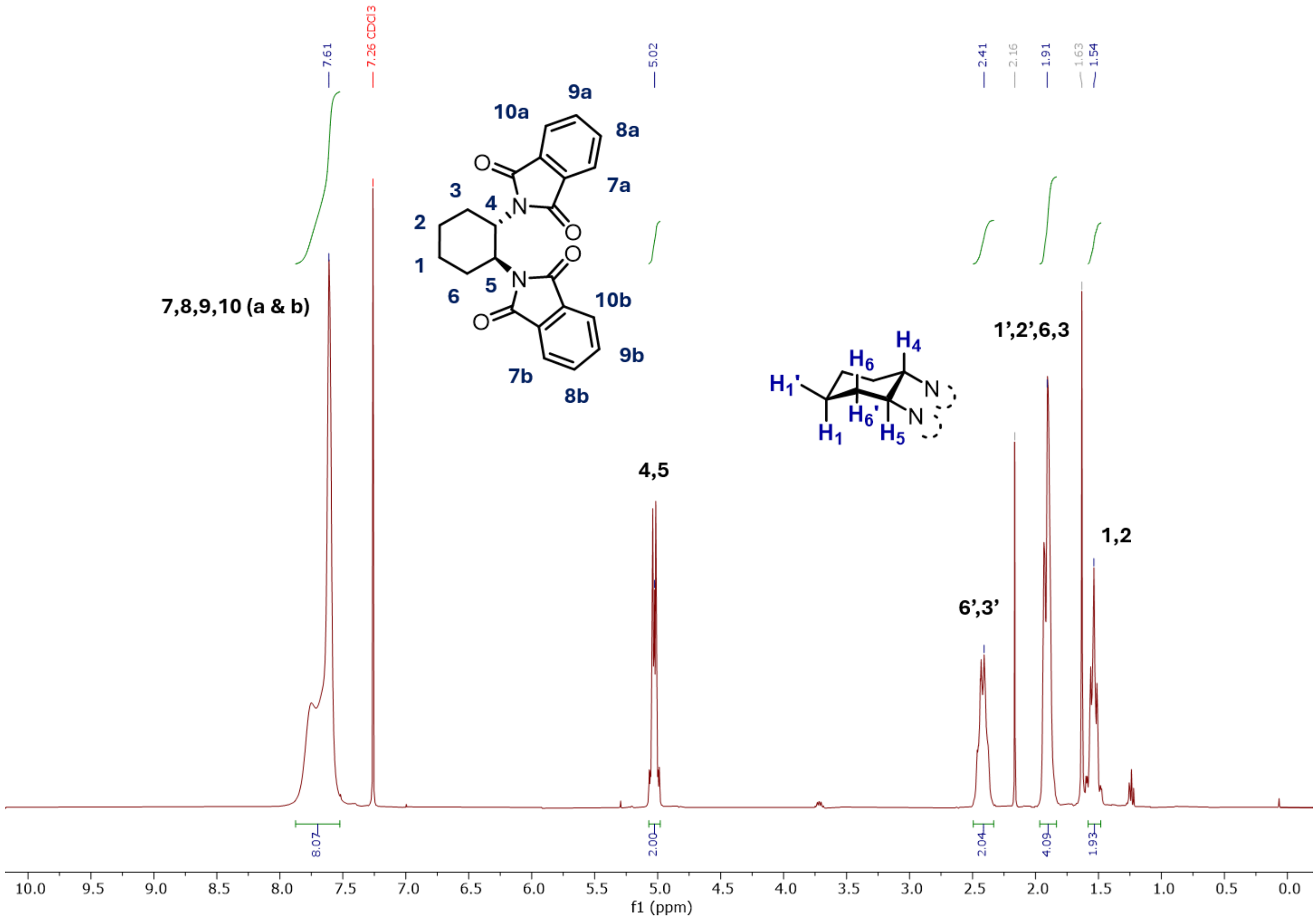

Figure S24: *^{1}H NMR spectra of **4** ($CDCl_3$, 400 MHz).*

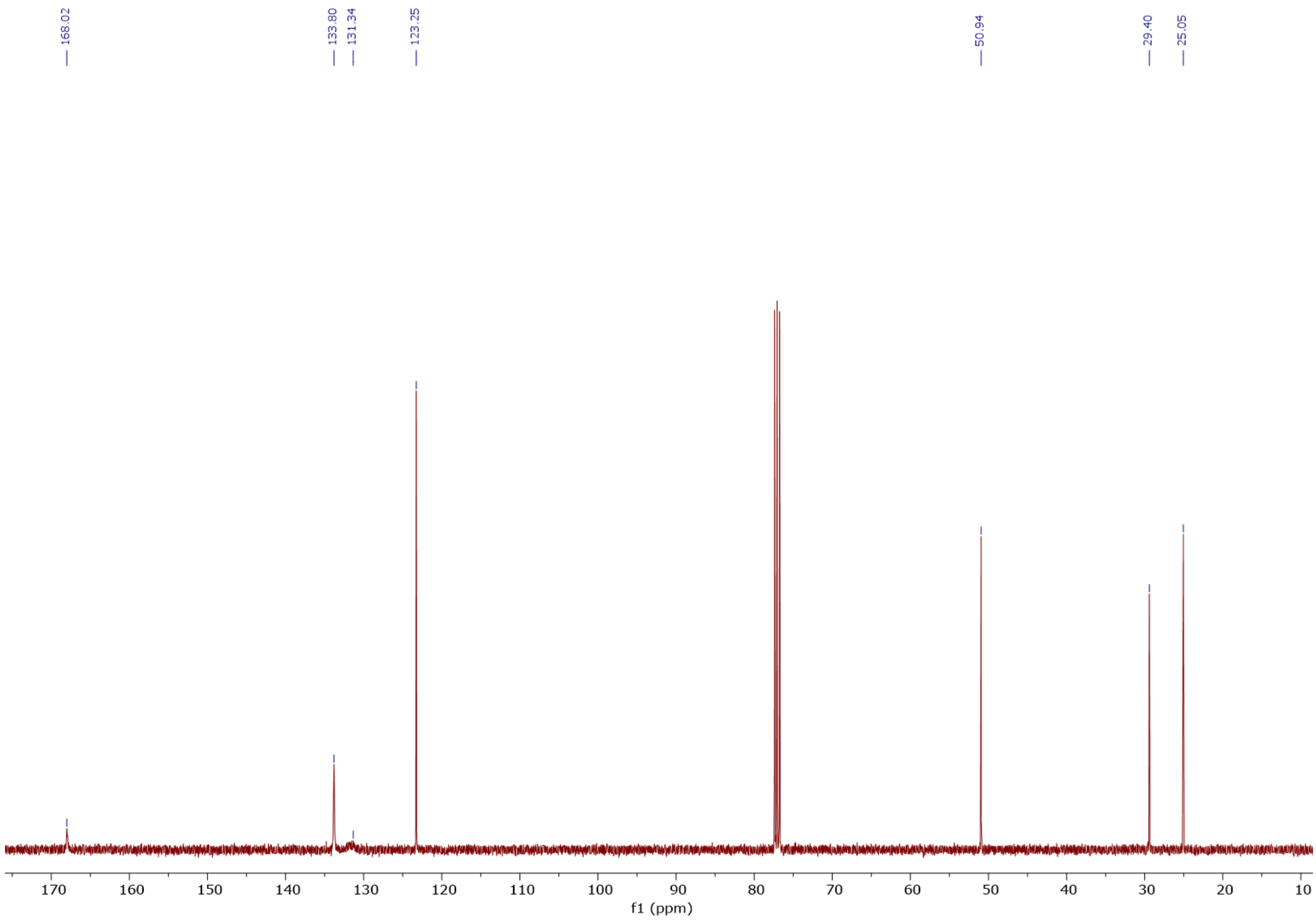

Figure S25: *^{13}C NMR spectra of **4** ($CDCl_3$, 101 MHz).*

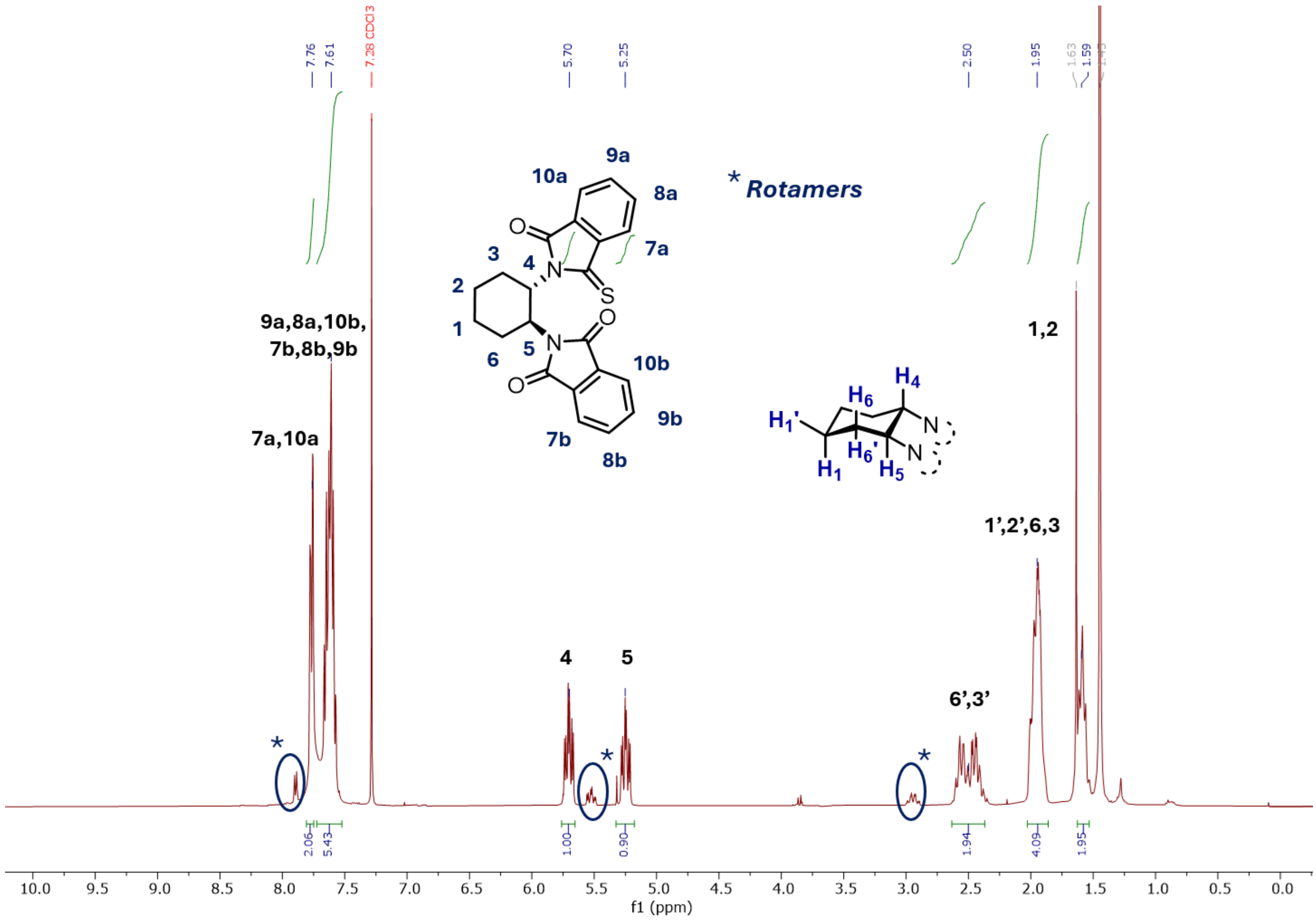

Figure S26: *^{1}H NMR spectra of* ***5*** *($CDCl_3$, 400 MHz).*

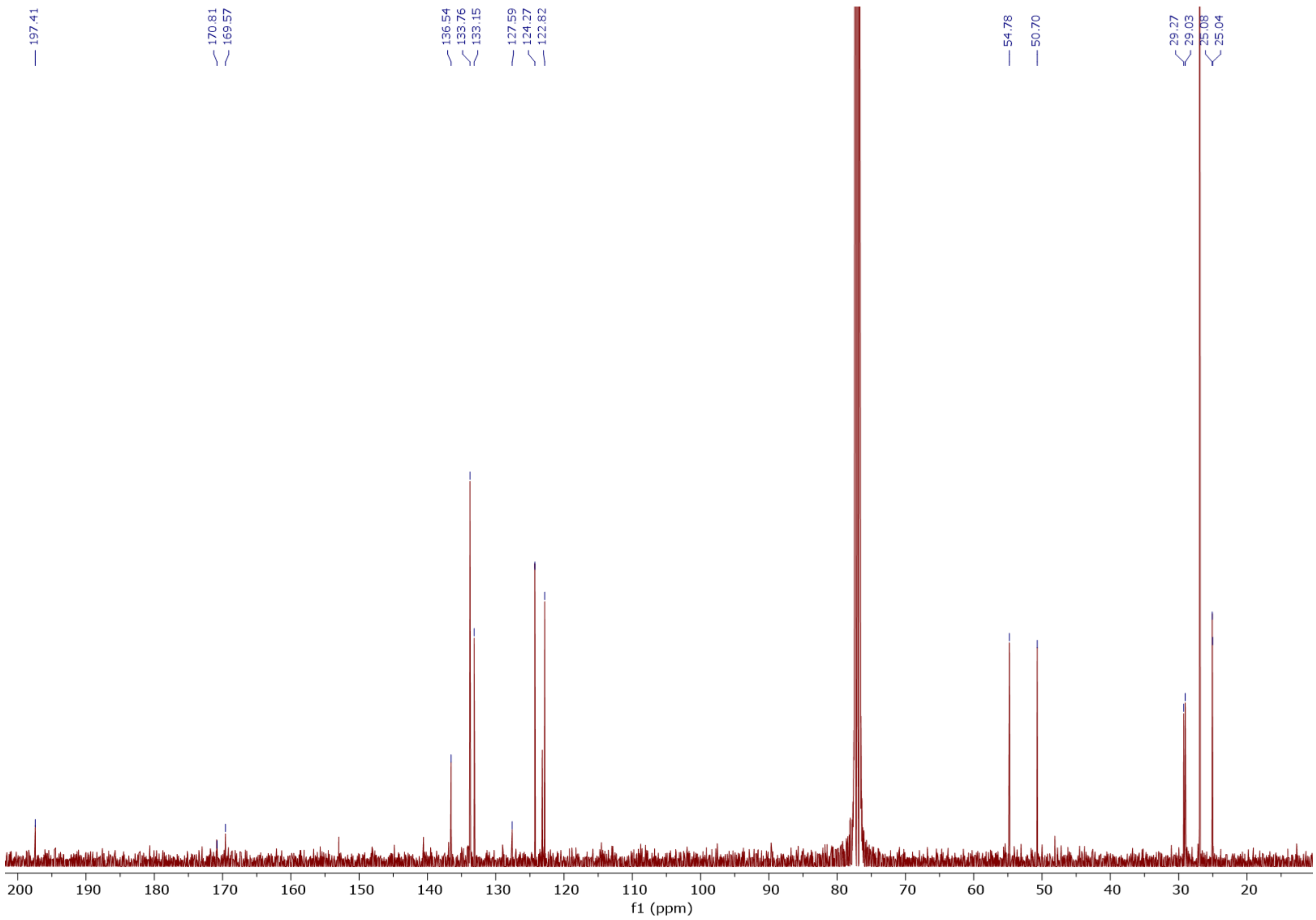

Figure S27: *^{13}C NMR spectra of* ***5*** *($CDCl_3$, 101 MHz).*

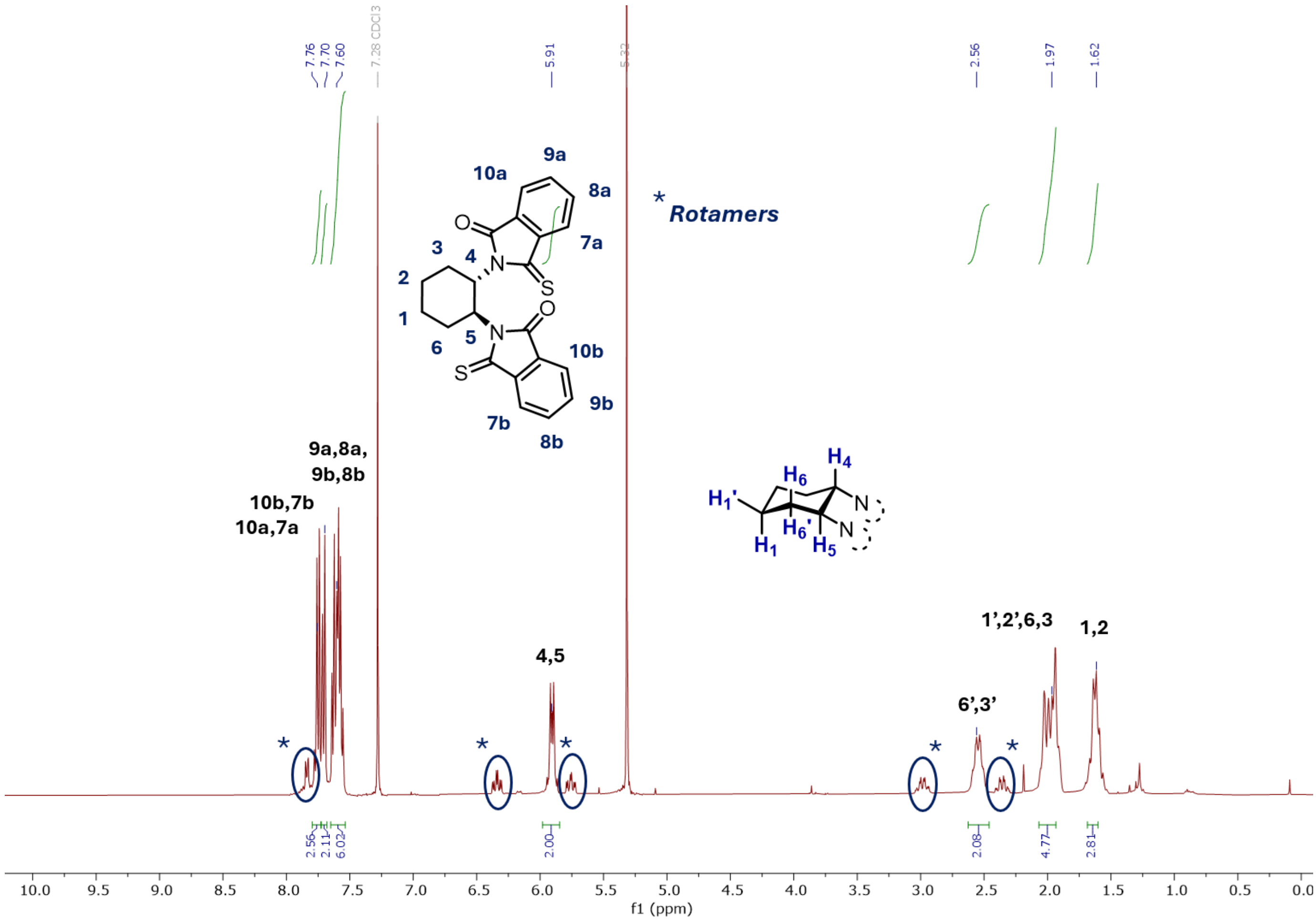

Figure S28: *^{1}H NMR spectra of **6** ($CDCl_3$, 400 MHz).*

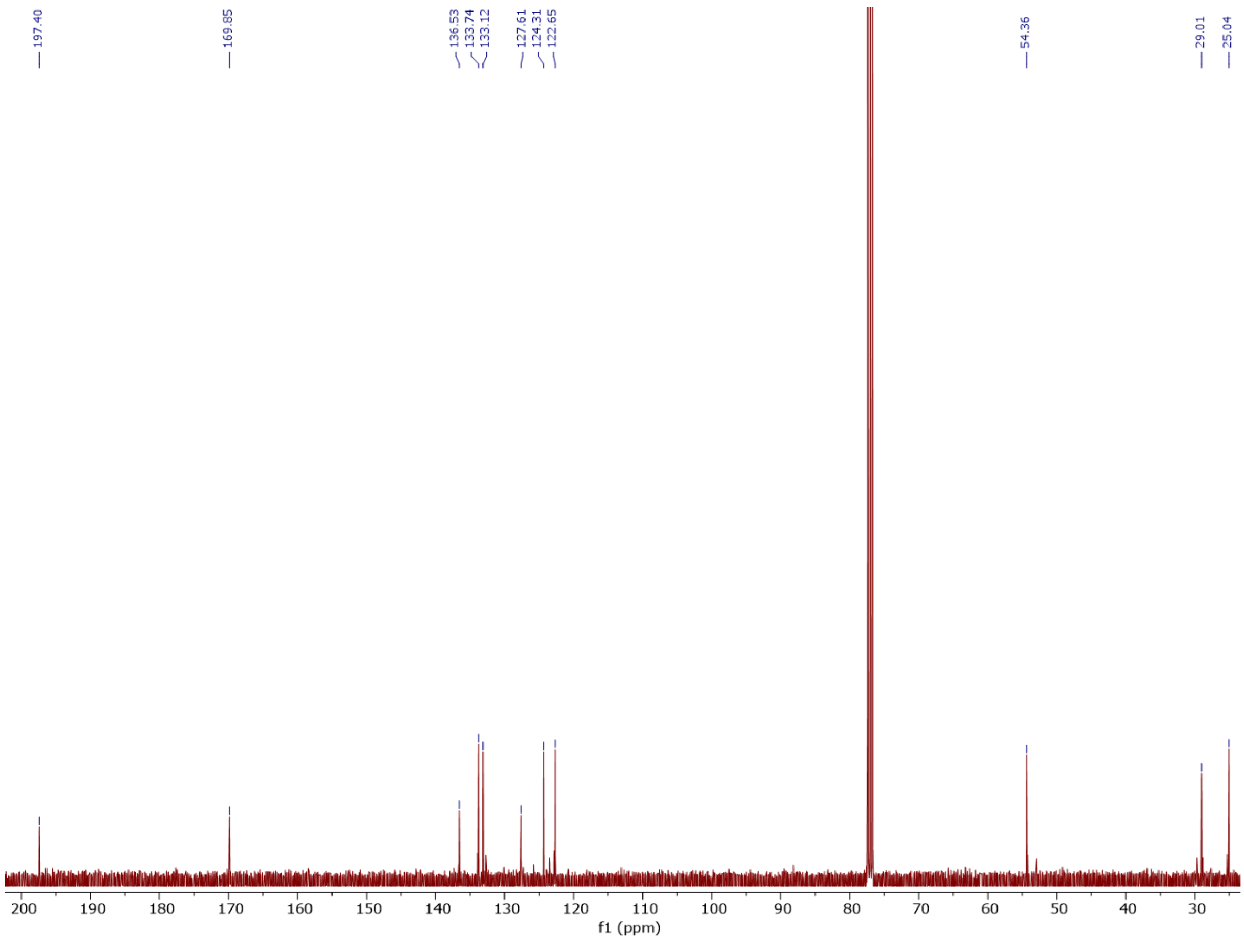

Figure S29: *^{13}C NMR spectra of **6** ($CDCl_3$, 101 MHz).*

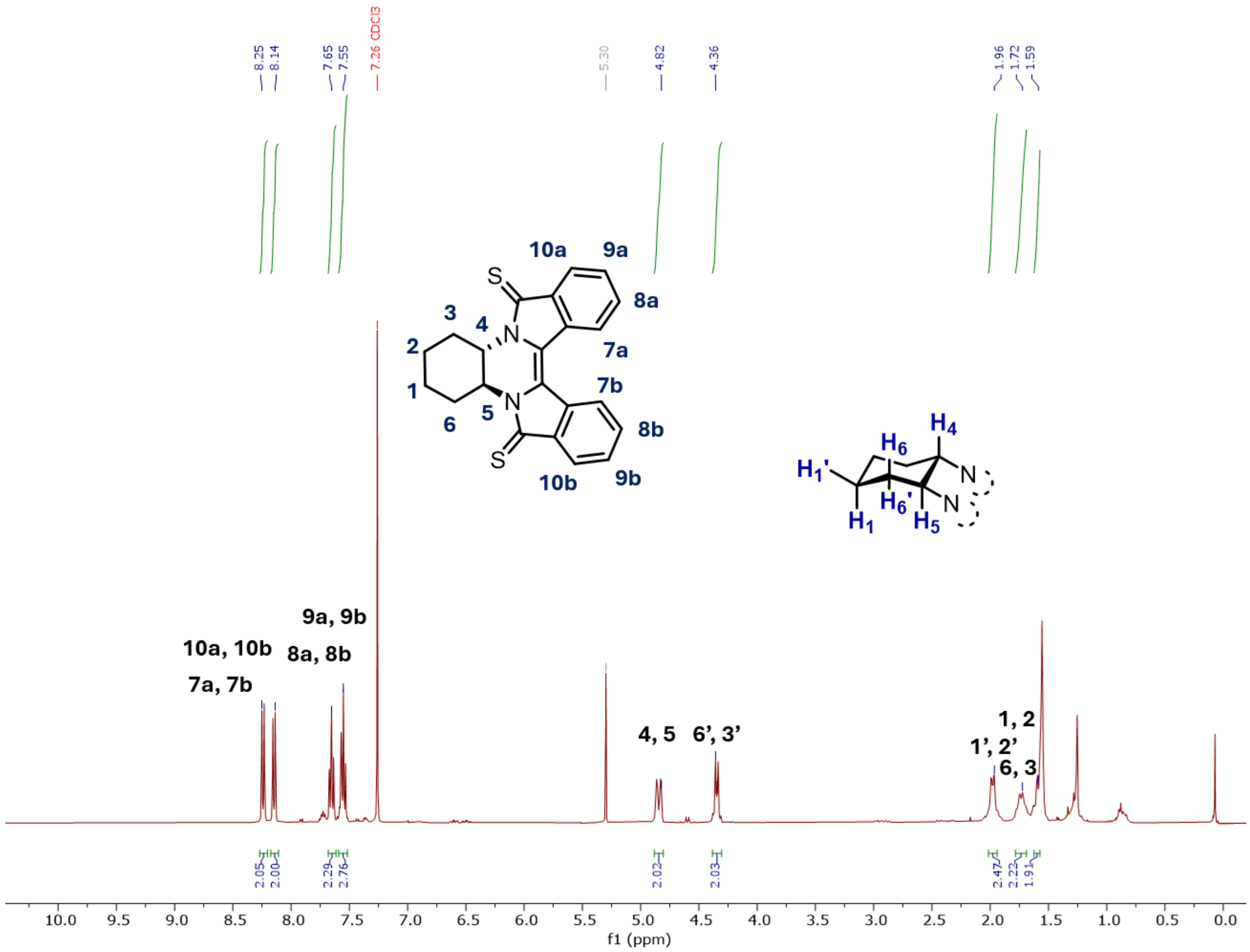

Figure S30: ^{1}H NMR spectra of **7** ($CDCl_3$, 400 MHz).

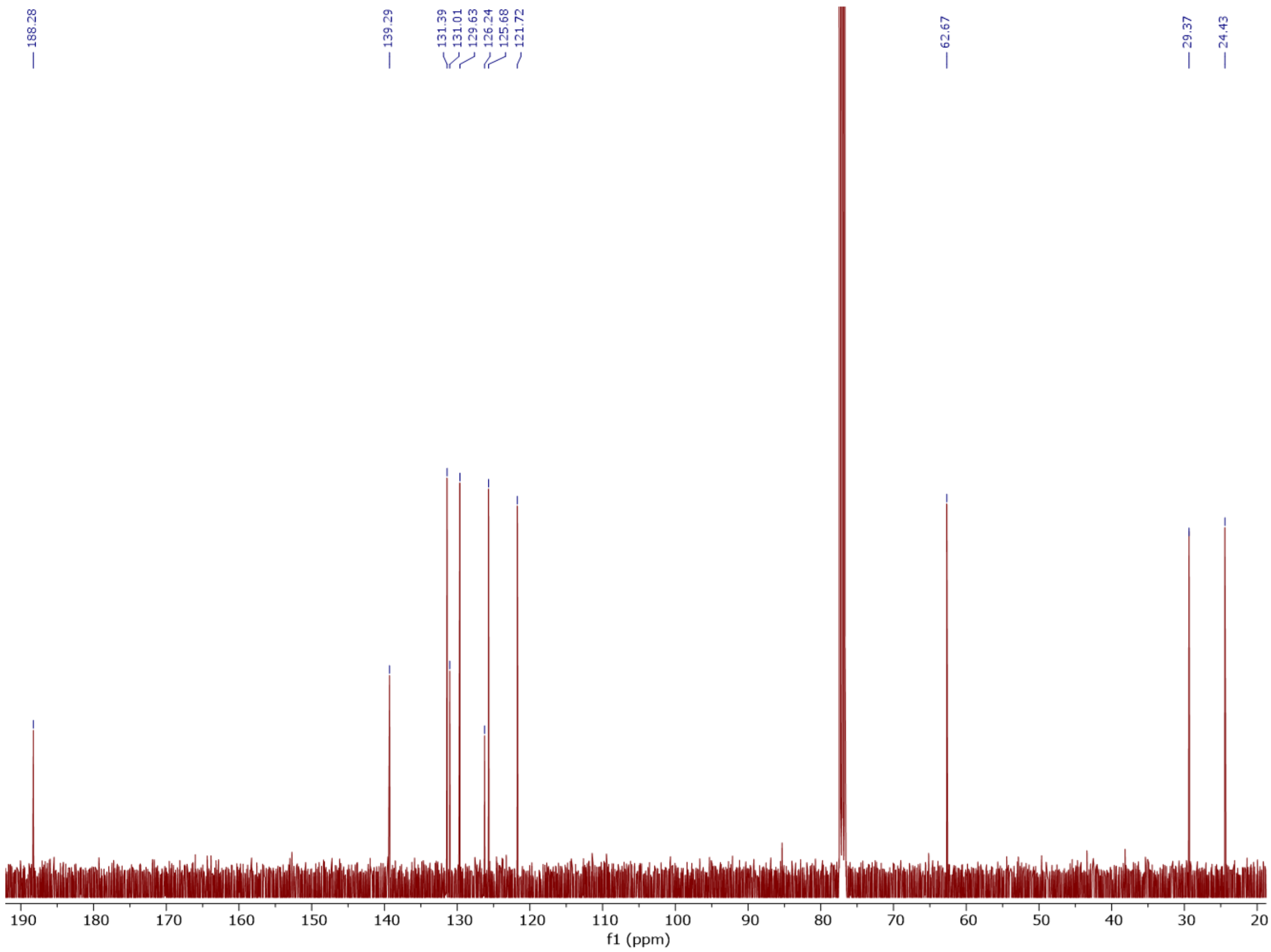

Figure S31: ^{13}C NMR spectra of **7** ($CDCl_3$, 101 MHz).

8. HR-MS Spectra

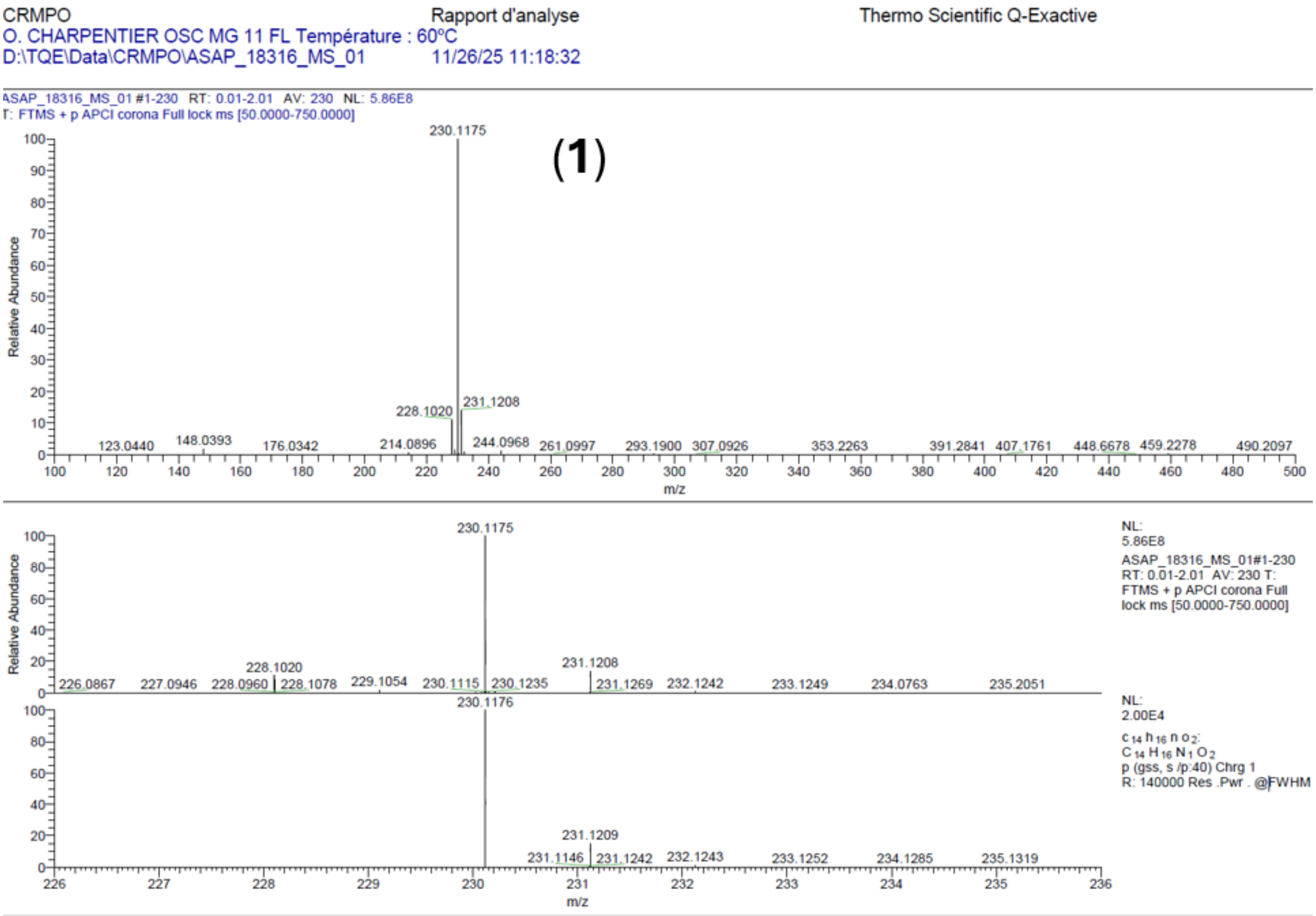

Figure S32: HRMS spectrum of compound **1**.

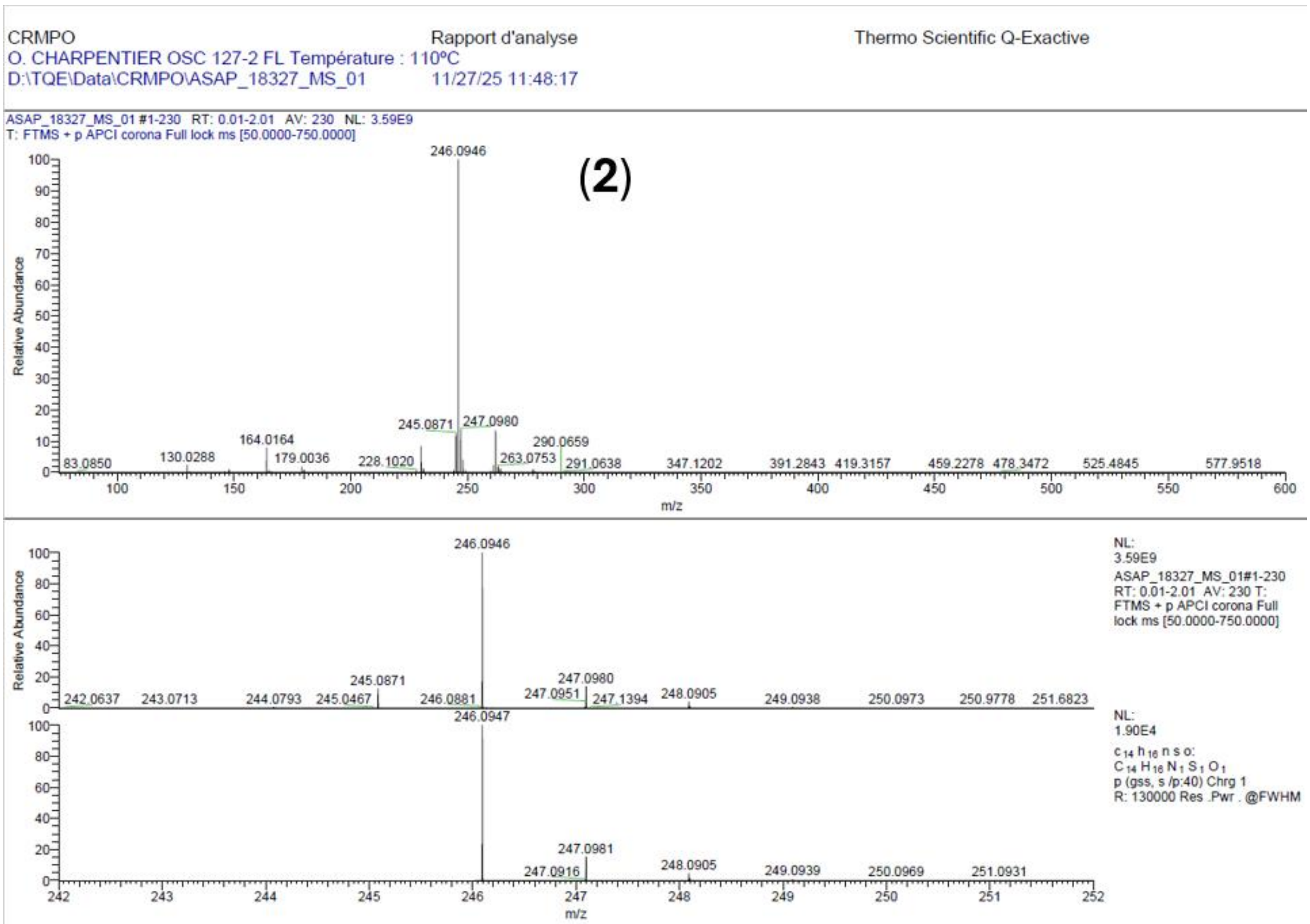

Figure S33: HRMS spectrum of compound **2**.

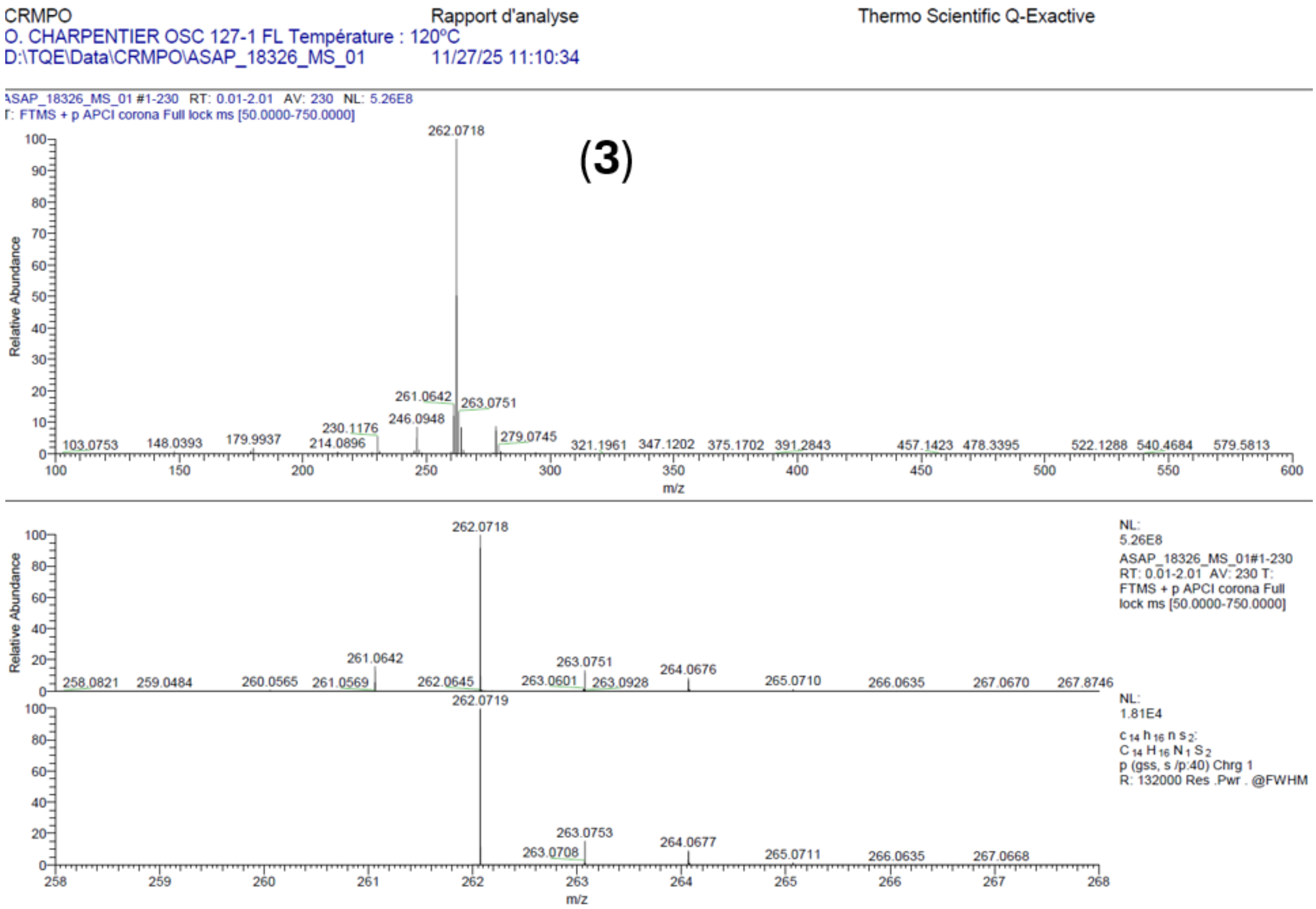

Figure S34: HRMS spectrum of compound **3**.

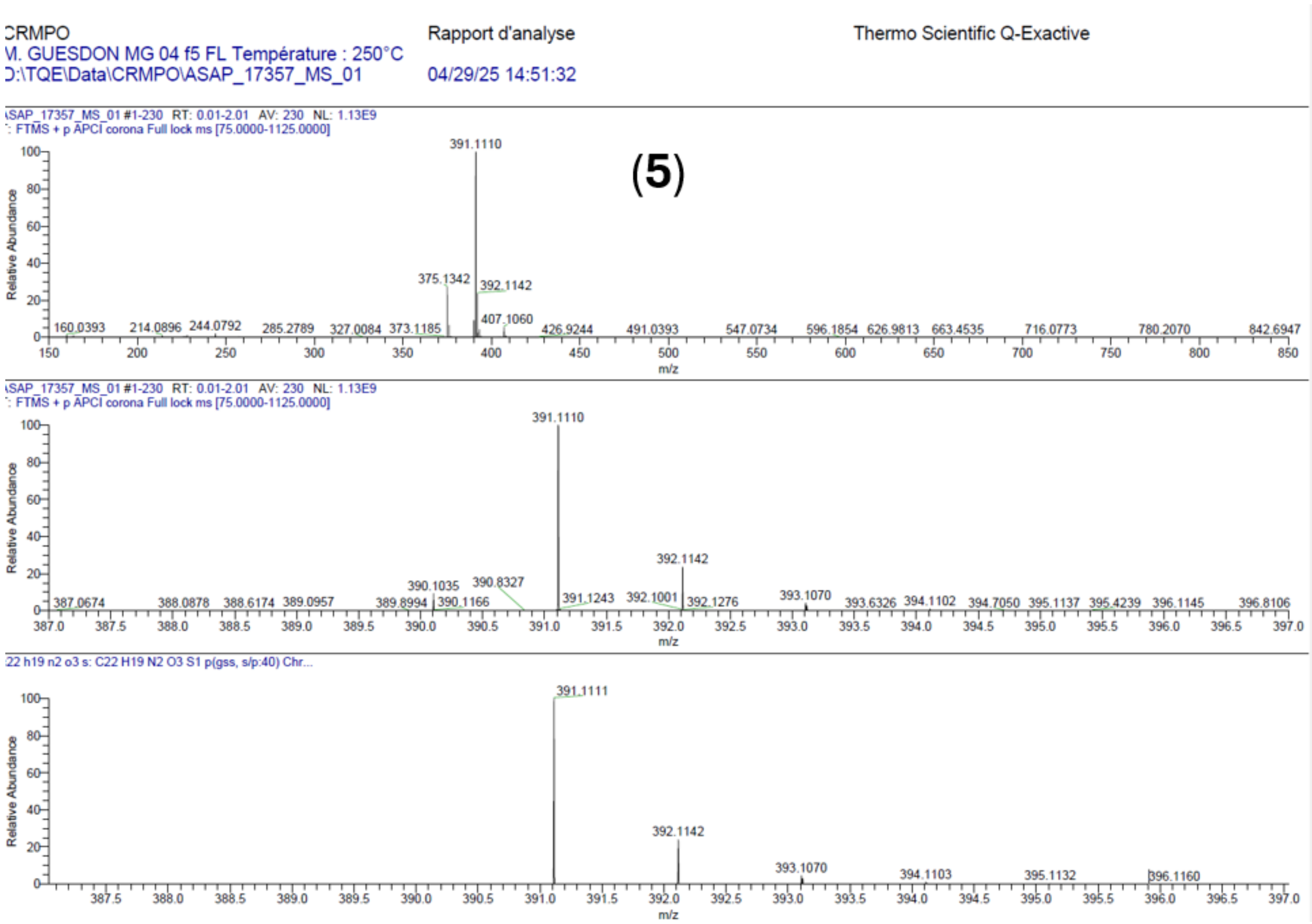

Figure S35: HRMS spectrum of compound **5**.

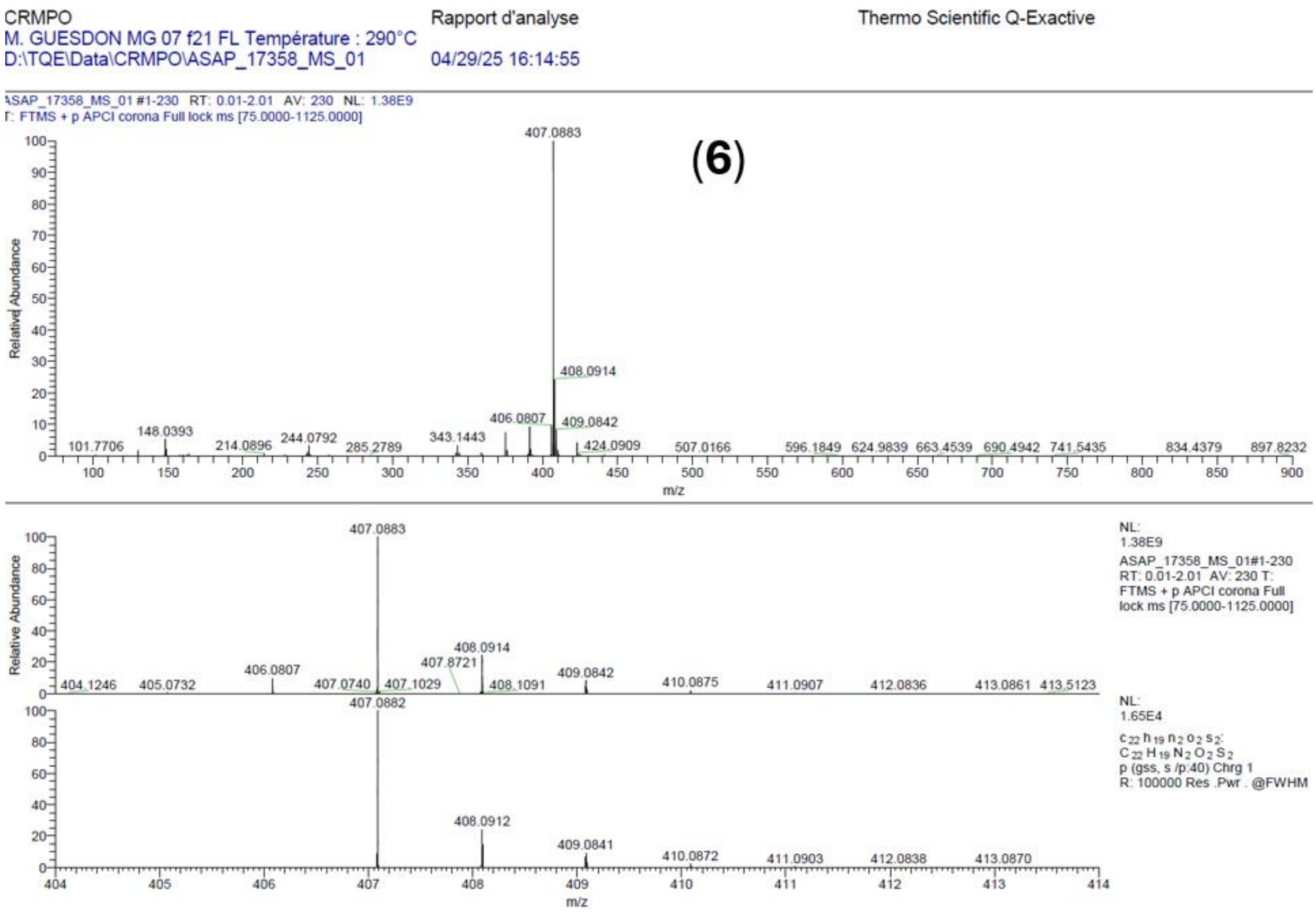

Figure S36: HRMS spectrum of compound **6**.

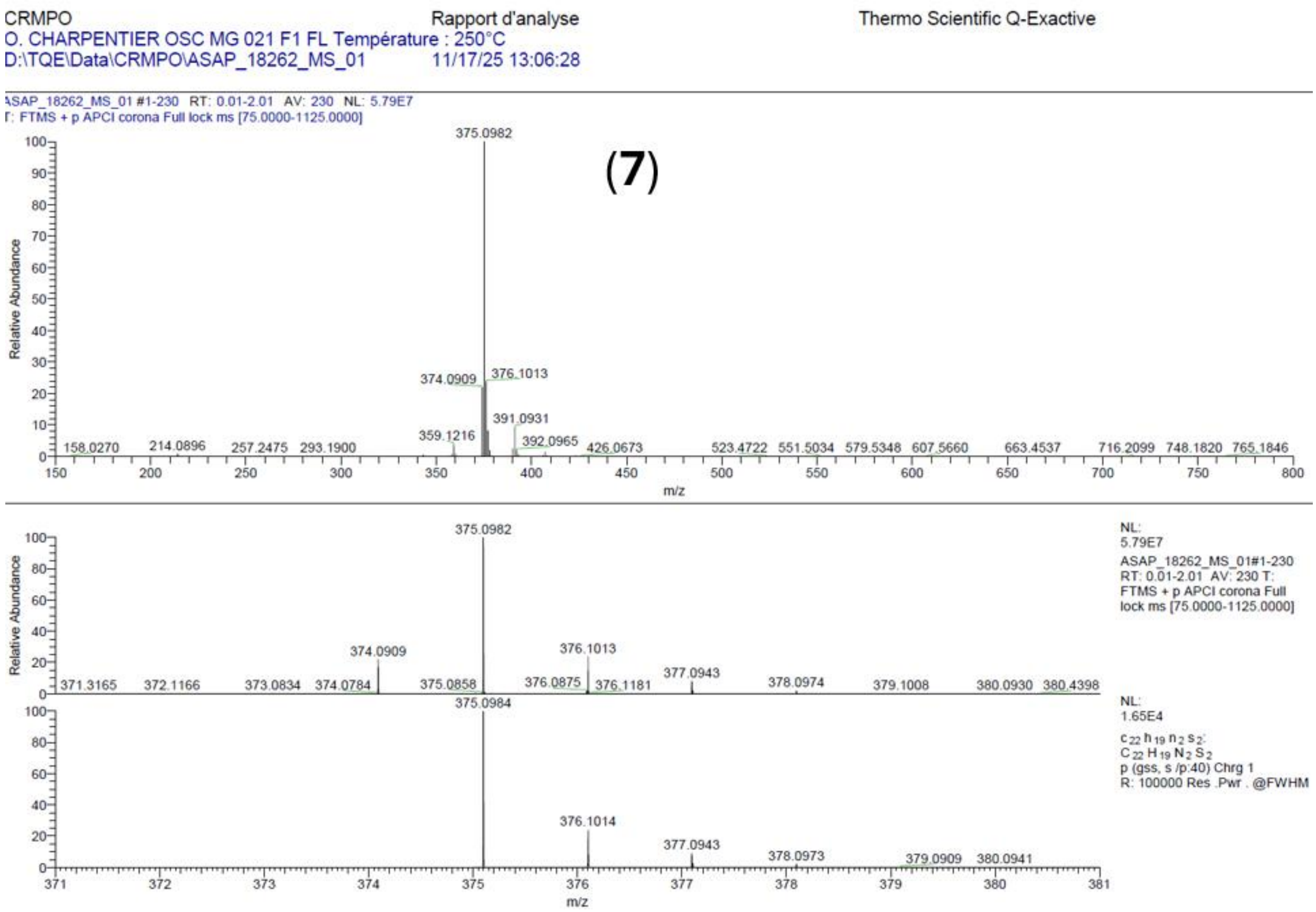

Figure S37: HRMS spectrum of compound **7**.

9. Additional theoretical results

a) Representation of the conformers

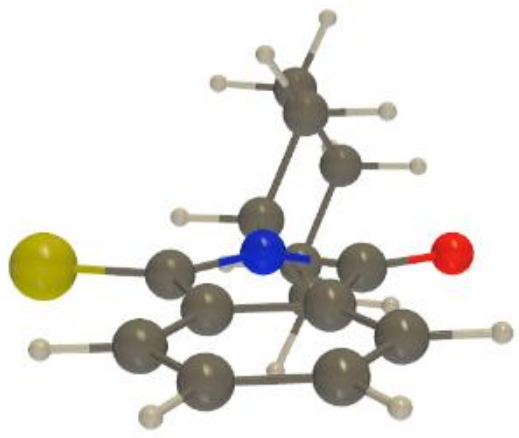

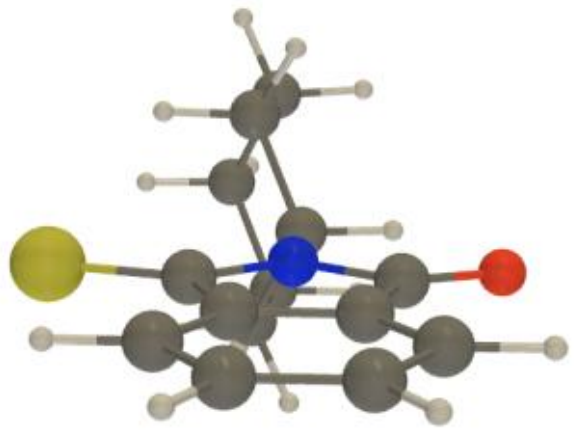

G=0.0 kcal.mol^{-1} *G*=1.3 kcal.mol^{-1}

Figure S38: *Representation of the two conformers of* ***2*** *together with their relative free energies in kcal.mol^{-1}. Note the different orientations of the cyclohexyl.*

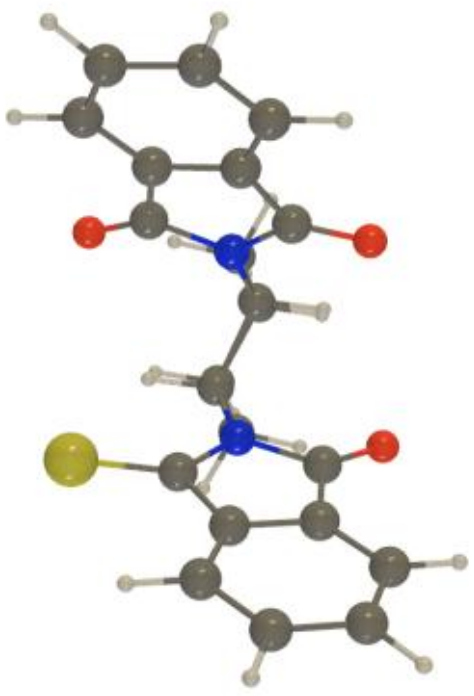

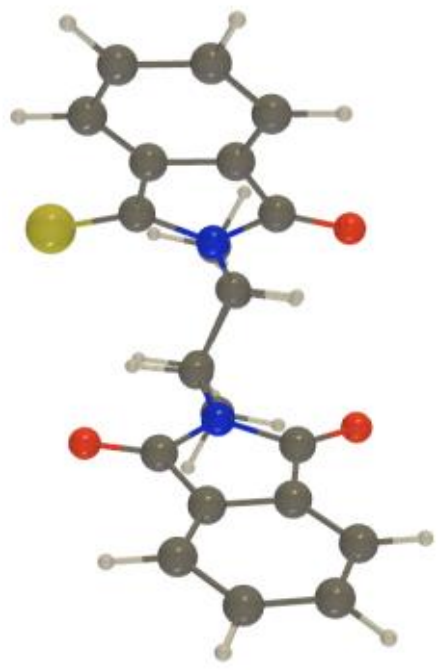

G=0.0 kcal.mol^{-1} *G*=1.2 kcal.mol^{-1}

Figure S39: *Representation of the two conformers of* ***5*** *together with their relative free energies in kcal.mol^{-1}.*

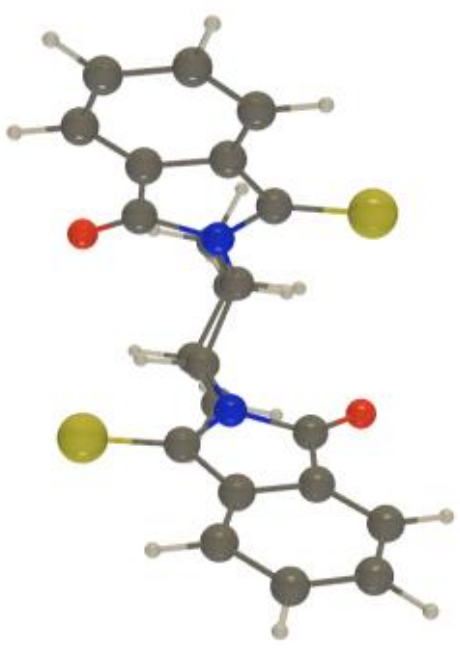

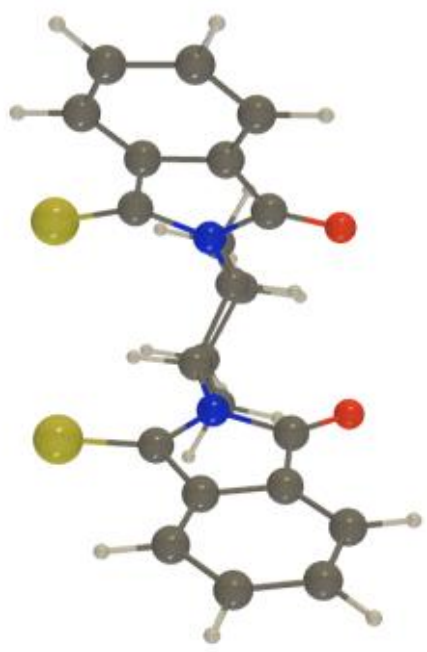

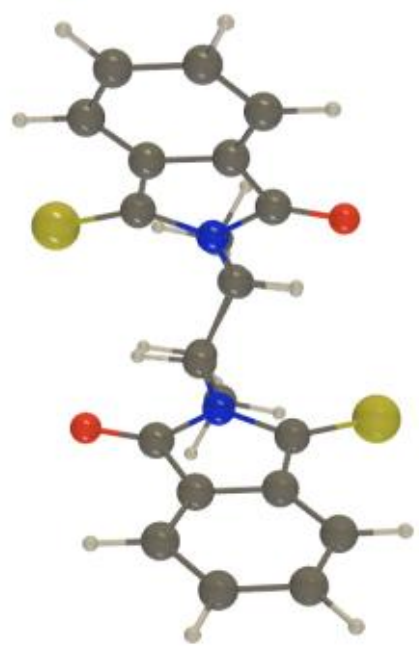

G=0.0 kcal.mol^{-1} *G*=0.8 kcal.mol^{-1} *G*=2.6 kcal.mol^{-1}

Figure S40: *Representation of the three conformers of* ***6*** *together with their relative free energies in kcal.mol^{-1}.*

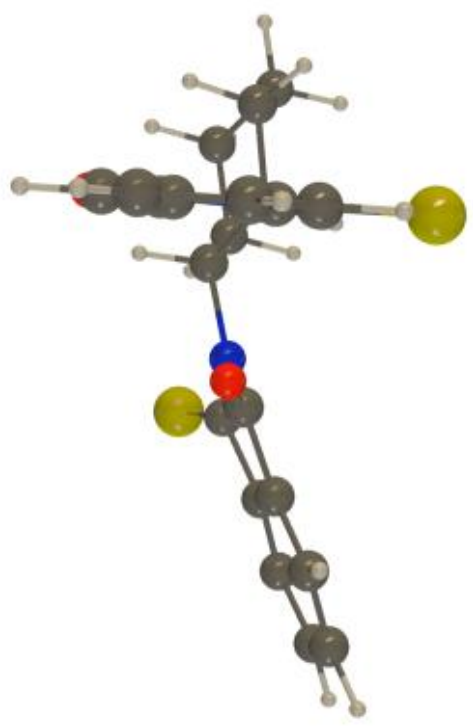

G=21.0 kcal.mol^{-1}

Figure S41: *Representation of the transition state between the two most stable conformers of* ***6****. This transition state presents an imaginary frequency of 49i cm^{-1}. The reported free energy is compared to the most stable structure.*

b) Simulated spectra

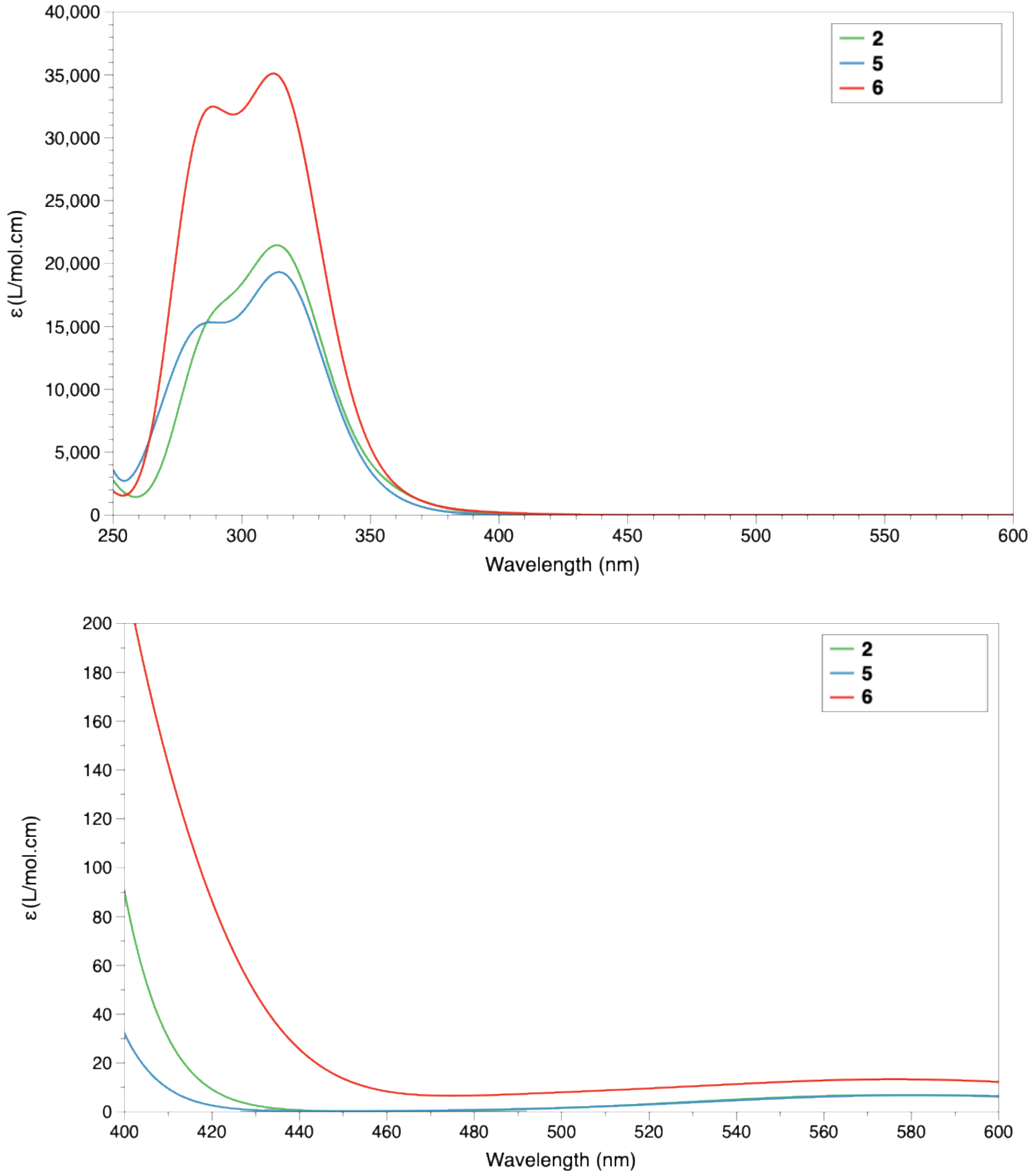

Figure S42: *Modelled UV/Vis spectrum for* ***2***, ***5***, *and* ***6****: general view (top) and zoom on the long wavelength region (bottom). The intensities are determined on the basis of vertical energies and oscillator strength obtained at the (LR-)SMD($CHCl_3$)-ZORA-PBE0/ma-def2-TVPP level and include SOC effects but do not account for vibronic couplings.*

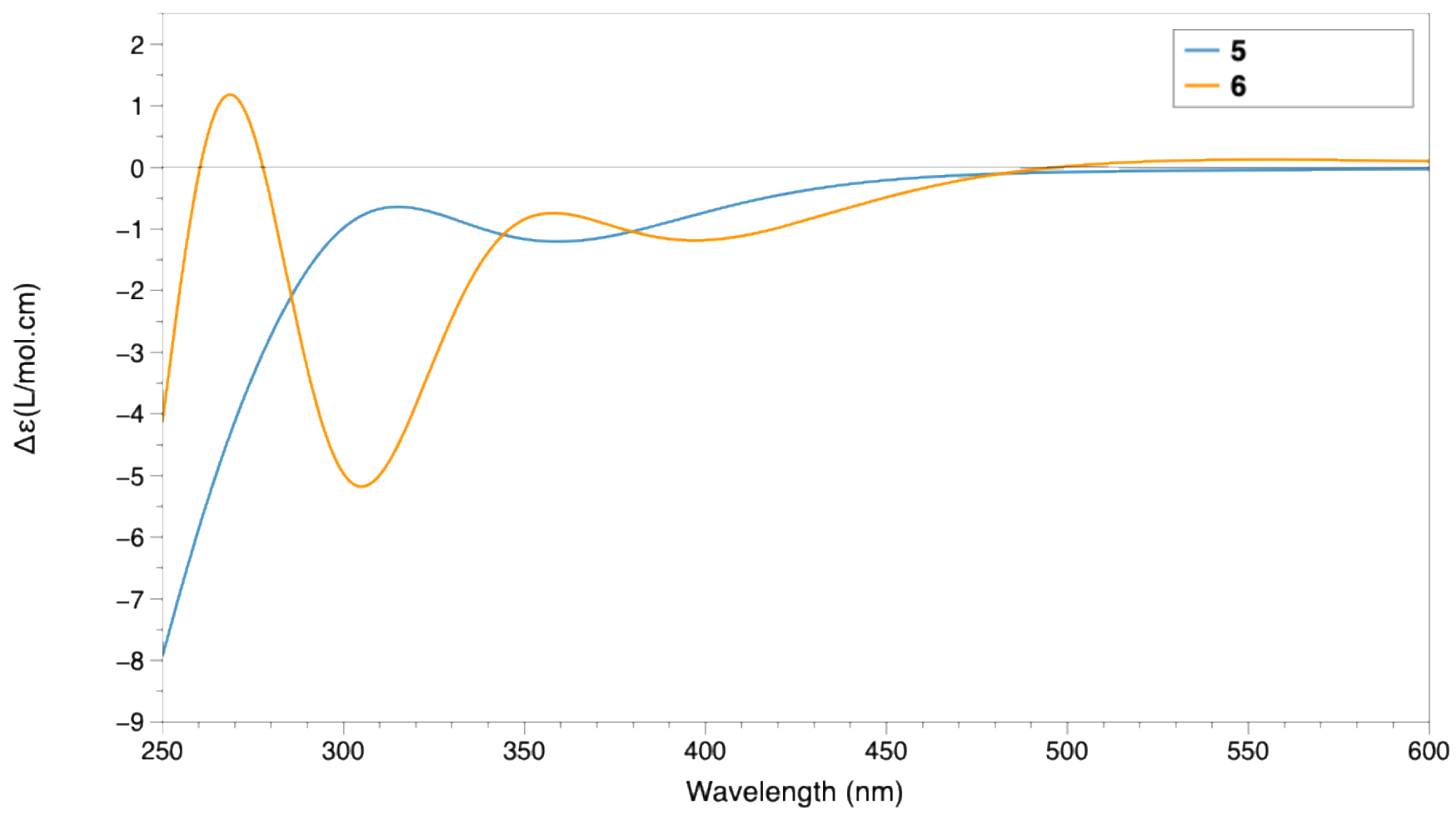

Figure S43: *Modelled ECD spectrum for* ***5*** *(R,R) and (R,R)* ***6****.*

c) Electron Density Difference plots

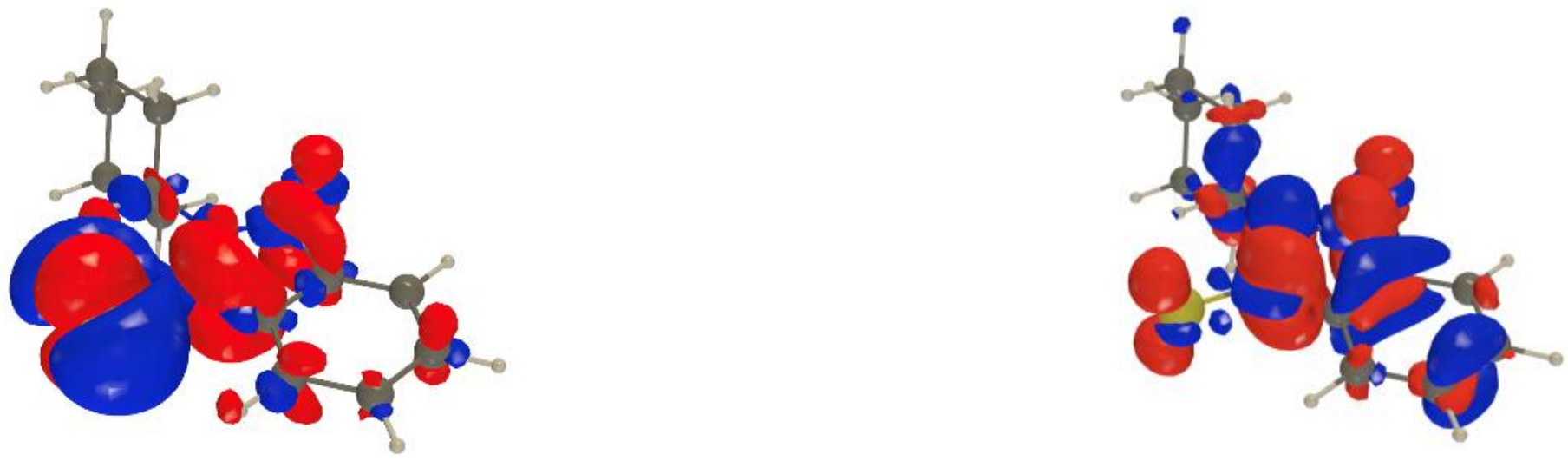

2 (S_0- S_1) **2** (S_0- S_2)

Figure S44: *Electron density difference (EDD) plots corresponding to the absorption to the lowest singlet excited states of **2** (ground-state geometry). The blue and red lobes represent regions of decrease and increase of density (threshold: 0.002 au). PCM-PBE0/6-311++G(d,p) level of theory.*

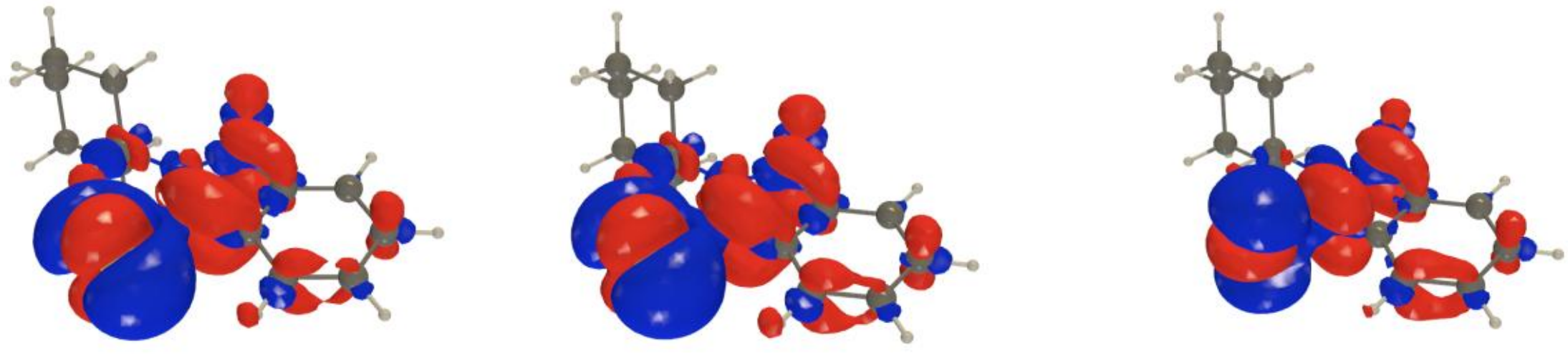

2 (S_0- S_1) **2** (S_0- T_1) **2** (S_0- T_2)

Figure S45: *Electron density difference (EDD) plots corresponding to the absorption to the lowest singlet and the two lowest triplet excited states of **2** (S_1 optimal geometry). See caption above for the color code and threshold.*

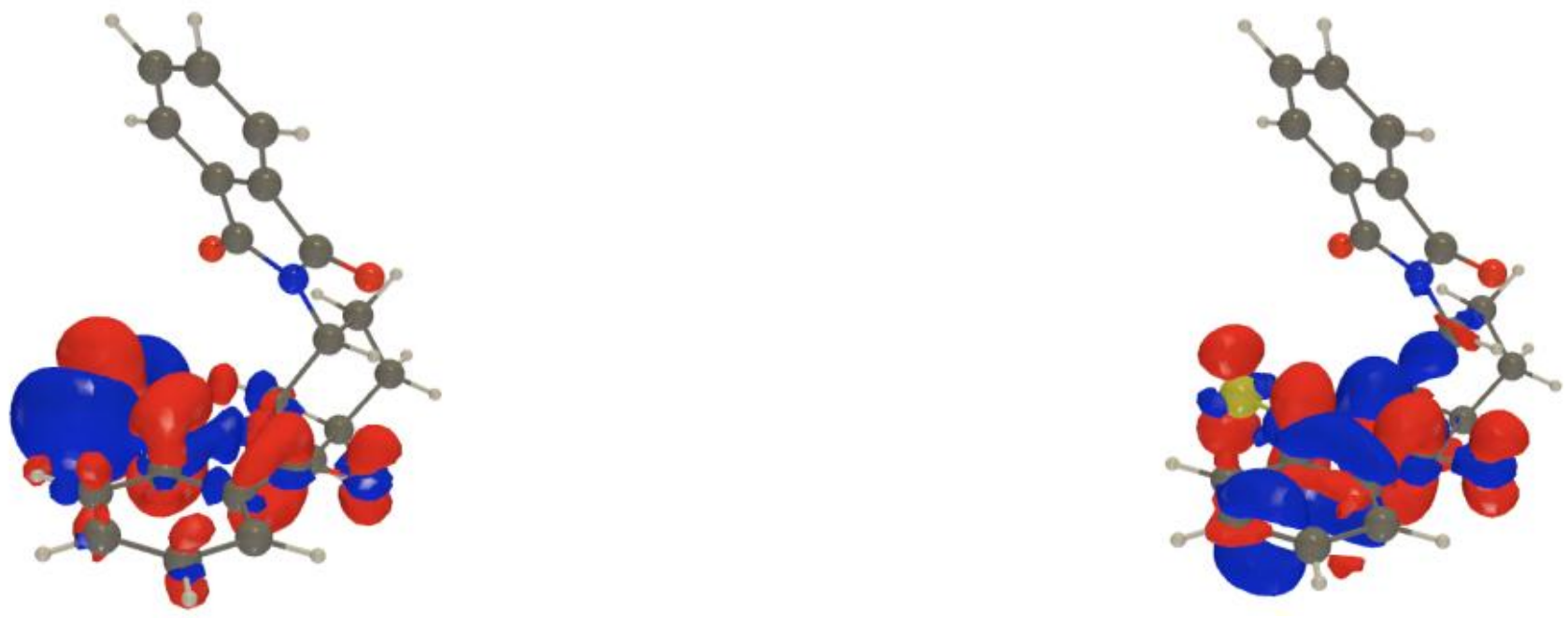

5 (S_0- S_1) **5** (S_0- S_2)

Figure S46: *Electron density difference (EDD) plots corresponding to the absorption to the lowest singlet excited states of **5** (ground-state geometry). See caption above for the color code and threshold.*

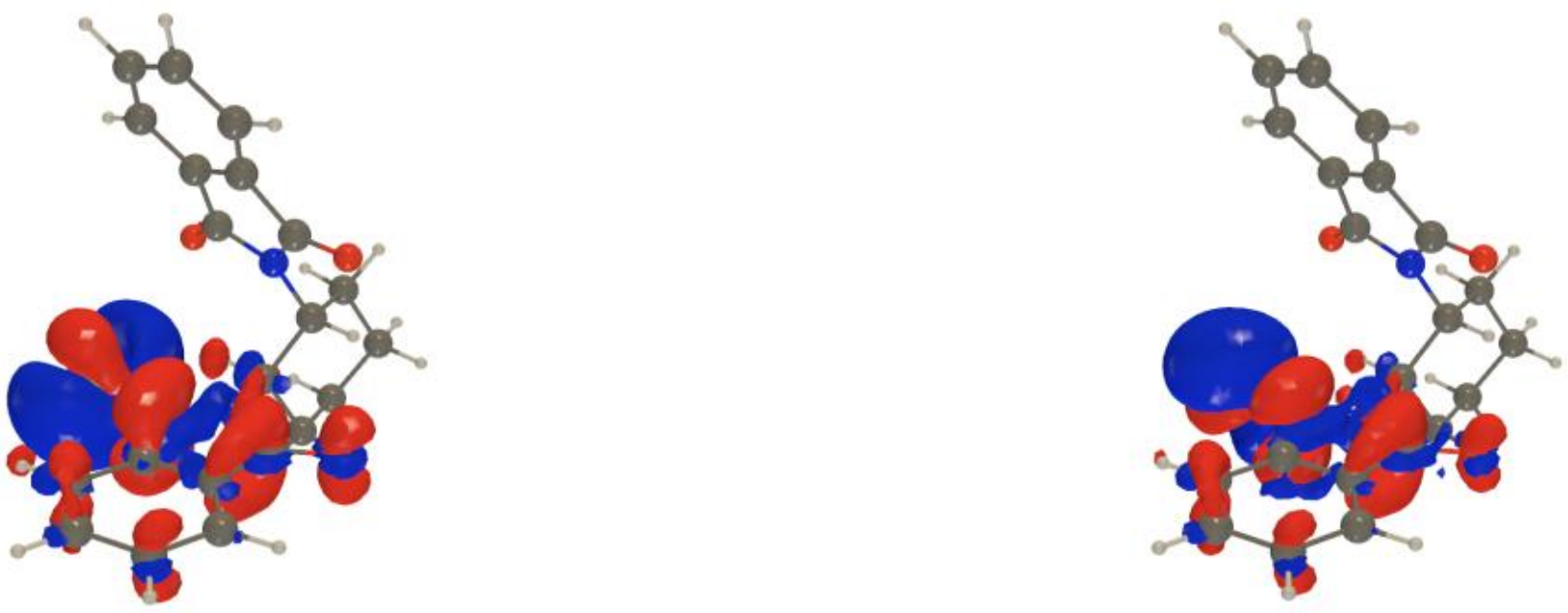

5 (S_0- T_1) **5** (S_0- T_2)

Figure S47: *Electron density difference (EDD) plots corresponding to the absorption to the lowest triplet excited states of **5** (S_1 optimal geometry). See caption above for the color code and threshold*

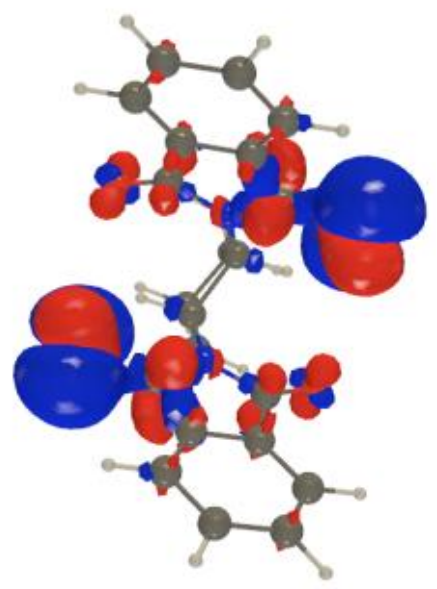

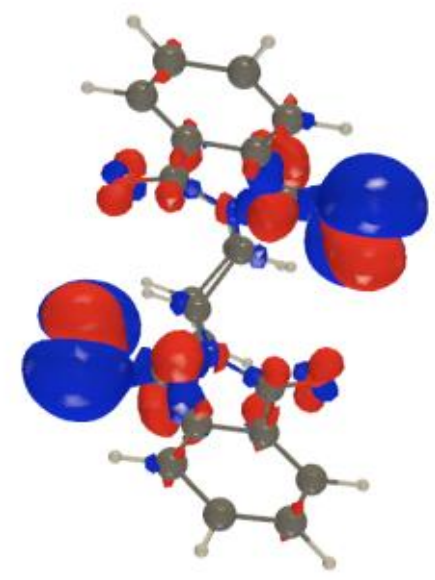

6 (S_0- S_1) **6** (S_0- S_2)

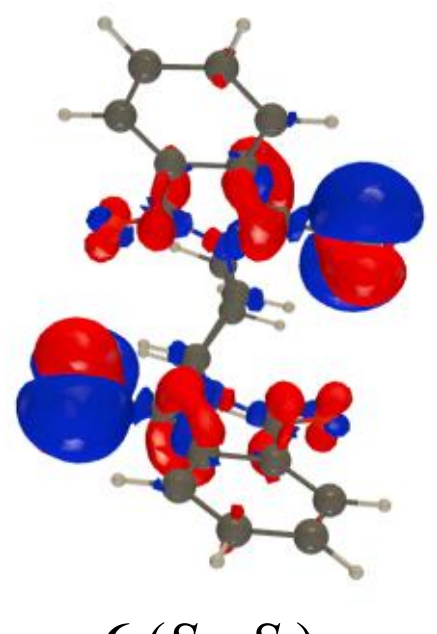

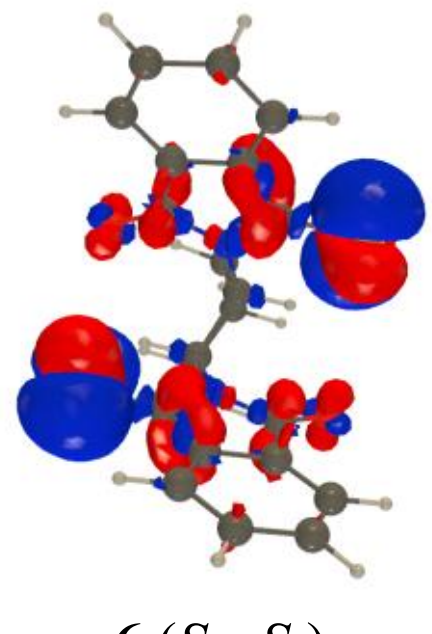

6 (S_0- S_3) **6** (S_0- S_4)

Figure S48: *Electron density difference (EDD) plots corresponding to the absorption to the lowest singlet excited states of **6** (ground-state geometry). See caption above for the color code and threshold.*

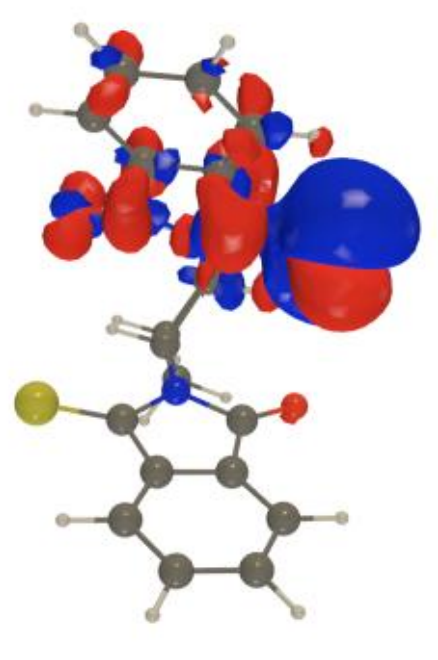

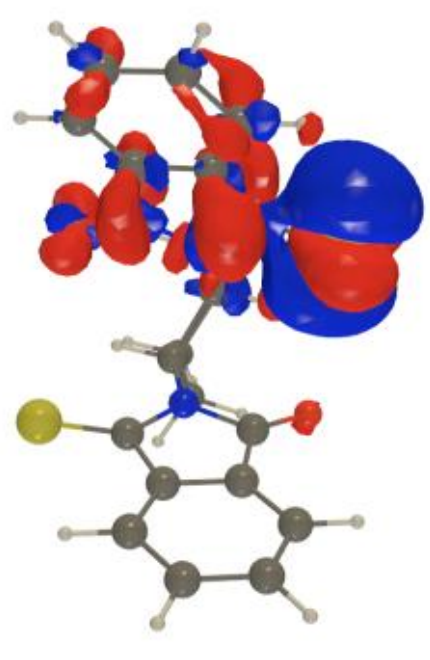

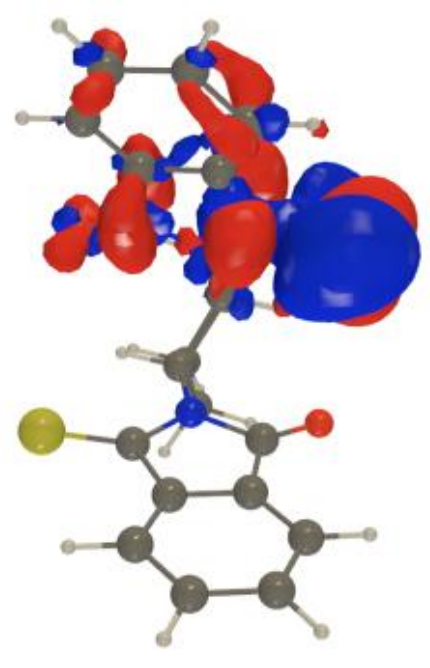

6 (S_0- S_1) **6** (S_0- T_1) **6** (S_0- T_2)

Figure S49: *Electron density difference (EDD) plots corresponding to the absorption to the lowest singlet and two lowest triplet excited states of **6** (S_1 optimal geometry). See caption above for the color code and threshold*

d) Spin Difference plots

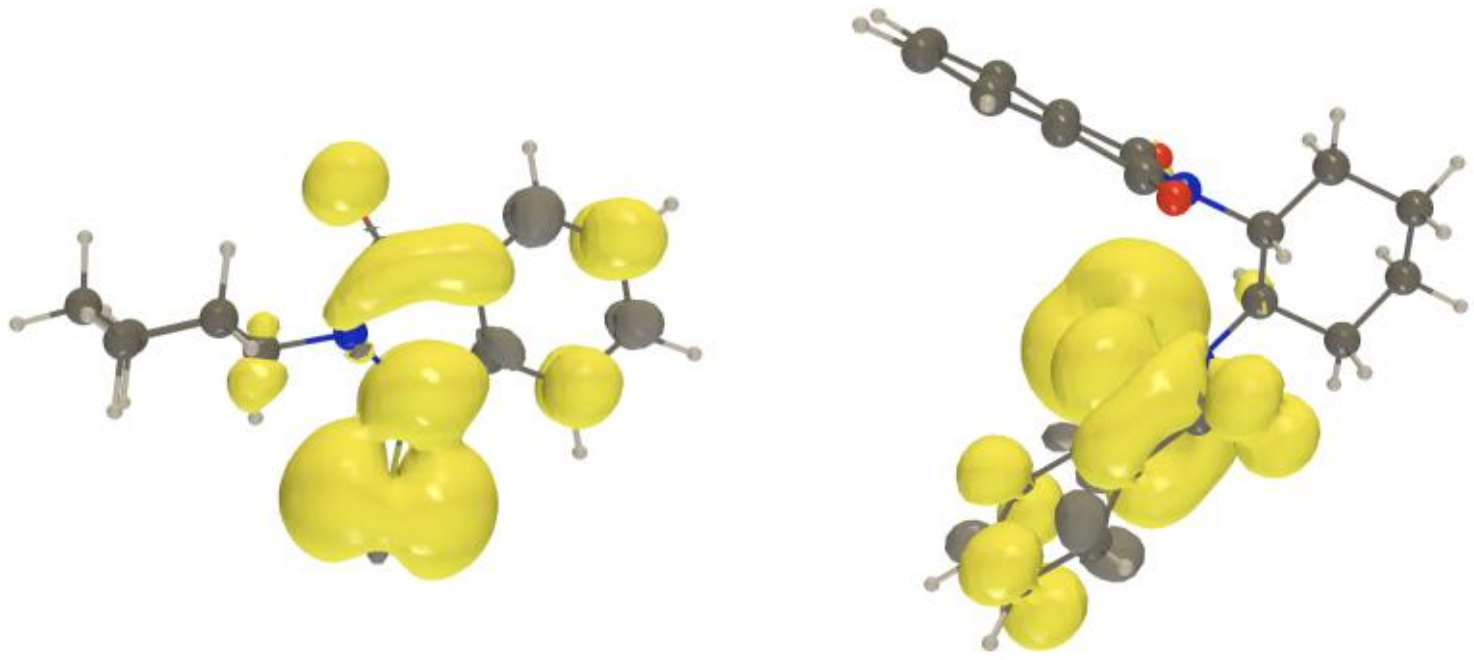

2 **5**

Figure S50: *Spin difference plots (α–β) plots corresponding to the lowest triplet excited state (threshold: 0.002 au) for **2** and **5**. PCM-UPBE0/6-311++G(d,p) level of theory.*

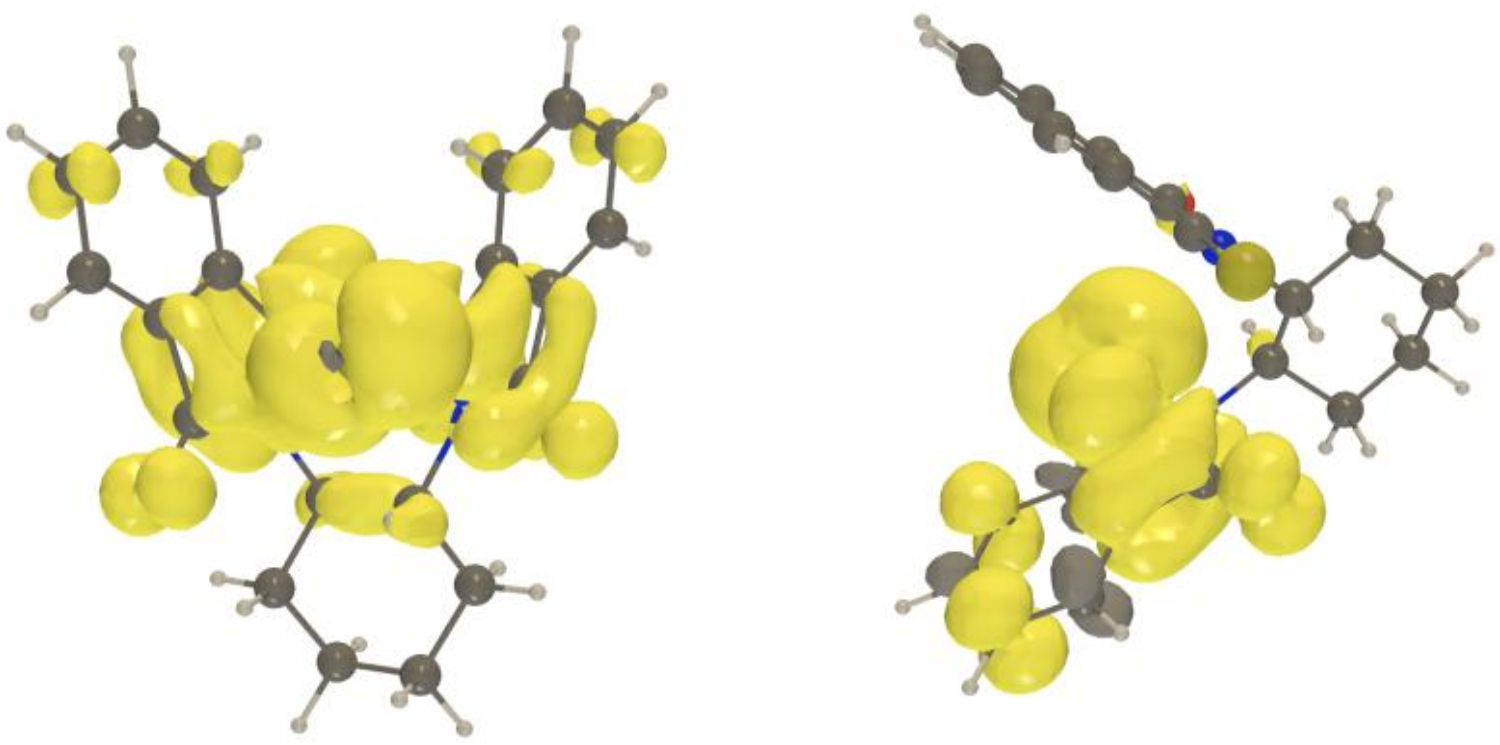

6 (sym) **6 (non-sym)**

Figure S51: *Spin difference plots (α–β) plots corresponding to the lowest triplet excited state of **6** (threshold: 0.002 au). We obtained two triplets, the non-symmetric one being more stable by 11.4 kcal.mol^{-1} on the free energy scale (both are true minima according to U-DFT).*

e) Computed transition energies and SOCs

Table. S3: *Theoretical best estimates of the vertical absorption and fluorescence wavelengths (in nm) and 0-0 energies (in eV) for all compounds. These values are CC2 corrected.*

		$\lambda^{vert\text{-}abs}$ (nm)	$\lambda^{vert\text{-}flu}$ (nm)	ΔE^{0-0} (eV)
**2**	S_1	470	589	2.25
	S_2	344	454	2.98
**5**	S_1	473	592	2.24
	S_2	340	463	2.91
**6**[a]	S_1	468	594	2.23
	S_2	467	511	2.88

[a] For **6** the ground-state structure is symmetric (*C_2*) and the first (second) excited-state in absorption is of *B* (*A*) symmetry. Optimization of the first (second) excited-state yields a *C_1* (*C_2*) stable geometry.

Table. S4: *Theoretical best estimates of the vertical phosphorescence wavelengths (in nm) and 0-0 energies (in eV) for all compounds. We provide both CC2 and SCS-CC2 corrected values*

		**SCS-CC2**		**CC2**	
		$\lambda^{vert\text{-}phos}$ (nm)	ΔE^{0-0} (eV)	$\lambda^{vert\text{-}phos}$ (nm)	ΔE^{0-0} (eV)
**2**	T_1	555	2.34	603	2.23
**5**	T_1	573	2.31	632	2.16
**6**	T_1	579	2.31	642	2.15

Table. S5: *Singlet-triplet gaps (eV) computed at PBE0 (left) and SCS-CC2 (center) levels. A negative value indicates a higher-lying triplet state. On the right-hand-side, we report PBE0 SOC matrix elements (cm^{-1}). All calculations on the optimal S_1 structures.*

	**PBE0**		**SCS-CC2**		**PBE0**	
	S_1-T_1 (eV)	S_1-T_2 (eV)	S_1-T_1 (eV)	S_1-T_2 (eV)	S_1-T_1 (cm^{-1})	S_1-T_2 (cm^{-1})
**2**	0.33	0.29	0.10	-0.10	0.2	80.1
**5**	0.36	0.28[a]	0.11	-0.13	37.6	75.0
**6**	0.37	0.27[b]	0.12	-0.14	40.3	73.8

[a]The third triplet is ca. 0.5 eV above the lowest singlet. [b]The third triplet is almost perfectly aligned with the lowest singlet (-0.01 eV), but the associated SOCMe is small 0.9 cm^{-1}).

f) Topological analysis

The total electron density of the S_0 and S_1 states for bisphthalimides **5** and **6**, at their S_1 geometries, was analyzed using the quantum theory of atoms in molecules (QTAIM). The calculated values of the usual descriptors for the identified S···O interaction are presented in Table S6.

Table. S6: *Selected QTAIM descriptors (a.u.) calculated at the bond critical point (BCP) of the S···O interaction identified in S_0 and S_1 states of bisphthalimides **5** and **6**.*

	**5**		**6**	
	S₀	***S₁***	***S₀***	***S₁***
ρ_{BCP}	0.007	0.006	0.008	0.007
$\nabla^2\rho_{\mathrm{BCP}}$	0.021	0.019	0.025	0.023
H_{BCP}	-0.004	-0.003	-0.005	-0.003
V_{BCP}	-0.013	-0.010	-0.016	-0.012
**DI**	0.048	0.049	0.056	0.058

The positive value of the Laplacian of the electron density at the BCP (the point of minimum electron density along the bond path), $\nabla^2\rho_{\mathrm{BCP}} \approx 0.02\ \mathrm{a.u.}$, is indicative of closed-shell interactions, that is mainly electrostatic S···O interactions. In addition, the electron density calculated at the BCP is very small, $\rho_{\mathrm{BCP}} < 0.01\ \mathrm{a.u.}$, testifying to weak non-covalent interactions in line with the small value of the delocalization index ($\mathrm{DI} \approx 0.05$). This descriptor quantifies the number of shared electron-pairs between a pair of atoms; it can be regarded as a measure of bond order. The presence of some electron sharing is confirmed by the negative value of the total energy density at the BCP, H_{BCP}, which reflects the covalency magnitude of the interaction. A negative value indicates that the potential energy density (<0) prevails over the kinetic energy density (>0) and electrons are stabilized close to the BCP.

According to the difference of ρ_{BCP} and V_{BCP} values between bisphthalimides **5** and **6**, the S···O interaction is enhanced in **6** (larger ρ_{BCP} and more negative potential energy density at the BCP, V_{BCP}).

Additional NCI (Non-Covalent Interaction) analyses in S_0 state were performed to further investigate the S···O intramolecular interactions. The results are depicted in Figure S52 and S53 for compounds **5** and **6**, respectively.

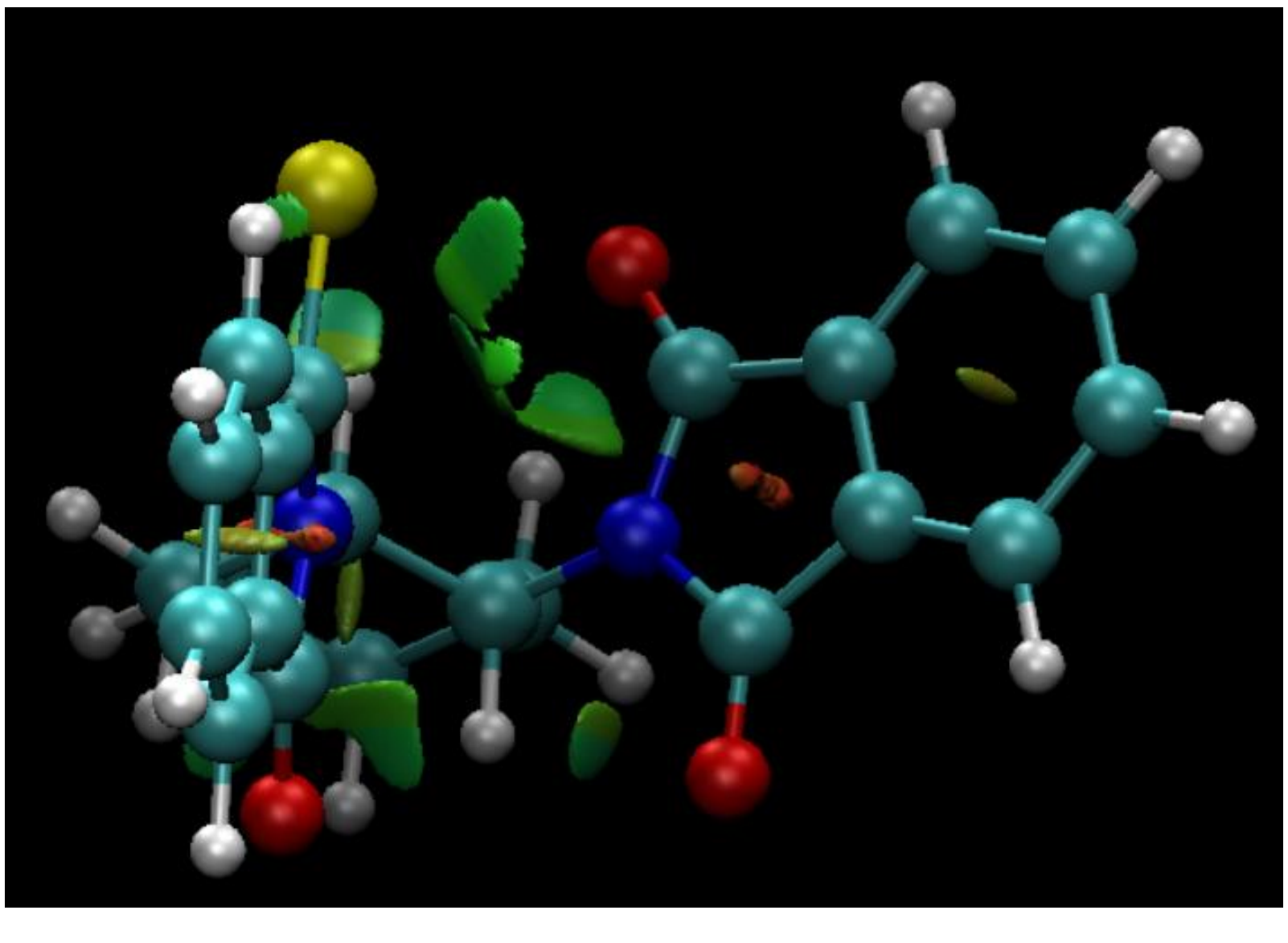

Figure S52: *NCI surface (isosurface = 0.4, range: -0.5, 0.5) for bisphthalimide **5** in the S_0 state.*

The NCI analysis reveals for **5** an extended sheet-shaped green isosurface around the S···O contact, indicating a weak non-covalent interaction resulting from van der Waals forces. On the opposite side of the molecule, no isosurface is observed between the (two) oxygen atoms, indicating that there is no interaction between them. The NCI analysis therefore suggests that the S···O contact is the most significant intermolecular interaction identified in this region for the monothione compound.

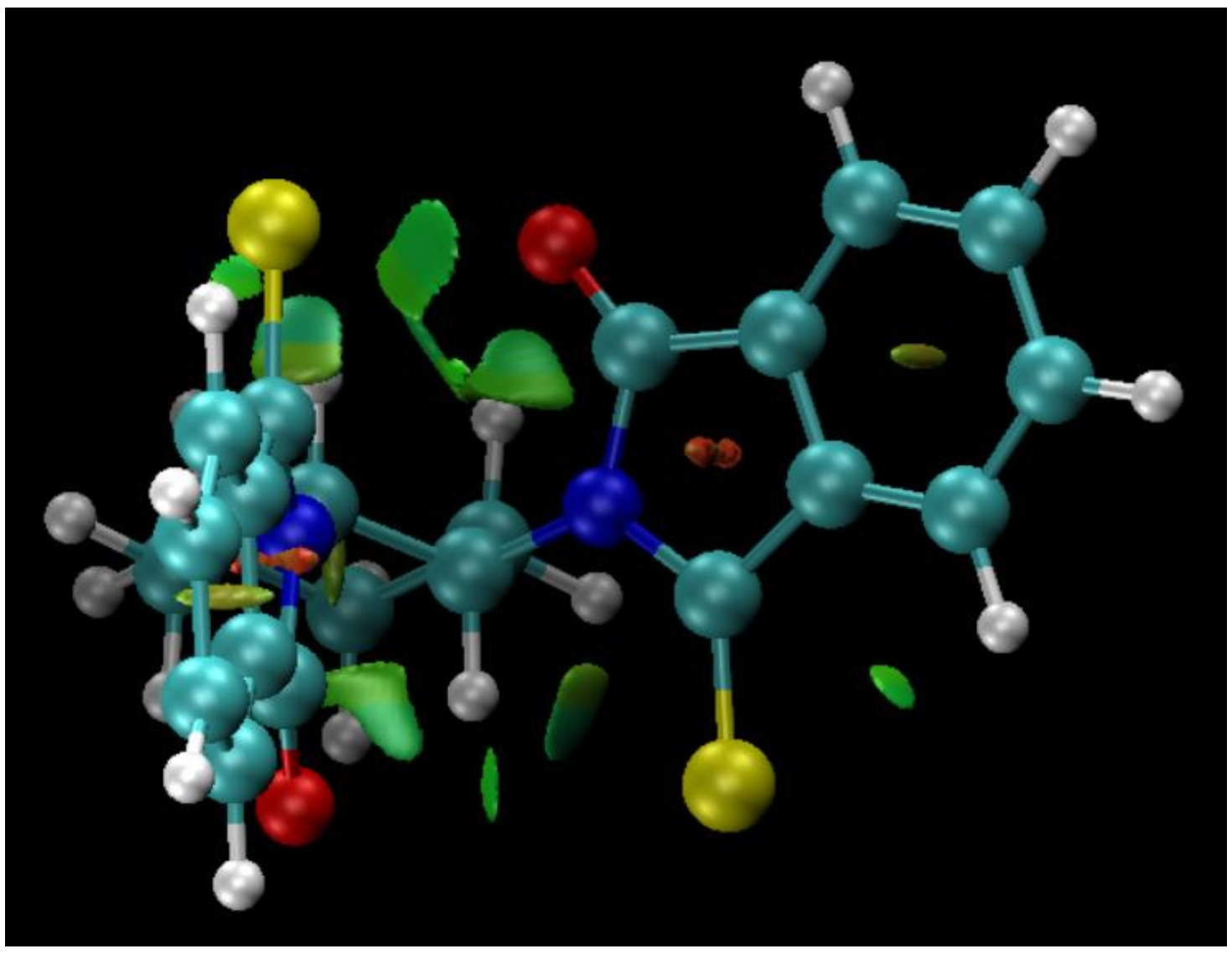

Figure S53: *NCI surface (isosurface = 0.4, range: -0.5, 0.5) for bisphthalimide **6** in the S_0 state.*

Regarding the compound **6**, the NCI analysis reveals two S···O non-covalent interactions. An extended sheet-shaped green isosurface is observed along one S···O contact, indicating a weak non-covalent interaction, similar to the van der Waals interaction previously identified in **5**. On the opposite side of the compound, a small sheet-shaped green isosurface is observed between the S and O atoms. This indicates a non-covalent interaction, but its smaller extent suggests weaker stabilizing van der Waals forces (consistent with the absence of interaction found in the QTAIM analysis). Overall, the NCI analysis indicates that both S···O contacts are weak, with little or no electron sharing, and that the first interaction predominates.

g) Cartesian coordinates

We produce below the Cartesian coordinates of the key optimized geometries of the most stable conformers. All structures are true minima. The free energies are PCM-(R/U/TD)PBE0/6-31+G(d) ones.

2

$S_0 - G$= -1069.602899 au.

C	-2.2599850	-0.3114760	-1.2696740
H	-2.0871050	-1.3930810	-1.3238570
C	-3.7625880	-0.0282520	-1.2606580
H	-4.2229030	-0.4607790	-2.1575640
H	-3.9296710	1.0579370	-1.3149300
C	-4.4278050	-0.5787020	0.0000120
H	-5.4978800	-0.3355880	0.0000120
H	-4.3528510	-1.6762810	0.0000270
N	-0.1607670	0.0569280	-0.0000070
C	-1.6134350	0.2463720	-0.0000090
H	-1.7416600	1.3352770	-0.0000270
C	-2.2599780	-0.3114370	1.2696770
H	-1.7873120	0.1425960	2.1497890
H	-2.0870940	-1.3930400	1.3238910
C	-3.7625810	-0.0282160	1.2606630
H	-4.2228890	-0.4607190	2.1575830
H	-3.9296650	1.0579750	1.3149050
C	0.7608540	1.0792810	-0.0000070
C	2.0943210	0.4459510	-0.0000020
C	3.3608380	1.0107570	0.0000000
C	4.4547250	0.1405450	0.0000040
O	-0.1112480	-2.2725740	0.0000060
C	0.4680590	-1.2055520	0.0000000
C	1.9221360	-0.9370000	0.0000010
C	2.9978580	-1.8093280	0.0000050
C	4.2782350	-1.2471870	0.0000070
H	3.4922090	2.0889950	-0.0000020
H	5.4615600	0.5488910	0.0000050
H	5.1496740	-1.8959740	0.0000110
H	2.8507090	-2.8855820	0.0000080
H	-1.7873250	0.1425280	-2.1498040
S	0.4502280	2.6946500	-0.0000100

$S_1 - G$= -1069.518894 au.

C	-2.2450930	-0.3199240	-1.2693260
H	-2.0688170	-1.4012170	-1.3170170
C	-3.7471410	-0.0350870	-1.2605900
H	-4.2063650	-0.4681030	-2.1575750
H	-3.9142040	1.0512800	-1.3171360
C	-4.4136670	-0.5838200	0.0000030
H	-5.4829520	-0.3376750	-0.0000010
H	-4.3415010	-1.6813180	0.0000160
N	-0.1410240	0.0226470	0.0000060
C	-1.5915950	0.2269290	0.0000000
H	-1.7256460	1.3227390	-0.0000120
C	-2.2450980	-0.3198960	1.2693350
H	-1.7686060	0.1302360	2.1489990
H	-2.0688230	-1.4011880	1.3170500
C	-3.7471460	-0.0350590	1.2605880
H	-4.2063740	-0.4680560	2.1575800
H	-3.9142090	1.0513090	1.3171080
C	0.7734090	1.0328690	-0.0000030
C	2.0827290	0.4750660	-0.0000020
C	3.3627730	1.0515480	-0.0000120
C	4.4586250	0.2009820	-0.0000100
O	-0.0910710	-2.3169970	0.0000220
C	0.5043740	-1.2412110	0.0000200
C	1.9274590	-0.9351750	0.0000090
C	3.0361420	-1.7767350	0.0000090
C	4.3027310	-1.2004670	0.0000000
H	3.4976800	2.1299840	-0.0000210
H	5.4600910	0.6230250	-0.0000170
H	5.1846680	-1.8347560	0.0000010
H	2.9087910	-2.8561980	0.0000180

H	-1.7685970	0.1301900	-2.1489970
S	0.3362240	2.6966930	-0.0000210

S_2 – G= -1069.490322 au.

C	-2.2599850	-0.3114760	-1.2696740
H	-2.0871050	-1.3930810	-1.3238570
C	-3.7625880	-0.0282520	-1.2606580
H	-4.2229030	-0.4607790	-2.1575640
H	-3.9296710	1.0579370	-1.3149300
C	-4.4278050	-0.5787020	0.0000120
H	-5.4978800	-0.3355880	0.0000120
H	-4.3528510	-1.6762810	0.0000270
N	-0.1607670	0.0569280	-0.0000070
C	-1.6134350	0.2463720	-0.0000090
H	-1.7416600	1.3352770	-0.0000270
C	-2.2599780	-0.3114370	1.2696770
H	-1.7873120	0.1425960	2.1497890
H	-2.0870940	-1.3930400	1.3238910
C	-3.7625810	-0.0282160	1.2606630
H	-4.2228890	-0.4607190	2.1575830
H	-3.9296650	1.0579750	1.3149050
C	0.7608540	1.0792810	-0.0000070
C	2.0943210	0.4459510	-0.0000020
C	3.3608380	1.0107570	0.0000000
C	4.4547250	0.1405450	0.0000040
O	-0.1112480	-2.2725740	0.0000060
C	0.4680590	-1.2055520	0.0000000
C	1.9221360	-0.9370000	0.0000010
C	2.9978580	-1.8093280	0.0000050
C	4.2782350	-1.2471870	0.0000070
H	3.4922090	2.0889950	-0.0000020
H	5.4615600	0.5488910	0.0000050
H	5.1496740	-1.8959740	0.0000110
H	2.8507090	-2.8855820	0.0000080
H	-1.7873250	0.1425280	-2.1498040
S	0.4502280	2.6946500	-0.0000100

T_1 – G= -1069.532206 au.

C	-2.2487190	-0.3182990	-1.2688590
H	-2.0701090	-1.3991330	-1.3163150
C	-3.7512370	-0.0361650	-1.2604990
H	-4.2100920	-0.4696550	-2.1575290
H	-3.9205340	1.0499910	-1.3165920
C	-4.4169400	-0.5861410	0.0000040
H	-5.4867990	-0.3422540	0.0000000
H	-4.3424330	-1.6834910	0.0000130
N	-0.1444520	0.0255220	0.0000050
C	-1.5949160	0.2301590	0.0000010
H	-1.7333590	1.3243670	-0.0000080
C	-2.2487220	-0.3182780	1.2688680
H	-1.7731260	0.1321840	2.1489910
H	-2.0701120	-1.3991120	1.3163420
C	-3.7512410	-0.0361440	1.2604990
H	-4.2100980	-0.4696190	2.1575350
H	-3.9205380	1.0500130	1.3165750
C	0.7823200	1.0405010	-0.0000080
C	2.0883040	0.4774050	-0.0000050
C	3.3765460	1.0393010	-0.0000160
C	4.4622280	0.1774210	-0.0000100
O	-0.0971210	-2.3109450	0.0000300
C	0.4932840	-1.2287170	0.0000200
C	1.9204800	-0.9304740	0.0000110
C	3.0208130	-1.7845030	0.0000150
C	4.2927250	-1.2228360	0.0000050
H	3.5252520	2.1160950	-0.0000280
H	5.4680780	0.5893400	-0.0000180
H	5.1684390	-1.8659190	0.0000090
H	2.8799290	-2.8623250	0.0000270
H	-1.7731200	0.1321490	-2.1489870
S	0.3567010	2.7116810	-0.0000280

5

$S_0 - G$= -1580.873368 au.

N	-1.3718080	0.6868060	0.2304770
C	-0.5400900	1.8805790	0.3862430
H	-0.1301240	1.8117490	1.3995680
C	-1.3798050	3.1577450	0.2754920
H	-2.1451730	3.1366300	1.0607930
H	-1.9000590	3.1692520	-0.6889930
C	-0.5103520	4.4059510	0.3968160
H	-1.1389710	5.2982210	0.2908140
H	-0.0597760	4.4525570	1.3987970
C	-1.5976910	-0.2503150	1.2174100
C	-2.5067440	-1.2579840	0.6382040
C	-3.0361990	-2.4151700	1.1900510
C	-3.8860050	-3.1813360	0.3877470
O	-2.1163120	1.0719910	-1.9446070
C	-2.1027470	0.3887870	-0.9420000
C	-2.8144200	-0.8761240	-0.6662590
C	-3.6566310	-1.6260670	-1.4705850
C	-4.1922900	-2.7948790	-0.9217090
N	1.4616390	0.6820660	-0.4411630
C	0.6333180	1.8720520	-0.6131070
H	0.2272140	1.7869090	-1.6263770
C	1.4654870	3.1550220	-0.5146590
H	2.2359690	3.1264640	-1.2946860
H	1.9805820	3.1805890	0.4522660
C	0.5928780	4.3986820	-0.6571940
H	1.2185720	5.2945800	-0.5653080
H	0.1424100	4.4279090	-1.6599510
O	1.0312320	-0.2855730	-2.5068440
C	1.5901950	-0.3015850	-1.4289210
C	2.5281670	-1.3179800	-0.8875060
C	2.9922900	-2.4977080	-1.4448110
C	3.8843110	-3.2580820	-0.6812090
O	2.3613710	1.0835490	1.6665230
C	2.2574420	0.3932790	0.6726650
C	2.9295640	-0.8998650	0.3777520
C	3.8123770	-1.6437340	1.1423240
C	4.2871850	-2.8383810	0.5904670
H	2.6757860	-2.8182190	-2.4331130
H	4.2716110	-4.1909210	-1.0815140
H	4.9809870	-3.4520490	1.1583540
H	-3.8882050	-1.3170480	-2.4857890
H	-2.7961130	-2.7093190	2.2077080
H	-4.3179650	-4.0945440	0.7876820
H	-4.8566050	-3.4131480	-1.5189790
H	4.1213200	-1.3126170	2.1295120
S	-0.9976270	-0.2422600	2.7462600

$S_1 - G$= -1580.789357 au.

N	-1.3978700	0.6360840	0.1914080
C	-0.5922620	1.8417720	0.3865920
H	-0.2018030	1.7861630	1.4110700
C	-1.4474600	3.1072300	0.2662220
H	-2.2479810	3.0562490	1.0140020
H	-1.9217610	3.1226000	-0.7212690
C	-0.6057320	4.3665230	0.4527030
H	-1.2447660	5.2501160	0.3378630
H	-0.2014320	4.3992630	1.4750800
C	-1.6336120	-0.3140590	1.1449960
C	-2.5263150	-1.2884100	0.6143940
C	-3.0820650	-2.4593300	1.1546780
C	-3.9341510	-3.2037740	0.3524880
O	-2.1013050	1.0513050	-2.0015270
C	-2.1232980	0.3442480	-0.9970880
C	-2.8310740	-0.8932680	-0.7124670
C	-3.6856240	-1.6513110	-1.5080240
C	-4.2361710	-2.8087930	-0.9672430
N	1.4528940	0.6942740	-0.4108590
C	0.6117430	1.8750240	-0.5731610
H	0.2308280	1.8131980	-1.5980440
C	1.4239960	3.1642180	-0.4090080
H	2.2231370	3.1658930	-1.1598720

H	1.9034070	3.1661380	0.5775030
C	0.5433020	4.4023630	-0.5505610
H	1.1531050	5.3020540	-0.4050020
H	0.1372690	4.4558600	-1.5708430
O	1.2905420	-0.0964840	-2.5877010
C	1.7296310	-0.1924560	-1.4610130
C	2.6519000	-1.2167260	-0.9068030
C	3.2221600	-2.3276840	-1.5048490
C	4.0669280	-3.1158560	-0.7158370
O	2.1143890	0.9535710	1.8009630
C	2.1480990	0.3448760	0.7477930
C	2.9057830	-0.8913900	0.4227970
C	3.7401510	-1.6637650	1.2130770
C	4.3215660	-2.7895100	0.6198100
H	3.0203110	-2.5761760	-2.5426540
H	4.5339550	-3.9970490	-1.1468660
H	4.9824650	-3.4223580	1.2055770
H	-3.9112750	-1.3407360	-2.5248230
H	-2.8565450	-2.7781540	2.1689860
H	-4.3788980	-4.1128420	0.7485290
H	-4.9066480	-3.4188680	-1.5658740
H	3.9340040	-1.4054290	2.2500510
S	-0.9416580	-0.3015690	2.7169940

S_2 – G= -1580.758629 au.

N	-1.3837910	0.6780990	0.4032150
C	-0.6318330	1.8602180	0.5394250
H	-0.0383390	1.7710610	1.4552340
C	-1.4959020	3.1343040	0.5539600
H	-2.1082590	3.0543520	1.4620970
H	-2.1734160	3.1282420	-0.3030970
C	-0.6702430	4.4071810	0.5655180
H	-1.3507550	5.2669180	0.5824090
H	-0.0589290	4.4642040	1.4769170
C	-1.1978600	-0.4846090	1.2235720
C	-2.1328290	-1.4267090	0.7285550
C	-2.4287060	-2.7327210	1.1627490
C	-3.3936800	-3.4438180	0.4814560
O	-2.6277320	1.2894590	-1.5024090
C	-2.3656020	0.4636200	-0.6220950
C	-2.8428390	-0.8639540	-0.3815940
C	-3.8168540	-1.6044210	-1.0697850
C	-4.0886190	-2.8840030	-0.6327370
N	1.3460970	0.8214110	-0.5839730
C	0.4604950	1.9342630	-0.6778300
H	-0.1265700	1.8022150	-1.5921660
C	1.1830640	3.2824490	-0.6618990
H	1.8164380	3.2900030	-1.5601170
H	1.8472500	3.3397630	0.2041370
C	0.2308650	4.4658310	-0.6635030
H	0.8156990	5.3926940	-0.6682740
H	-0.3819800	4.4666640	-1.5752950
O	0.2974200	-0.4600790	-2.2068980
C	1.1638550	-0.3414130	-1.3693340
C	2.2169400	-1.2894210	-0.9542670
C	2.5038170	-2.5632380	-1.4190180
C	3.5737200	-3.2337250	-0.8212440
O	2.7667040	1.4672990	1.1548280
C	2.4072540	0.6494060	0.3377470
C	2.9596210	-0.6971650	0.0658850
C	4.0229010	-1.3536560	0.6642420
C	4.3210230	-2.6382570	0.2027930
H	1.9185150	-3.0199270	-2.2114180
H	3.8309160	-4.2353980	-1.1536190
H	5.1458120	-3.1882910	0.6468800
H	-4.3403650	-1.1697160	-1.9165130
H	-1.8981510	-3.1594860	2.0090120
H	-3.6345330	-4.4570410	0.7920380
H	-4.8427660	-3.4819510	-1.1367520
H	4.5957580	-0.8892080	1.4611350
S	-0.1321950	-0.5678790	2.5133100

$T_1 - G$= -1580.803397au.

N	-1.4054150	0.6336710	0.1497260
C	-0.6273970	1.8526410	0.3584940
H	-0.2884500	1.8240670	1.4010700
C	-1.4818090	3.1093600	0.1674130
H	-2.3365280	3.0552610	0.8523480
H	-1.8797670	3.1191340	-0.8535480
C	-0.6626350	4.3730220	0.4189300
H	-1.2931550	5.2550010	0.2548750
H	-0.3417840	4.4051720	1.4706250
C	-1.6821770	-0.3053990	1.1201150
C	-2.5270450	-1.3054460	0.5633010
C	-3.0956700	-2.4784020	1.0897010
C	-3.8851450	-3.2507990	0.2524740
O	-1.9811430	0.9748600	-2.0883470
C	-2.0494990	0.3008590	-1.0595100
C	-2.7594170	-0.9409580	-0.7861870
C	-3.5505850	-1.7294670	-1.6184750
C	-4.1146350	-2.8852400	-1.0905920
N	1.4517170	0.7047810	-0.3674390
C	0.6232410	1.8928570	-0.5382350
H	0.2947320	1.8592290	-1.5823690
C	1.4287900	3.1737840	-0.2975860
H	2.2809220	3.1836000	-0.9875510
H	1.8348100	3.1580950	0.7220830
C	0.5637830	4.4169760	-0.4883050
H	1.1611690	5.3135200	-0.2836380
H	0.2407940	4.4840000	-1.5370610
O	1.5512510	0.0900280	-2.6040370
C	1.8601910	-0.0937410	-1.4456850
C	2.7298020	-1.1524000	-0.8713610
C	3.3811830	-2.2091780	-1.4843350
C	4.1446020	-3.0503440	-0.6674790
O	1.8576060	0.7966510	1.9168900
C	2.0191220	0.2721080	0.8294980
C	2.8276500	-0.9299410	0.4995330
C	3.5804990	-1.7552930	1.3176190
C	4.2427070	-2.8271960	0.7093860
H	3.3009470	-2.3776200	-2.5542490
H	4.6710090	-3.8918540	-1.1091970
H	4.8440710	-3.4986510	1.3159370
H	-3.7158530	-1.4416860	-2.6534610
H	-2.9229710	-2.7779010	2.1201870
H	-4.3364610	-4.1613700	0.6379320
H	-4.7373840	-3.5182940	-1.7165390
H	3.6532890	-1.5768470	2.3864970
S	-1.1230350	-0.2213210	2.7482320

6

$S_0 - G$= -1903.749274 au.

N	0.6366990	1.3172980	0.6780100
C	-0.0198660	0.7707600	1.8663950
H	-1.0631180	1.0927690	1.7879360
C	0.5834570	1.3561530	3.1483430
H	0.4528370	2.4448070	3.1222880
H	1.6604240	1.1556000	3.1697730
C	-0.0666150	0.7599070	4.3933310
H	0.4018180	1.1879770	5.2876670
H	-1.1305720	1.0351810	4.4295380
C	0.0198660	2.1113500	-0.2671620
C	1.0516300	2.4533410	-1.2649870
C	0.9654050	3.2051950	-2.4274720
C	2.1317440	3.3627410	-3.1809240
O	2.7980960	0.5535320	1.0991180
C	2.0136030	1.1604320	0.4005010
C	2.2578470	1.8811820	-0.8654890
C	3.4210950	2.0324210	-1.6022060
C	3.3410100	2.7864430	-2.7766040
N	-0.6366990	-1.3172980	0.6780100
C	0.0198660	-0.7707600	1.8663950
H	1.0631180	-1.0927690	1.7879360
C	-0.5834570	-1.3561530	3.1483430

H	-0.4528370	-2.4448070	3.1222880
H	-1.6604240	-1.1556000	3.1697730
C	0.0666150	-0.7599070	4.3933310
H	-0.4018180	-1.1879770	5.2876670
H	1.1305720	-1.0351810	4.4295380
O	-2.7980960	-0.5535320	1.0991180
C	-2.0136030	-1.1604320	0.4005010
C	-2.2578470	-1.8811820	-0.8654890
C	-3.4210950	-2.0324210	-1.6022060
C	-3.3410100	-2.7864430	-2.7766040
C	-0.0198660	-2.1113500	-0.2671620
C	-1.0516300	-2.4533410	-1.2649870
C	-0.9654050	-3.2051950	-2.4274720
C	-2.1317440	-3.3627410	-3.1809240
H	-4.3549950	-1.5815770	-1.2791170
H	-4.2296930	-2.9284720	-3.3852000
H	-2.1000960	-3.9439870	-4.0983380
H	4.3549950	1.5815770	-1.2791170
H	0.0244600	3.6518570	-2.7356080
H	2.1000960	3.9439870	-4.0983380
H	4.2296930	2.9284720	-3.3852000
H	-0.0244600	-3.6518570	-2.7356080
S	-1.5454780	2.6081350	-0.2737700
S	1.5454780	-2.6081350	-0.2737700

$S_1 - G$= -1903.667344 au. (the molecule breaks sym in the lowest excited-state)

N	-1.4591000	0.6265710	0.2284600
C	-0.6521450	1.8186930	0.4889940
H	-0.3360210	1.7496390	1.5370180
C	-1.4795110	3.0974970	0.3195920
H	-2.3384820	3.0446840	0.9992020
H	-1.8718320	3.1348830	-0.7028270
C	-0.6409100	4.3421410	0.5973830
H	-1.2578710	5.2363300	0.4489200
H	-0.3206970	4.3498400	1.6496150
C	-1.8036810	-0.3064790	1.1667040
C	-2.6748060	-1.2611680	0.5682430
C	-3.3058390	-2.4117030	1.0679010
C	-4.1067860	-3.1408990	0.2016040
O	-1.9781380	1.0507980	-2.0129810
C	-2.0961710	0.3481230	-1.0123450
C	-2.8562790	-0.8695450	-0.7817550
C	-3.6609730	-1.6119020	-1.6416170
C	-4.2854940	-2.7500160	-1.1416070
N	1.4340820	0.6598910	-0.2130400
C	0.6130870	1.8568060	-0.3877050
H	0.2988030	1.8372770	-1.4364000
C	1.4334960	3.1254670	-0.1297370
H	2.2863520	3.1294300	-0.8189860
H	1.8369650	3.0952780	0.8900090
C	0.5859170	4.3818150	-0.3088650
H	1.1958570	5.2670380	-0.0925590
H	0.2647990	4.4659250	-1.3568770
O	1.7907130	0.8197090	2.0813630
C	1.9754440	0.2521350	1.0217520
C	2.7760700	-0.9554010	0.7389310
C	3.4999520	-1.7737560	1.5906310
C	4.1687460	-2.8600440	1.0189250
C	1.8753760	-0.1430620	-1.2480160
C	2.7105180	-1.1921050	-0.6333710
C	3.3735140	-2.2665550	-1.2074650
C	4.1049450	-3.1010060	-0.3579390
H	3.5444770	-1.5773540	2.6579870
H	4.7475350	-3.5274910	1.6512160
H	4.6353360	-3.9531750	-0.7738010
H	-3.7926180	-1.3043560	-2.6757140
H	-3.1749420	-2.7263900	2.1000020
H	-4.6075590	-4.0345200	0.5645670
H	-4.9189600	-3.3478750	-1.7907510
H	3.3219920	-2.4478760	-2.2771480
S	-1.2642020	-0.2917840	2.7962730
S	1.5663620	0.0521100	-2.8468910

S_2 – G= -1903.646149 au.

N	1.2467150	0.7664770	0.6579830
C	0.4034150	0.6563450	1.8489390
H	-0.3062500	1.4888540	1.7885150
C	1.2344750	0.8077240	3.1278210
H	1.7289490	1.7862230	3.1021390
H	2.0203790	0.0438660	3.1393840
C	0.3675910	0.6684270	4.3759440
H	0.9981880	0.7611870	5.2681910
H	-0.3631430	1.4890610	4.4174100
C	1.1878610	1.7913520	-0.2564850
C	2.2234800	1.5588080	-1.2431770
C	2.5905910	2.2752880	-2.3828220
C	3.6513960	1.7862650	-3.1405850
O	2.5905910	-1.1058060	1.0238100
C	2.3000280	-0.1279540	0.3514650
C	2.9062440	0.3860110	-0.8796750
C	3.9641950	-0.1014650	-1.6353230
C	4.3318350	0.6132560	-2.7750740
N	-1.2467150	-0.7664770	0.6579830
C	-0.4034150	-0.6563450	1.8489390
H	0.3062500	-1.4888540	1.7885150
C	-1.2344750	-0.8077240	3.1278210
H	-1.7289490	-1.7862230	3.1021390
H	-2.0203790	-0.0438660	3.1393840
C	-0.3675910	-0.6684270	4.3759440
H	-0.9981880	-0.7611870	5.2681910
H	0.3631430	-1.4890610	4.4174100
O	-2.5905910	1.1058060	1.0238100
C	-2.3000280	0.1279540	0.3514650
C	-2.9062440	-0.3860110	-0.8796750
C	-3.9641950	0.1014650	-1.6353230
C	-4.3318350	-0.6132560	-2.7750740
C	-1.1878610	-1.7913520	-0.2564850
C	-2.2234800	-1.5588080	-1.2431770
C	-2.5905910	-2.2752880	-2.3828220
C	-3.6513960	-1.7862650	-3.1405850
H	-4.4847520	1.0095930	-1.3440200
H	-5.1550580	-0.2604930	-3.3899160
H	-3.9598160	-2.3226640	-4.0338510
H	4.4847520	-1.0095930	-1.3440200
H	2.0668710	3.1828940	-2.6698930
H	3.9598160	2.3226640	-4.0338510
H	5.1550580	0.2604930	-3.3899160
H	-2.0668710	-3.1828940	-2.6698930
S	0.1269160	3.0880480	-0.2137800
S	-0.1269160	-3.0880480	-0.2137800

T_1 – G= -1903.680061 au. (most stable solution)

N	1.4663930	0.6408200	-0.1987400
C	0.6761640	1.8342720	-0.4898970
H	0.4133600	1.7737480	-1.5515170
C	1.4832620	3.1182620	-0.2726840
H	2.3889270	3.0655580	-0.8887480
H	1.8011540	3.1708900	0.7745440
C	0.6549230	4.3503320	-0.6298940
H	1.2490720	5.2541970	-0.4500230
H	0.4154090	4.3381580	-1.7034210
C	1.8283920	-0.3148150	-1.1245860
C	2.6703430	-1.2706980	-0.4907750
C	3.3048740	-2.4382710	-0.9496570
C	4.0669340	-3.1621670	-0.0462270
O	1.8984630	1.0603670	2.0577650
C	2.0483480	0.3614160	1.0547320
C	2.8115370	-0.8631330	0.8588860
C	3.5752860	-1.6034790	1.7582560
C	4.2048220	-2.7540480	1.2971140
N	-1.4232390	0.6570710	0.1603700
C	-0.6301200	1.8741980	0.3232010
H	-0.3659080	1.9002950	1.3854400

C	-1.4539890	3.1181650	-0.0243480
H	-2.3519800	3.1272010	0.6048580
H	-1.7863420	3.0521290	-1.0685540
C	-0.6383440	4.3925260	0.1794060
H	-1.2418830	5.2625940	-0.1055770
H	-0.3989420	4.5084360	1.2460510
C	-1.9597410	-0.0676250	1.2090350
C	-2.7311430	-1.1683010	0.6017370
C	-3.4451680	-2.2005630	1.1915580
C	-4.0893120	-3.1054470	0.3433350
O	-1.5479240	0.6276260	-2.1625220
C	-1.8391300	0.1472210	-1.0820350
C	-2.6632220	-1.0403760	-0.7849510
C	-3.2990310	-1.9296790	-1.6358940
C	-4.0189340	-2.9737920	-1.0478920
H	-3.4974400	-2.2975780	2.2720980
H	-4.6564440	-3.9271920	0.7717000
H	-4.5322970	-3.6940250	-1.6787460
H	3.6696050	-1.2833590	2.7926510
H	3.2024060	-2.7703210	-1.9796350
H	4.5679930	-4.0676660	-0.3785160
H	4.8082120	-3.3500190	1.9761770
H	-3.2394170	-1.8181490	-2.7146900
S	1.3705000	-0.2938460	-2.7861290
S	-1.8012740	0.2527170	2.8093820
